# Linear regression in ecological studies involving space: methodology and an application example in public health

**DOI:** 10.1590/1980-549720260018

**Published:** 2026-04-20

**Authors:** Gleice Margarete de Souza Conceição, Patricia Marques Moralejo Bermudi, Raquel Gardini Sanches Palasio, Gerson Laurindo Barbosa, Camila Meireles Fernandes, Lidia Maria Reis Santana, Ligia Vizeu Barrozo, Daiane Leite da Roza, José Alberto Quintanilha, Francisco Chiaravalloti-Neto

**Affiliations:** I Universidade de São Paulo, School of Public Health, Department of Epidemiology - São Paulo (SP), Brazil.; II Instituto Pasteur, São Paulo State Health Secretariat - São Paulo (SP), Brazil.; III Universidade Federal de São Paulo, Department of Medicine - São Paulo (SP), Brazil.; IV Universidade de São Paulo, School of Philosophy, Languages and Human Sciences - São Paulo (SP), Brazil.; V Universidade de São Paulo, Institute of Energy and Environment - São Paulo (SP), Brazil.

**Keywords:** Linear regression, Ecological studies, Spatial dependence, Spatial analysis, Spatial autocorrelation, Autoregressive models

## Abstract

Many health-related phenomena can be better understood when the geographic region in which they occur is taken into account. One of the most important aspects to consider in spatial study designs is the presence of autocorrelation in observations measured across space. If this spatial dependence is not properly modeled, the resulting statistics may be biased, compromising the validity of conclusions regarding the presence or absence of associations. Methodologies developed based on the linear regression model allow this dependence to be adequately accommodated, producing precise, robust, and unbiased estimates. With the aim of highlighting the applicability of spatial models and pointing out the necessary precautions in data analysis, this article describes, step by step, one of the most commonly used methodologies for spatial data analysis, as well as the measures to be taken to avoid modeling errors and distortion of results. The linear regression model is presented, along with procedures to evaluate model fit, the most commonly used measure to detect spatial dependence, and two autoregressive models frequently applied to model this dependence (SAR and SEM). An application example is provided using the GeoDa and R software.

## INTRODUCTION

The term “regression” was introduced in 1885 by Francis Galton. While studying the anthropometric characteristics of successive generations, Galton observed that children of parents who were taller than the average tended to be tall but shorter than their parents, whereas children of parents who were shorter than the average tended to be short but taller than their parents. Based on these observations, he postulated that human height tended to “regress” toward the mean^1^.

The Least Squares Method, developed by Legendre and Gauss to determine the orbits of comets, made it possible to estimate the parameters of the regression proposed by Galton. Gauss noted that this method provides optimal estimates when the errors are assumed to be random, independent, and normally distributed^2^ ^,^ ^3^. This methodology was later refined and applied in the formulation of the theory of Linear Regression Models (LRMs). Technological advances have substantially reduced computational time and effort, enabling the technique to be applied to increasingly large datasets. Currently, LRMs are widely used for data analysis across a broad range of fields.

Based on this theory, new methodologies have been developed, including models for the analysis of spatial and temporal data. These approaches help establish relationships between variables of interest and interrelated factors across time and space, allowing for a deeper understanding of the phenomena under study and enabling the formulation of more effective public policies^4^.

It is common for observations measured across space and/or time to be correlated, making it necessary to account for this dependence. Spatial regression models allow this dependence to be incorporated, generating accurate, robust, and unbiased estimates, as well as enabling the identification and interpretation of the effects of spatial factors that influence the phenomenon under study.

On the other hand, disregarding the spatial dimension can compromise the validity of the conclusions. Among the main problems resulting from this are inadequate model specification, the omission of important variables, and the production of biased estimates and incorrect confidence intervals and *p*-values, which can lead to spurious associations and inaccurate or invalid conclusions^5^.

Thus, spatial regression models are powerful tools for analyzing data observed in space. Software such as GeoDa and the R language have facilitated their application. However, it is important to emphasize that the success of these applications depends on knowledge of the theoretical foundations and a clear understanding of the problem under investigation.

Although the international scientific literature includes studies using a spatial approach, this perspective is less frequent in Brazilian journals. A search in the Virtual Health Library (VHL), Public Health Brazil^6^, using the term “linear regression” identified 2,458 articles in Portuguese published up to August 2025. When the search was restricted to “linear regression” and “spatial,” the number was reduced to only 82.

According to Figueiredo Filho et al.^7^, supporting materials that contribute to the understanding of spatial and spatiotemporal models, highlighting appropriate procedures and practices to be avoided, are also scarce in Brazilian scientific journals. The present study aimed to address this gap by offering a comprehensive approach to the relevance and application of spatial models. The objective is to disseminate tools that assist in selecting the most appropriate method for the analysis of spatial data, as well as to discuss the biases that may arise when spatial dependence is ignored, thereby reducing the risk of erroneous conclusions and promoting the advancement of regression methods in spatial data analysis. To this end, LRM and its assumptions are presented, along with the most widely used measure for detecting spatial dependence in model residuals and two of the most commonly applied approaches for modeling this dependence. As an example, an application is presented using data from Fernandes et al.^8^, who evaluated the spatial distribution of the prevalence of adolescent mothers and its relationship with socioeconomic indicators in the municipality of Foz do Iguaçu, Paraná (PR).

As this was a methodological study, with no direct involvement of human participants or use of identifiable individual data, submission to a Research Ethics Committee was not required.

## THE LINEAR REGRESSION MODEL

LRMs are used to describe and quantify linear or linearizable associations and to generate forecasts. They can be applied to assess trends in spatial, temporal, or spatiotemporal series. These models involve a quantitative response variable (*Y*), preferably continuous, and one or more explanatory variables (*X*_*1*_ , *X*_*2*_ ,..., *X*_*p*_ ), also known as covariates, which may be quantitative or qualitative. The objective is to evaluate the joint effect of the covariates on the response variable (RV) and to approximate this relationship using a mathematical function, thereby facilitating its description and quantification and, eventually, enabling forecasting^9^.

Such models can be applied in spatial ecological designs, in which the geographic region where events occur is fundamental to understanding the phenomenon under study. Therefore, both the database structure and the adopted model must take into account the spatial distribution of the data. In this type of design, the study area is partitioned into Spatial Area Units (SAUs), which are generally based on pre-existing units, such as municipalities and census tracts.

The LRM model for spatial data analysis is similar to the classical model, except that the variables are measured at the SAU level, and each row of the database refers to one of these units. The model can be written as^10^

Y=Xβ+ε

Where:

*Y* = (*y*_*1*_ , *y*_*2*_ ,..., *y*_*N*_ ) is the vector *(Nx1)* containing the RV values for each SAU;

N is the number of SAUs;

*X* is the matrix *(Nxp)* containing the values of the *p* covariates for each SAU;

β is the parameter vector *(px1)* to be estimated, which will quantify the effect of each explanatory variable on the response;

ε is the vector *(Nx1)* of random errors.

The model assumptions are that ε~*N*(0,σ^2^ *I*_*N*_ ), where *I*_*N*_ is the identity matrix, and that (ε_*i*_ , ε_*j*_ ) are independent for every pair of SAUs (*i, j*). These assumptions can be translated into four essential conditions: normality: random errors with a normal distribution, implying normality of the RV; homoscedasticity: constant variance of the RV across the values of the covariates; linearity: a linear relationship between the RV and the covariates; independence: RV values that are independent of each other (uncorrelated)^10^.

These assumptions must be verified before and after model fitting, through exploratory and residual analyses, respectively. If they are not satisfied, the results may be compromised. In the absence of linearity, the model will not adequately reflect the behavior of the data, and the estimates will be invalid. In the absence of normality, homoscedasticity, or independence, the variances of the estimators, the test statistics, and the p-values of the hypothesis tests will be biased, thereby compromising any conclusions regarding the presence or absence of associations. Finally, the presence of outliers may lead to biased estimators and variances if their influence is substantial^10^.

To address problems related to normality, linearity, and homoscedasticity, several measures may be adopted, including transformations of the RV (such as logarithmic transformation); or the fitting of generalized linear, generalized additive, or Bayesian models. These approaches allow the use of distributions other than the normal, such as Poisson and negative binomial, as well as alternative functional forms for the relationship between the RV and the covariates, beyond linearity^11^. If the observations are not independent and the data do not permit adequate specification of the spatial dependence structure, little can be done, which compromises the performance of any analysis. Conversely, when such a structure exists (for example, in spatial contexts), models that explicitly incorporate this dependence should be employed. Models with spatial dependence fall within this category.

In spatial studies, the first law of Geography is commonly applied^12^: in space, all things are related, but those that are spatially closer are more related than those that are farther apart. This phenomenon is referred to as spatial dependence or positive spatial autocorrelation^4^.

It is possible that deviations from the assumptions of normality and homoscedasticity, as well as the presence of spatial dependence in the residuals of the model, may be corrected after the inclusion of covariates. If the residuals of the fitted model are normally distributed, random, homoscedastic, present few outliers (provided they do not distort the coefficient estimates), and are free from spatial dependence, the model may be considered well fitted. An example is the study by Diniz et al.^13^, which spatially modeled breast cancer mortality in the municipalities of São Paulo. Although the rates exhibited spatial dependence, this pattern was adequately accommodated by the covariates, and the residuals did not indicate any deviation from the assumptions of the LRMs.

## EXPLORATORY ANALYSIS

The first step in any statistical analysis is data exploration, which must be thorough and precede the application of any model. It is necessary to understand the relationship between the RV and each covariate, as well as the relationships among the covariates themselves. Below are the steps that should be considered in an exploratory analysis for the fitting of any LRM, in order to avoid commonly committed errors.

Initially, each variable should be described individually, according to its nature. Qualitative variables should be summarized using frequency tables and bar graphs. For quantitative variables, numerical summary measures (mean, median, standard deviation, etc.) and a set of graphical displays (histograms, box plots, and dot plots) should be used. These provide information on the range of values, their frequency and distribution, central tendency and variability, as well as the presence of asymmetry and tails. If outliers are present, it is essential to assess their influence on LRM estimates and, if necessary, apply appropriate control measures; they should be controlled for but never deleted. To evaluate the adherence of the data to a given distribution, in addition to histograms and normality tests, QQ-plots should be used. Only after understanding the behavior of each variable individually should their joint behavior be examined pairwise, and the presence of associations assessed, both between the RV and each covariate and among the covariates themselves.

To describe the relationship between RV and qualitative covariates, the response should be summarized according to the categories of the qualitative covariate, using the numerical summary measures and graphical displays mentioned previously. In particular, it is desirable that the variance of the RV be similar across the categories of the qualitative covariates. This can be assessed using tests for equality of variances^10^.

For quantitative covariates, the scatter plot and the correlation coefficient should be examined jointly. The former reveals the shape of the relationship and, if it is linear, the latter expresses its strength. Two points should be considered. The first concerns linearity, which is one of the assumptions of LRMs. If the shape of the relationship between the RV and the covariate is not linear, the LRM will not be appropriate. In such cases, it may be convenient to transform the quantitative variable into a qualitative one (for example, using age ranges instead of age). The values defining the categories depend on the researcher’s knowledge, and in the absence of prior knowledge, it is advisable to define them so that each category contains approximately the same number of observations. Another alternative is the use of polynomial terms or smoothers^10^ ^,^ ^14^. The second point refers to the fact that the correlation coefficient measures only linear associations. Therefore, it should always be interpreted in conjunction with the scatter plot, never in isolation. Furthermore, a high coefficient does not necessarily imply a linear association; similarly, a low coefficient does not necessarily imply the absence of association, since this may occur when the relationship is non-linear.

It is also necessary to understand the relationships among the covariates (using the same tools described previously), as well as to assess the presence of collinearity, which occurs when covariates are strongly correlated. Collinear covariates make the coefficients unstable and lead to imprecise estimates. For example, income and education may be collinear: higher levels of education are generally associated with higher income. When one of these variables is known, the additional information provided by the other may be limited. One possible criterion is to consider two variables collinear when the correlation between them is greater than 0.70 (or less than -0.70)^15^ and to discard one of them. Stepwise forward modeling procedures^16^ provide robust criteria for the inclusion and exclusion of collinear covariates, provided they are applied appropriately. The criterion for inclusion/retention of variables should never be based exclusively on statistical significance, but rather on the researcher’s overall knowledge of conceptual relationships and biological plausibility. Furthermore, such decisions should never be delegated to the software but should always remain under the responsibility of the researcher.

It is also essential to assess the presence of interactions between covariates, a topic not addressed in this article. However, it is important to emphasize that omitting a relevant interaction will result in an incorrectly specified model, which may lead to erroneous conclusions.

Finally, it is necessary to assess whether the RV is randomly distributed in space or whether spatial dependence is present. This can be evaluated using indices that measure the degree of spatial autocorrelation, among which the global Moran’s *I* index, presented below, is the most widely used.

Zuur et al.^17^ propose a protocol for exploratory analysis using R, which may be useful but insufficient for conducting a comprehensive exploratory analysis.

## RESIDUAL ANALYSIS

This procedure is performed after model fitting in order to verify whether the adopted model is appropriate for the data. To this end, it is necessary to examine the residuals. The residual (*e*_*i*_ ) is defined as

$$e_{i}=Y_{i}-{\hat{Y}}_{i}$$

That is, the difference between the observed value (*Y*_*i*_ ) and the corresponding fitted value (*Ŷ*_*i*_ ).

The residual *e*_*i*_ can be interpreted as the observed error, in contrast to the true error ε_*i*_ , which is unknown and unobservable. The LRM assumes that ε_*i*_ are independent and identically distributed, distributed as *N*(0,σ^2^). If the fitted model is appropriate, *e*_*i*_ should reflect the properties assumed for ε_*i*_ ; that is, they should follow a normal distribution with mean zero and variance σ^2^, present no outliers, and be independent (without autocorrelation). Residual analysis involves the construction of graphical and numerical diagnostic tools, fundamentally:

1. Scatter plot of residuals versus each covariate;
2. Scatter plot of residuals versus fitted values;
3. Histograms of residuals;
4. QQ-plot;
5. Moran’s I index.

Standardized residuals should be used, as they are more efficient than raw residuals, particularly for detecting outliers. In the diagrams listed as items 1 and 2, the residuals should be randomly dispersed around zero, with constant variance, no systematic patterns, and no influential outliers, with at least 95% of the values falling within ±1.96. Items 3 and 4 (histogram and QQ-plot) should indicate an approximately normal distribution. Moran’s I index, listed as item 5, should indicate the absence of spatial autocorrelation.

## GLOBAL MORAN INDEX (MORAN I)

Moran’s I^18^ measures the correlation between the values of a variable and the values observed in its neighborhoods. Cliff and Ord^19^ were the first to apply this index to regression residual analysis. To construct it, it is necessary to identify, for each SAU, its respective neighbors. The definition of neighborhood may be established by contiguity or by distance. The contiguity criterion, more commonly used in ecological designs, defines as neighbors the areas that share a common side (rook) or a common side or vertex (queen). The distance criterion considers the distance between the centroid of a SAU and those of neighboring areas and can be constructed in two ways: by defining as neighbors all units within a previously fixed maximum distance, or by defining as neighbors the k closest units. In both cases, the parameters are defined by the researcher.

The index can be constructed using the RV or the residuals of the fitted LRM and is calculated as:

$$I=\frac{N}{W}\frac{\sum_{i=1}^{N}\sum_{j=1}^{N}w_{ij}\left(y_{i}-\overset{-}{Y}\right)\left(y_{j}-\overset{-}{Y}\right)}{\sum_{i=1}^{N}{\left(y_{i}-\overset{-}{Y}\right)}^{2}}$$

Where:

N is the number of SAUs;

*y*_*i*_ and *y*_*j*_ are the values of the RV (or of the regression model residual) for the pair (i, j) of SAUs, *i*=1,...,*N*, *j*=1,...,*N*;

*W*_*ij*_ are the spatial weights, which take the value 1 if *i* and *j* are neighboring SAUs, and 0, otherwise;

$W=\sum_{i=1}^{N}\sum_{j=1}^{N}w_{ij}$, that is, the sum of the weights *W*_*ij*_

$\overset{-}{Y}=\frac{\sum_{i=1}^{N}y_{i}}{N}$ is the sample mean of *Y*.

Other weighting schemes for *W*_*ij*_ may also be used, for example, , where *l*_*ij*_ represents the length of the boundary between *A*_*i*_ and *A*_*j*_ , and *l*_*i*_ represents the perimeter of *A*_*i*_ .

Moran’s I ranges from -1 to 1. Values close to zero suggest the absence of spatial autocorrelation, indicating that the RV (or residuals) are randomly distributed in space, without clustering patterns. Values greater than zero indicate the presence of positive spatial autocorrelation, meaning that areas with high residual (or RV) values are geographically close to each other, as are areas with low values. Values less than zero indicate negative spatial autocorrelation, suggesting that areas with similar values are spatially dispersed and distant from each other. The hypothesis test for the index (in which Ho represents the absence of spatial autocorrelation) is performed using a Monte Carlo approach, based on a large number of simulations to obtain the p-value^4^. If Moran’s I applied to the LRM residuals indicates the presence of spatial dependence, it will be necessary to fit a model capable of accommodating this structure.

Furthermore, it is important to distinguish between the use of Moran’s I applied to the RV and that applied to the residuals. Moran’s I calculated for the response variable identifies spatial autocorrelation prior to multiple adjustment, whereas Moran’s I for the residuals assesses whether autocorrelation persists after adjustment. Thus, a significant Moran’s I in the residuals indicates that the multiple model did not fully capture the spatial structure and, therefore, that a spatial model may be required.

## LRM WITH GLOBAL SPATIAL DEPENDENCE

Spatial dependence may be local or global. If spatial autocorrelation is heterogeneous, that is, if it varies across space, the dependence is local and requires a multiparameter model to accommodate it. Geographically weighted regression^20^ is one possible approach; however, if there is no significant heterogeneity, the dependence may be assumed to be global and can be accommodated by a single parameter added to the LRM. In this article, only models with global effects are considered. The two most commonly used are the Spatial Autoregression (SAR) and the Spatial Error (SEM) Models. In the former, dependence is explicitly modeled in the RV by incorporating a spatial lag term; in the latter, it is treated as noise to be removed and modeled as a component of the residuals^21^ ^,^ ^22^.

The SAR model is formulated as:

u=λWu+ε

Where:

*Y*, *X*, β, ε are the same as previously defined;

*W* is the neighborhood matrix containing the weights *W*_*ij*_ defined previously

(*W*_*ij*_ =1, if *i* and *j* are neighboring SAUs and *W*_*ij*_ =0, otherwise);

ρ is the spatial autoregressive coefficient to be estimated.

Spatial dependence is accommodated by the term ρ*WY*, where *WY* corresponds to the spatially lagged RV and ρ corresponds to the spatial autoregressive coefficient associated with the lag.

SEM is formulated as:

Y=Xβ+u

u=λWu+ε

Where:

*Y*, *X*, β, *W,* ε are the same as previously defined;

λ is the spatial autoregressive coefficient to be estimated.

Here, spatial dependence is accommodated in the error term *u*, which is decomposed into λ*Wu* (spatially lagged error corresponding to the component of the residuals with spatial dependence) and ε (normally distributed, independent errors with constant variance).

In these models, values of ρ or λ close to zero indicate the absence of spatial autocorrelation; conversely, positive and statistically significant values indicate the presence of spatial dependence.

The choice of the most appropriate model is made using Lagrange Multiplier (LM) diagnostics, which test the hypotheses H_*0*ρ**_: *ρ*=0 and H_*0*λ**_: λ=0. The corresponding test statistics (*LM*_*ρ*_ and *LM*_*λ*_ , respectively) are obtained using Lagrange multipliers^22^. If both hypotheses are rejected, robust statistics are used, and the most suitable model is the one with the highest value of the robust LM statistic (*RLM*_*ρ*_ or *RLM*_*λ*_ ).

Once the most appropriate model has been identified, a new residual analysis is required, following items 4 and 5. If Moran’s I still indicates the presence of spatial dependence in the residuals, this suggests that the parameters ρ or λ were not able to fully capture the dependence. Before considering alternative types of models, it is recommended to select a more appropriate neighborhood matrix or include covariates that were initially omitted.

**Figure 1.**
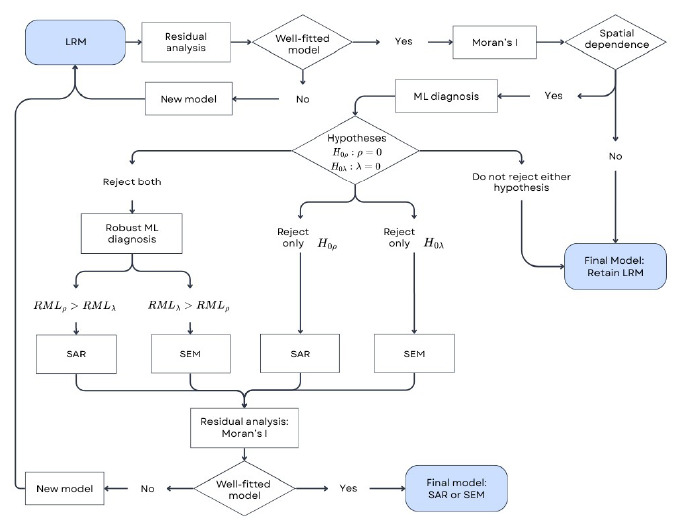
Workflow of procedures for fitting an LRM with a global spatial component^22^.

LRM: Linear Regression Model; SAR: Spatial Autoregression; SEM: Spatial Error Model

These procedures are shown in Figure 1.

## APPLICATION EXAMPLE

The study by Fernandes et al*.* ^*8*^ used the urban census tracts of the municipality of Foz do Iguaçu as SAUs (data available at https://doi.org/10.6084/m9.figshare.27310467.v2)^23^. After exploratory analysis, an LRM was fitted, with the prevalence of adolescent mothers as the RV and the Brazilian Deprivation Index (*Índice Brasileiro de Privação* - IBP) and the proportion of women heads of household (PWHH) as covariates. Moran’s I (estimated using the Queen neighborhood matrix) for the residuals was 0.059 (p=0.025), indicating the presence of spatial dependence.

Based on the LRM diagnostics, a SAR adjustment was selected. The new residual analysis indicated a well-fitted model, and Moran’s I (0.011, p=0.915) showed that spatial dependence was adequately modeled. The estimates for both models are presented in Table 1. Notably, in the SAR model, there was a decrease in the effect of IBP and an increase in the width of its confidence interval. The modeling procedures, step by step, using the GeoDa^22^ software and the R codes^24^, are presented in the supplementary material (https://github.com/LAES-USP/material-supl-regressao-espacial.git).

**Table 1.** Estimates of the Linear Regression and Spatial Autoregression models for the prevalence of adolescent mothers. Foz do Iguaçu (PR), Brazil, 2013-2019.

Explanatory variable	LRM			SAR		
	β	(β; 0.95) CI	p-value	β	(β; 0.95) CI	p-value
Brazilian Deprivation Index	4.13	(3.58; 4.68)	0.000	3.77	(3.13; 4.43)	0.000
Proportion of women household heads	0.09	(0.03; 0.14)	0.001	0.09	(0.03; 0.14)	0.000
Spatial autoregressive coefficient (ρ)	-	-	-	0.14	(0.00; 0.28)	0.055
**Measure**	**Value**		**p-valuev**	**Value**		**p-value**
Moran’s I	0.059		0.032	0.011		0.915
R^2^	0.43		-	0.44		-
AIC	2057.4		-	2055.8		-

LRM: Linear Regression Model; SAR: Spatial Autoregression.

## Data availability declaration

The data used in this study are public and can be accessed at the figshare repository (https://doi.org/10.6084/m9.figshare.27310467.v2).

The supplementary material for this article is available in the GitHub repository (https://github.com/LAES-USP/material-supl-regressao-espacial.git). The content includes a detailed description of the spatial modeling procedures, the commands in R (version 4.3), and the illustrations generated in GeoDa (version 22), as well as the files necessary for reproducing the analyses.

## Supplementary Material

Supplementary PDF
