# Supplementary material for: Linear regression in ecological studies involving space: methodology and an application example in public health
Source: Rev Bras Epidemiol. 2026 Apr 20;29:e260018. doi: 10.1590/1980-549720260018 (PMC13101397; doi:10.1590/1980-549720260018)
Supplement: Supplementary PDF [file 1980-5497-rbepid-29-e260018-sppl.pdf]

Material Suplementar

Exemplo de aplicação

Os dados utilizados neste exemplo são provenientes do estudo de Fernandes et al.(2024), que avaliaram a gravidez na adolescência no município de Foz do Iguaçu - PR, levando em conta sua distribuição espacial. O conjunto de dados está disponível em Fernandes et al. (2024b), contendo as seguintes variáveis:

- `RAZAO2`: Prevalência de mães adolescentes por setor censitário (multiplicada por 100).
- `IBP`: Índice Brasileiro de Privação.
- `PROPM2`: Proporção de mulheres responsáveis por domicílio (multiplicada por 100).

A seguir, apresentamos os comandos implementados na linguagem R, que oferece recursos completos, tanto para análise descritiva, quanto para análise de dados. Em complemento, exibimos os prints do GeoDa, um software gratuito voltado especificamente para análise espacial, que possibilita a visualização interativa e gráfica dos resultados e os diagnósticos do modelo.

A. Utilizando linguagem R

1. Definir opções para a saída do console

```r
options(scipen = 999)
```

2. Instalar e carregar bibliotecas

Antes de iniciar as análises, é importante assegurar que todos os pacotes necessários estejam instalados e que os dados do estudo estejam disponíveis no ambiente de trabalho. Para isso, utilizamos dois scripts auxiliares: um para instalar os pacotes e outro para realizar o download dos dados.

```r
# Executar script de instalação de pacotes
source("Scripts/00_instalar_pacotes.R")
```

```r
# Executa o script responsável pelo download dos dados utilizados na análise
source("Scripts/01_download_dados.R")
```

Carregar os pacotes utilizados:

```r
library(tidyverse)  # Manipulação de dados e gráficos
library(GGally)     # Extensão do ggplot2 para gráficos multivariados
library(sf)         # Análise de dados espaciais
library(RcmdrMisc)  # Para a matriz de correlação
library(tmap)       # Criação de mapas temáticos com dados espaciais
library(spdep)      # Dependência espacial e autocorrelação
library(spatialreg) # Modelos de regressão espacial
library(hnp)        # Para a análise de resíduos
```

3. Ler e visualizar o mapa com os dados

Ler o shapefile (mapa com dados):

```r
setores <- read_sf("dados_figshare/setores_foz.shp")
```

Plotar o mapa para visualização (R base):

```r
plot(st_geometry(setores))
```

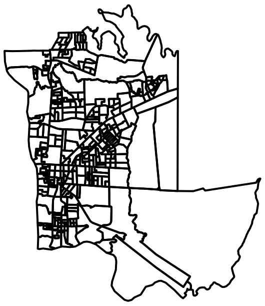

4. Visualizar estrutura dos dados e resumo estatístico

Visualizar a estrutura e as primeiras linhas do banco de dados:

```
glimpse(setores)
```

```
Rows: 322
Columns: 20
$ CD_GEOCODI <chr> "410830405000268", "410830405000269", "410830405000270", "4~
$ ADOLES     <dbl> 27, 17, 17, 8, 28, 2, 14, 3, 5, 11, 3, 12, 4, 15, 22, 16, 2~
$ TODAS      <dbl> 138, 78, 104, 93, 153, 56, 56, 72, 49, 143, 56, 55, 40, 78,~
$ IBP        <dbl> -0.7638875, -1.0722188, 0.2194413, -2.1010021, -0.8805667, ~
$ IBP2       <dbl> 3, 3, 4, 2, 3, 1, 4, 1, 1, 2, 1, 4, 1, 4, 4, 4, 3, 3, 2, 1,~
$ RAZAO      <dbl> 0.19565217, 0.21794872, 0.16346154, 0.08602151, 0.18300654,~
$ RAZAO2     <dbl> 19.565217, 21.794872, 16.346154, 8.602151, 18.300654, 3.571~
$ PROPM      <dbl> 0.3307888, 0.3741007, 0.2880000, 0.3194888, 0.5524194, 0.24~
$ PROPM2     <dbl> 33.07888, 37.41007, 28.80000, 31.94888, 55.24194, 24.77064,~
$ COORDX     <dbl> -54.55840, -54.50043, -54.51473, -54.53325, -54.52700, -54.~
$ COORDY     <dbl> -25.44582, -25.47619, -25.48553, -25.49387, -25.48877, -25.~
$ CLUSTER    <dbl> NA, 8, NA, NA, NA, 6, NA, 4, 6, 6, NA, NA, NA, NA, NA, NA, ~
$ RP_CLUSTER <dbl> NA, 1.5107234, NA, NA, NA, 0.6067010, NA, 0.5387019, 0.6067~
```

```
$ V0          <dbl> 8, 5, 3, 7, 8, 2, 1, 3, 2, 4, 1, 6, 2, 3, 6, 4, 7, 3, 3, 2,~
$ V1          <dbl> 9, 10, 10, 1, 11, 0, 7, 0, 1, 5, 2, 4, 2, 6, 6, 9, 10, 4, 1~
$ V2          <dbl> 5, 1, 4, 0, 7, 0, 4, 0, 2, 2, 0, 1, 0, 5, 8, 2, 3, 3, 0, 0,~
$ V3          <dbl> 5, 1, 0, 0, 2, 0, 2, 0, 0, 0, 0, 1, 0, 1, 2, 1, 2, 0, 1, 0,~
$ resp_total <dbl> 393, 278, 250, 313, 248, 218, 100, 246, 202, 431, 212, 132,~
$ resp_mulhe <dbl> 130, 104, 72, 100, 137, 54, 33, 78, 83, 221, 76, 49, 60, 64~
$ geometry   <POLYGON [°]> POLYGON ((-54.55675 -25.445..., POLYGON ((-54.50125~
```

Algumas estatísticas descritivas para as variáveis do banco de dados:

```r
summary(setores)
```

```
  CD_GEOCODI             ADOLES           TODAS              IBP         
 Length:322         Min.   :  0.00   Min.   :  3.00   Min.   :-3.0448  
 Class :character   1st Qu.:  5.00   1st Qu.: 52.00   1st Qu.:-2.1495  
 Mode  :character   Median :  9.00   Median : 74.00   Median :-1.3391  
                    Mean   : 12.78   Mean   : 91.58   Mean   :-1.2376  
                    3rd Qu.: 16.00   3rd Qu.:109.00   3rd Qu.:-0.6557  
                    Max.   :123.00   Max.   :506.00   Max.   : 2.9928  
                                                                        
      IBP2           RAZAO             RAZAO2           PROPM       
 Min.   :1.000   Min.   :0.00000   Min.   : 0.000   Min.   :0.0000  
 1st Qu.:2.000   1st Qu.:0.08355   1st Qu.: 8.355   1st Qu.:0.3186  
 Median :3.000   Median :0.12862   Median :12.862   Median :0.3755  
 Mean   :2.484   Mean   :0.13905   Mean   :13.905   Mean   :0.3917  
 3rd Qu.:3.000   3rd Qu.:0.19145   3rd Qu.:19.145   3rd Qu.:0.4415  
 Max.   :5.000   Max.   :0.42857   Max.   :42.857   Max.   :0.7561  
                                                                     
     PROPM2          COORDX           COORDY          CLUSTER      
 Min.   : 0.00   Min.   :-54.60   Min.   :-25.61   Min.   : 1.000  
 1st Qu.:31.86   1st Qu.:-54.58   1st Qu.:-25.54   1st Qu.: 4.000  
 Median :37.55   Median :-54.56   Median :-25.52   Median : 6.000  
 Mean   :39.17   Mean   :-54.56   Mean   :-25.52   Mean   : 5.516  
 3rd Qu.:44.15   3rd Qu.:-54.54   3rd Qu.:-25.49   3rd Qu.: 7.000  
 Max.   :75.61   Max.   :-54.47   Max.   :-25.44   Max.   :11.000  
                                                   NA's   :194     
   RP_CLUSTER           V0               V1               V2        
 Min.   :0.1243   Min.   : 0.000   Min.   : 0.000   Min.   : 0.000  
 1st Qu.:0.5387   1st Qu.: 2.000   1st Qu.: 2.000   1st Qu.: 0.000  
 Median :0.6067   Median : 3.000   Median : 4.000   Median : 2.000  
 Mean   :0.9057   Mean   : 4.391   Mean   : 5.025   Mean   : 2.683  
 3rd Qu.:1.4879   3rd Qu.: 6.000   3rd Qu.: 7.000   3rd Qu.: 4.000  
```

```
 Max.   :2.0418   Max.   :29.000   Max.   :48.000   Max.   :33.000
 NA's   :194
       V3             resp_total      resp_mulhe              geometry
 Min.   : 0.0000   Min.   :  5.0   Min.   :  0.00   POLYGON      :322
 1st Qu.: 0.0000   1st Qu.:183.2   1st Qu.: 67.00   epsg:4674    :  0
 Median : 0.0000   Median :240.0   Median : 88.00   +proj=long...:  0
 Mean   : 0.6739   Mean   :244.7   Mean   : 97.13
 3rd Qu.: 1.0000   3rd Qu.:297.2   3rd Qu.:118.00
 Max.   :13.0000   Max.   :699.0   Max.   :431.00
```

5. Criar dataframe regular

A criação de dataframe regular com apenas as variáveis de interesse, sem espacialização, facilita a realização de algumas operações em R.

```
setores_df <- setores |>
        st_drop_geometry() |>               # Remover coluna de geometria
        as.data.frame() |>                  # Converter para um dataframe regular
        dplyr::select(CD_GEOCODI, RAZAO2, IBP, PROPM2)  # Selecionar as variáveis de
↪   interesse
```

6. Análise Exploratória Não Espacial

6.1. Descrever cada variável individualmente

6.1.1. Medidas de tendência central e variabilidade

Calcular medidas de tendência central e variabilidade para cada variável e reorganizar os dados para formato tabular:

```
medidas_tendencia_central <- setores_df  |>
        summarise(
                Média_RAZAO2 = mean(RAZAO2, na.rm = TRUE),      # Calcula a média de
↪   RAZAO2, ignorando valores NA
                Mediana_RAZAO2 = median(RAZAO2, na.rm = TRUE),  # Calcula a mediana
                 ↪  de RAZAO2, ignorando valores NA
                DP_RAZAO2 = sd(RAZAO2, na.rm = TRUE),           # Calcula o desvio
                 ↪  padrão de RAZAO2, ignorando valores NA
                Média_IBP = mean(IBP, na.rm = TRUE),            # Calcula a média de
↪   IBP, ignorando valores NA
                Mediana_IBP = median(IBP, na.rm = TRUE),        # Calcula a mediana
                 ↪  de IBP, ignorando valores NA
```

Table 1: Medidas de tendência central e variabilidade para variáveis selecionadas

Variável	Média	Mediana	DP
RAZAO2	13.904993	12.861869	7.748910
IBP	-1.237594	-1.339051	1.176122
PROPM2	39.169561	37.550000	11.962748

```
                DP_IBP = sd(IBP, na.rm = TRUE),                 # Calcula o desvio
                 ↪  padrão de IBP, ignorando valores NA
                Média_PROPM2 = mean(PROPM2, na.rm = TRUE),      # Calcula a média de
 ↪  PROPM2, ignorando valores NA
                Mediana_PROPM2 = median(PROPM2, na.rm = TRUE),  # Calcula a mediana
                 ↪  de PROPM2, ignorando valores NA
                DP_PROPM2 = sd(PROPM2, na.rm = TRUE)            # Calcula o desvio
                 ↪  padrão de PROPM2, ignorando valores NA
        ) |>
        pivot_longer(
                cols = everything(),                 # Seleciona todas as colunas
                 ↪  para reorganizar
                names_to = c("Medida", "Variável"),  # Divide os nomes das colunas em
                 ↪  "Medida" e "Variável"
                names_sep = "_",                     # Usa "_" como separador para
                 ↪  dividir os nomes das colunas
                values_to = "Valor"                  # Nome da nova coluna que
                 ↪  armazenará os valores
        ) %>%
        pivot_wider(
                names_from = Medida,                 # Transforma os valores da
                 ↪  coluna "Medida" em cabeçalhos de coluna
                values_from = Valor                  # Os valores associados são
                 ↪  extraídos da coluna "Valor"
        )


# Criar tabela
knitr::kable(
        medidas_tendencia_central,                                          #
 ↪  Dataframe que contém os dados reorganizados
        format = (if (knitr::is_latex_output()) "latex" else "html"),
         ↪  # Especifica o formato da tabela
        caption = "Medidas de tendência central e variabilidade para variáveis
         ↪  selecionadas" # Adiciona uma legenda à tabela
)
```

6.1.2. Gráficos: Histograma, Box-plot e Dotplot

Variável resposta: Prevalência de mães adolescentes

Histograma

```
ggplot(setores_df, aes(x = RAZA02)) +                                     # Define a
↪ variável 'RAZA02' no eixo x
        geom_histogram(fill = "lightblue", color = "black", bins = 20) + # Cria o
        ↪ histograma com cor de preenchimento e borda, bins = número de barras
        labs(
                x = "Prevalência de mães adolescentes",                   # Título do
                ↪ eixo x
                y = "Número de setores") +                                # Título do
                ↪ eixo y
        theme_minimal()                                                   # Aplica o
        ↪ tema minimal gráfico
```

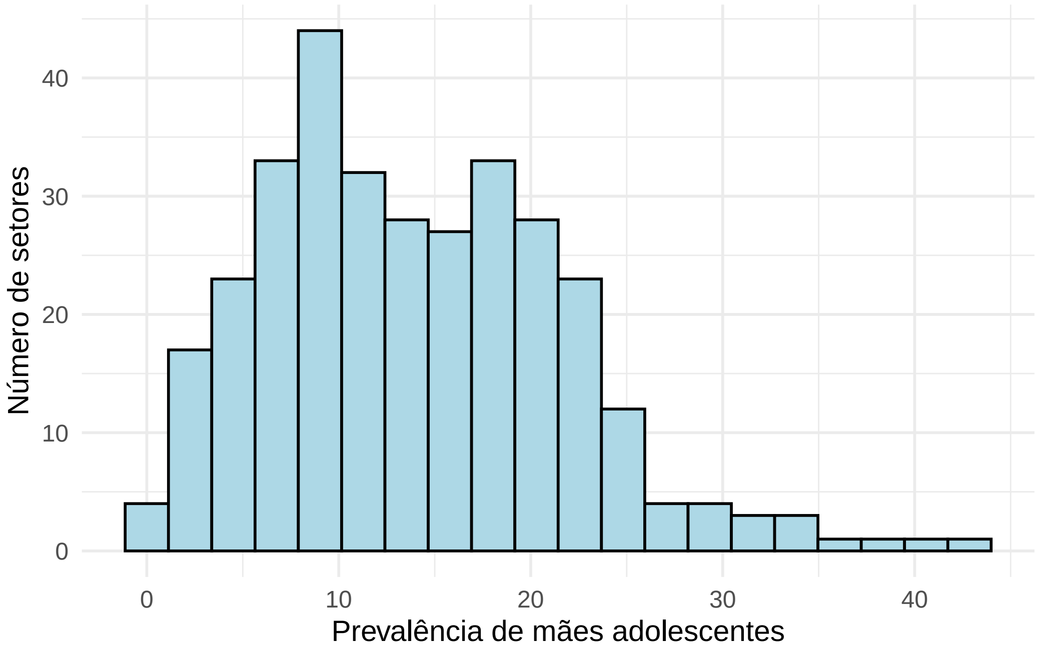

Box-plot

```
ggplot(setores_df, aes(y = RAZA02)) +                   # Cria o gráfico com 'RAZA02'
↪ no eixo y
        geom_boxplot() +                                      # Adiciona o box-plot
        labs(y = "Prevalência de mães adolescentes") +        # Define o título e o
        ↪ rótulo do eixo y
        theme_minimal() +                                     # Aplica o tema minimal
        ↪ gráfico
        theme(axis.text.x = element_blank())                  # Remove os rótulos do
        ↪ eixo x
```

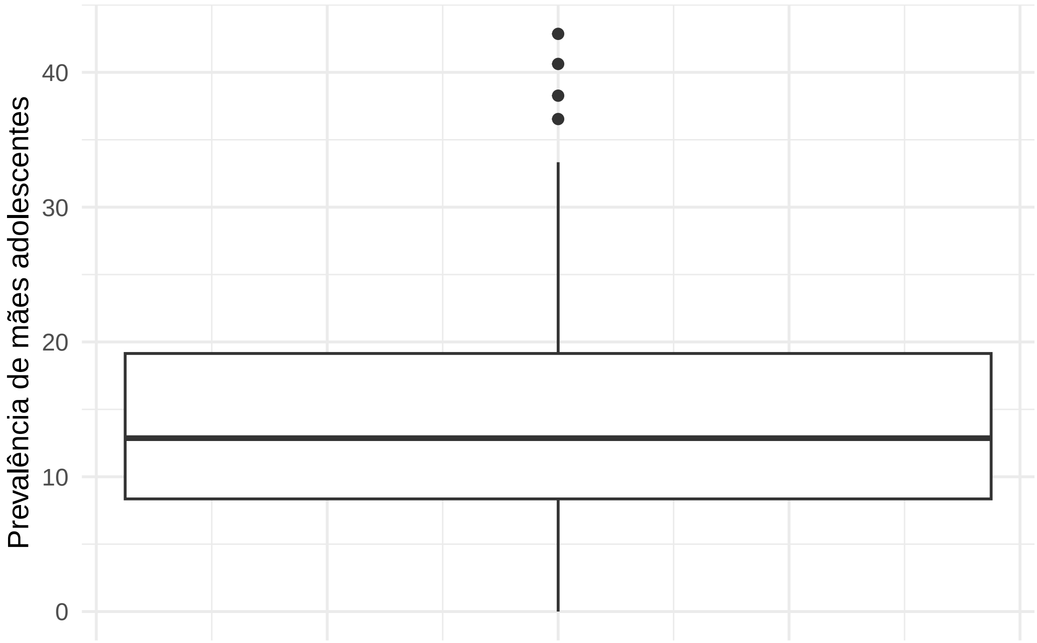

Dotplot

```
# Gera uma sequência de números de 1 até o número de linhas do dataframe,
↪  representando a ordem das observações que serão plotadas no eixo Y de cada um dos
↪  dotplot
ordem_observacoes <- 1:nrow(setores_df)

ggplot(setores_df,                                      # Cria o gráfico com 'RAZA02'
↪  no eixo x e 'ordem_observacoes' no eixo y
       aes(y = ordem_observacoes, x = RAZA02)) +
        geom_point() +                                  # Adiciona os pontos ao
         ↪  gráfico
        theme_minimal() +                               # Aplica o tema minimal
        labs( x = "Prevalência de mães adolescentes",
              y = "Ordem das observações")              # Define os rótulos dos eixos
```

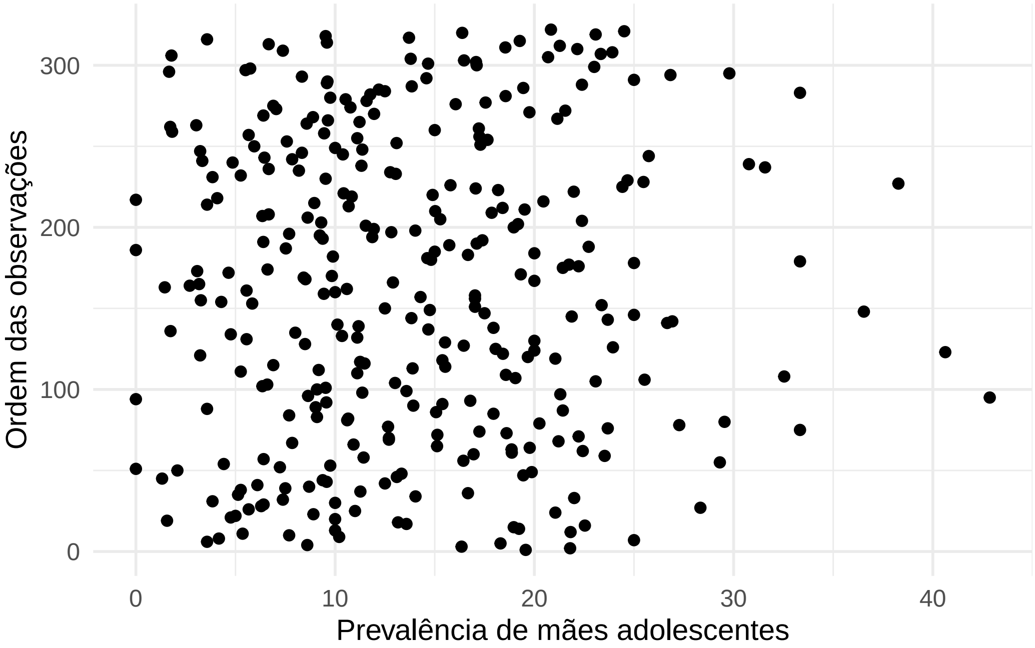

O box-plot e o dotplot apontam a existência de *outliers* na variável resposta. Deve-se investigar cada um e avaliar sua influência nas estimativas do modelo.

Covariável: Índice Brasileiro de Privação

Histograma

```
ggplot(setores_df, aes(x = IBP)) +                                    # Define a
↪  variável 'IBP' no eixo x
        geom_histogram(fill = "lightblue", color = "black", bins = 20) + # Cria o
        ↪  histograma com cor de preenchimento e borda, bins = número de barras
        labs(
                x = "Índice Brasileiro de Privação",                  # Título do
                ↪  eixo x
                y = "Número de setores"                               # Título do
                ↪  eixo y
        ) +
        theme_minimal() +                                             # Aplica o
        ↪  tema minimal gráfico
        theme(axis.text.x = element_blank())                          # Remove os
        ↪  rótulos do eixo x
```

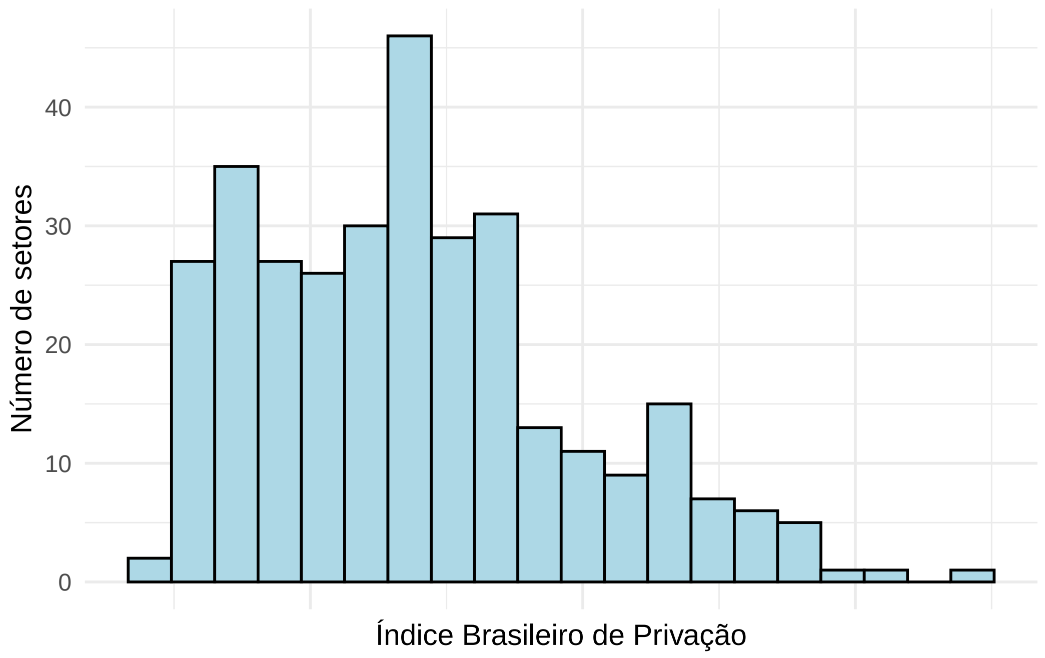

Box-plot

```
ggplot(setores_df, aes(y = IBP)) +                        # Cria o gráfico com
↪  'IBP' no eixo y
        geom_boxplot() +                                  # Adiciona o box-plot
        labs(y = "Índice Brasileiro de Privação") +       # Define o rótulo do eixo
         ↪  y
        theme_minimal() +                                 # Aplica o tema minimal
         ↪  gráfico
        theme(axis.text.x = element_blank())              # Remove os rótulos do
         ↪  eixo x
```

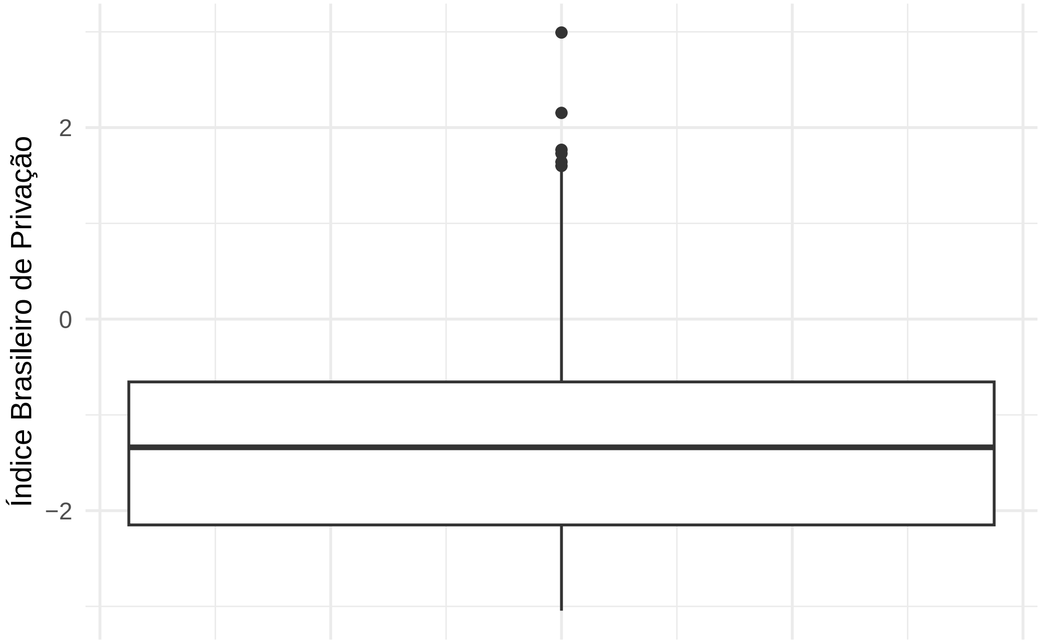

Dotplot

```
ggplot(setores_df,                                          # Cria o gráfico com 'IBP' no
 ↪  eixo x e 'ordem_observacoes' no eixo y
       aes(y = ordem_observacoes, x = IBP)) +
        geom_point() +                                      # Adiciona os pontos ao
         ↪  gráfico
        theme_minimal() +                                   # Aplica o tema minimal
        labs(x = "Índice Brasileiro de Privação",
             y = "Ordem das observações")                   # Define os rótulos dos eixos
              ↪
```

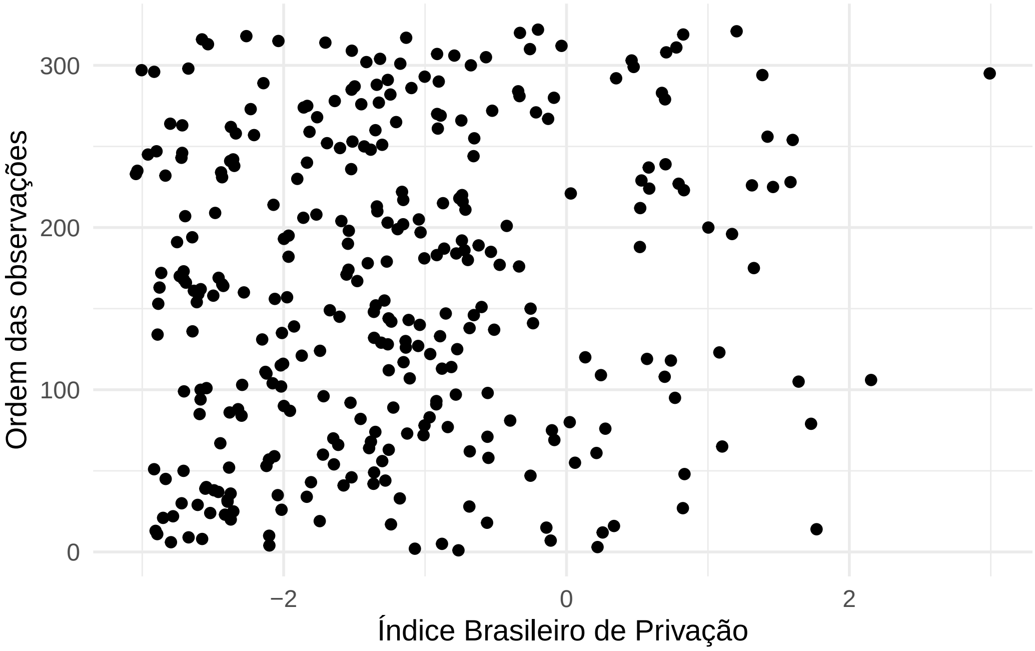

O box-plot e o dotplot apontam a existência de *outliers* na variável resposta. Deve-se investigar cada um e avaliar sua influência nas estimativas do modelo.

Covariável: Proporção de mulheres responsáveis pelo domicílio

Histograma

```
ggplot(setores_df, aes(x = PROPM2)) +                                  # Define a
↪  variável PROPM2 no eixo x
        geom_histogram(fill = "lightblue", color = "black", bins = 20) + # Cria o
         ↪  histograma com cor de preenchimento e borda, bins = número de barras
        labs(
                x = "Proporção de mulheres responsáveis por domicílio",  # Título do
                 ↪  eixo x
                y = "Número de setores"                                  # Título do
                 ↪  eixo y
        ) +
        theme_minimal()                                                  # Aplica o
         ↪  tema minimal
```

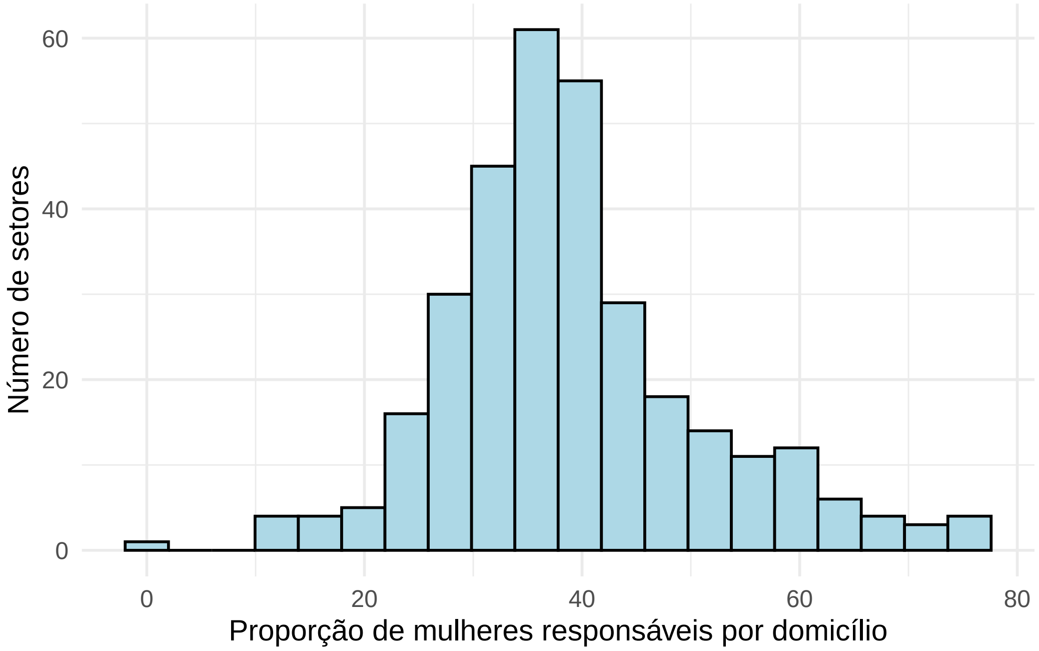

Box-plot

```
ggplot(setores_df, aes(y = PROPM2)) +                                # Cria o
↪  gráfico com 'PROPM2' no eixo y
        geom_boxplot() +                                             # Adiciona o
        ↪  box-plot
        labs(y = "Proporção de mulheres responsáveis por domicílio") + # Define o
        ↪  rótulo do eixo y
        theme_minimal() +                                            # Aplica o
        ↪  tema minimal gráfico
        theme(axis.text.x = element_blank())                         # Remove os
        ↪  rótulos do eixo x
```

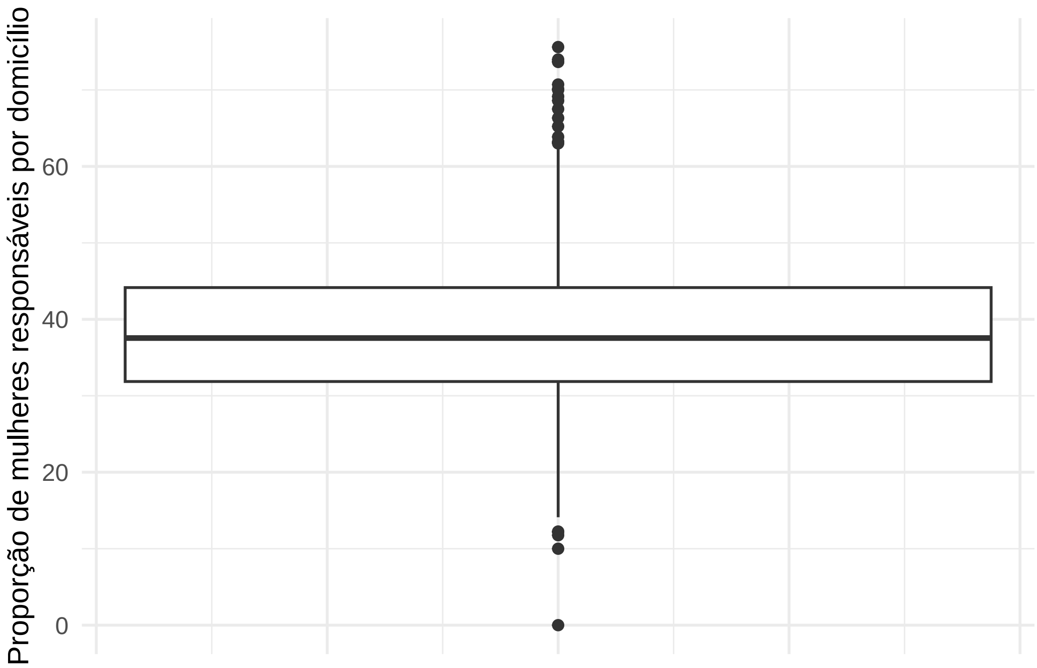

Dotplot

```
ggplot(setores_df,                                         # Cria o gráfico com
 ↪  'PROPM2' no eixo x e 'ordem_observacoes' no eixo y
       aes(y = ordem_observacoes, x = PROPM2)) +
        geom_point() +                                     # Adiciona os pontos ao
         ↪  gráfico
        theme_minimal() +                                  # Aplica o tema minimal
        labs(
                x = "Proporção de mulheres responsáveis por domicílio",
                y = "Ordem das observações")               # Define os rótulos dos
                 ↪  eixos
```

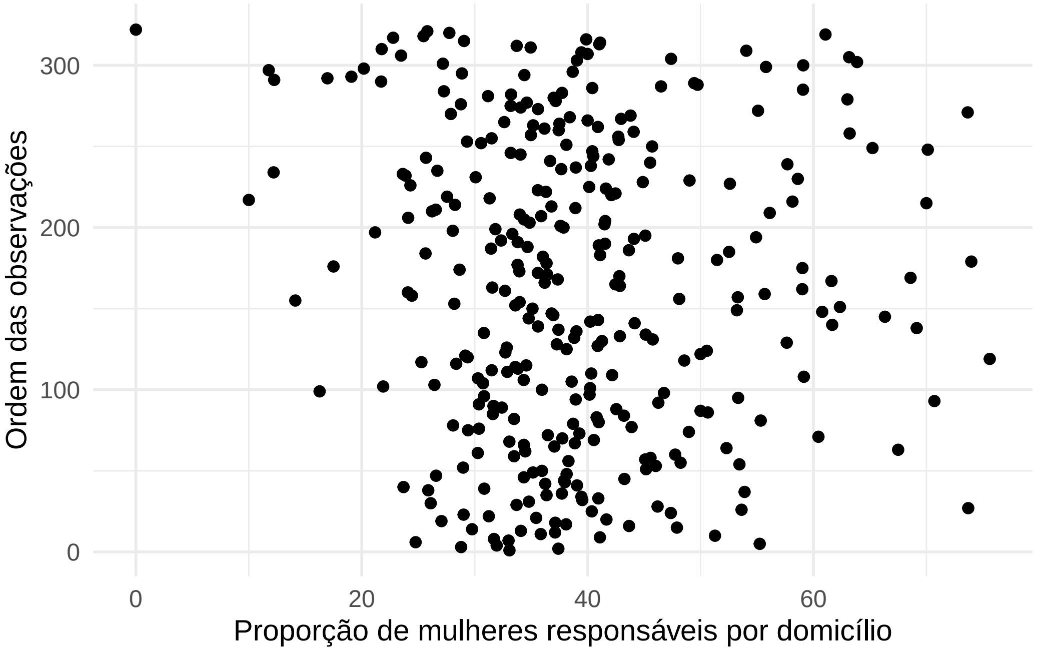

O box-plot e o dotplot apontam a existência de *outliers* na variável resposta. Deve-se investigar cada um e avaliar sua influência nas estimativas do modelo.

6.2. Descrever as associações da resposta com cada covariável, bem como das covariáveis entre si

Como todas as covariáveis são quantitativas, serão utilizados apenas diagramas de dispersão e coeficientes de correlação.

6.2.1 Diagramas de dispersão

Nesses gráficos, a variável que acredita-se depender da outra deve sempre ser alocada no eixo y.

Variável resposta (RAZAO2) x PROPM2

```
ggplot(data = setores_df, mapping = aes(x = PROPM2, y = RAZAO2)) +
        geom_point(color = "black") +
         ↪  # Pontos para cada observação
        geom_smooth(aes(color = "Linha suavizada"), se = FALSE) +
         ↪  # Linha de suavização (loess) sem banda de confiança
        geom_smooth(aes(color = "Reta de regressão e bandas de confiança"), method =
         ↪  lm) +             # Regressão linear com banda de confiança
```

```
scale_color_manual(
    # Define cores manualmente para cada linha representada na legenda
    values = c(
        "Linha suavizada" = "orange",
        "Reta de regressão e bandas de confiança" = "blue"
    )
) +
labs(
    x = "Proporção de mulheres responsáveis pelo domicílio",
    # Eixo x com unidade
    y = "Prevalência de mães adolescentes"
    # Eixo y com unidade
) +
theme_minimal() +                                                # Aplica
    tema minimal
theme(legend.position = "bottom",                                # Posiciona
    a legenda abaixo do gráfico
      legend.title = element_blank())                            # Remove o
      título da legenda
```

```
`geom_smooth()` using method = 'loess' and formula = 'y ~ x'
`geom_smooth()` using formula = 'y ~ x'
```

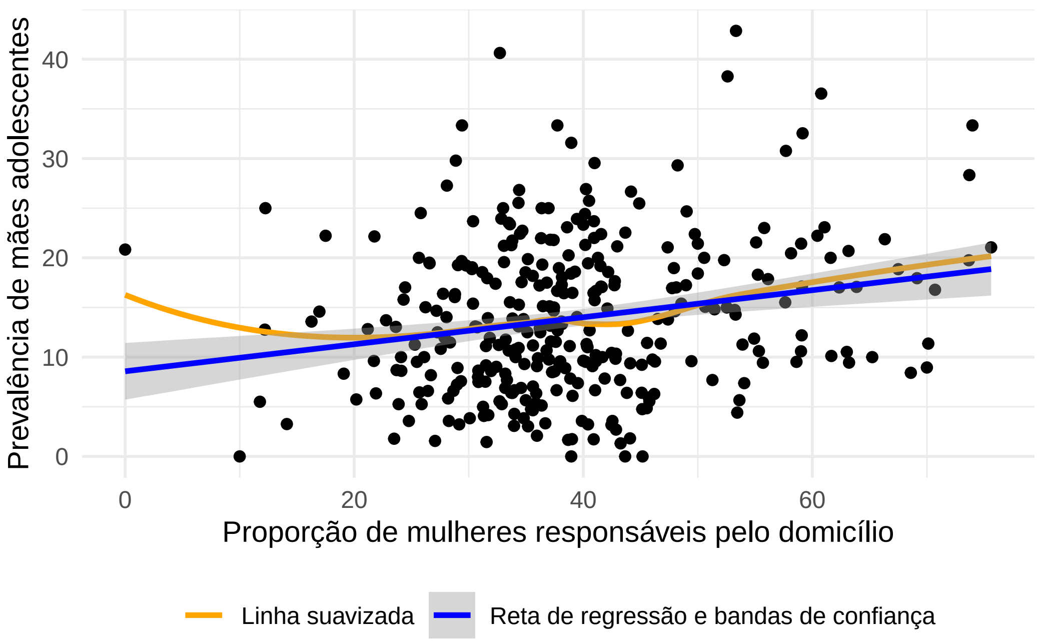

O diagrama sugere uma relação linear entre a variável resposta (RAZAO2) e a PROPM2: a

prevalência de mães adolescentes aumenta à medida que aumenta a proporção de mulheres responsáveis pelo domicílio.

Variável resposta (RAZAO2) x IBP

```
ggplot(data = setores_df, mapping = aes(x = IBP, y = RAZAO2)) +
        geom_point(color = "black") +
         ↪  # Pontos para cada observação
        geom_smooth(aes(color = "Linha suavizada"), se = FALSE) +
         ↪  # Linha de suavização (loess) sem banda de confiança
        geom_smooth(aes(color = "Reta de regressão e bandas de confiança"), method =
         ↪  lm) +            # Regressão linear com banda de confiança
        scale_color_manual(
         ↪  # Define cores manualmente para cada linha representada na legenda
                values = c(
                        "Linha suavizada" = "orange",
                        "Reta de regressão e bandas de confiança" = "blue"
                )
        ) +
        labs(
                x = "Índice Brasileiro de Privação",                # Eixo x com
                 ↪  unidade
                y = "Prevalência de mães adolescentes"              # Eixo y com
                 ↪  unidade
        ) +
        theme_minimal() +                                                  # Aplica
         ↪  tema minimal
        theme(legend.position = "bottom",                                  # Posiciona
         ↪  a legenda no topo do gráfico
              legend.title = element_blank())                              # Remove o
               ↪  título da legenda
```

```
`geom_smooth()` using method = 'loess' and formula = 'y ~ x'
`geom_smooth()` using formula = 'y ~ x'
```

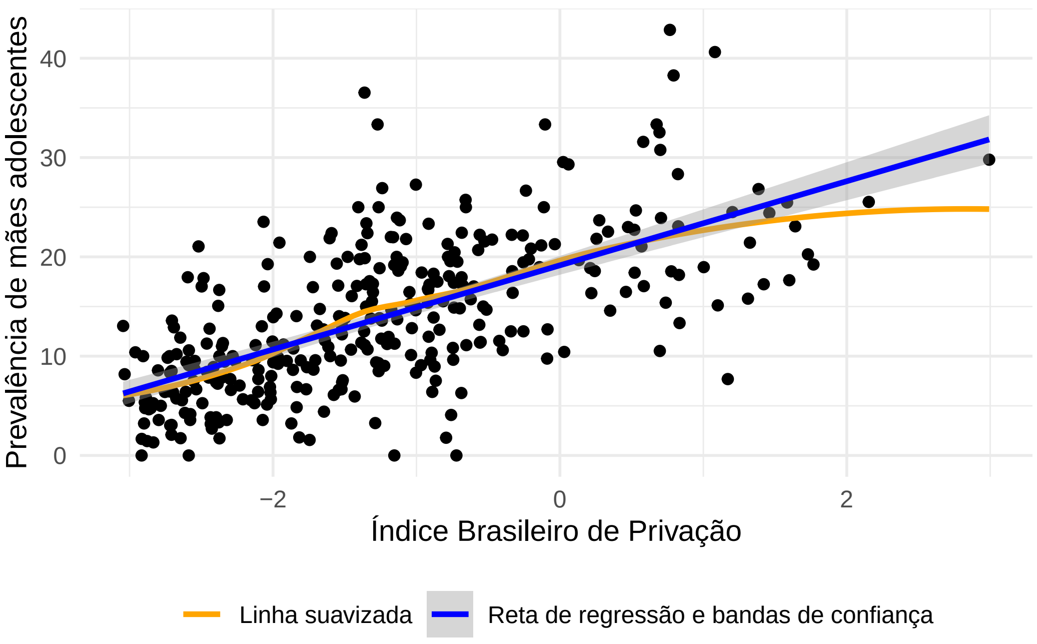

O diagrama sugere uma relação linear entre a variável resposta (RAZAO2) e o IBP: a prevalência de mães adolescentes aumenta à medida que aumenta o Índice Brasileiro de Privação.

PROPM2 x IBP

```
ggplot(data = setores_df, mapping = aes(x = IBP, y = PROPM2)) +
        geom_point(color = "black") +
         ↪  # Pontos para cada observação
        geom_smooth(aes(color = "Linha suavizada"), se = FALSE) +
         ↪  # Linha de suavização (loess) sem banda de confiança
        geom_smooth(aes(color = "Reta de regressão e bandas de confiança"), method =
         ↪  lm) +             # Regressão linear com banda de confiança
        scale_color_manual(
         ↪  # Define cores manualmente para cada linha representada na legenda
                values = c(
                        "Linha suavizada" = "orange",
                        "Reta de regressão e bandas de confiança" = "blue"
                )
        ) +
        labs(
                x = "Índice Brasileiro de Privação",
                 ↪  # Eixo x com unidade
                y = "Proporção de mulheres responsáveis pelo domicílio"
                 ↪  # Eixo y com unidade
        ) +
```

```
    theme_minimal() +                                              # Aplica
     ↪  tema minimal
    theme(legend.position = "bottom",                              # Posiciona
     ↪  a legenda abaixo do gráfico
          legend.title = element_blank())                          # Remove o
           ↪  título da legenda
```

```
`geom_smooth()` using method = 'loess' and formula = 'y ~ x'
`geom_smooth()` using formula = 'y ~ x'
```

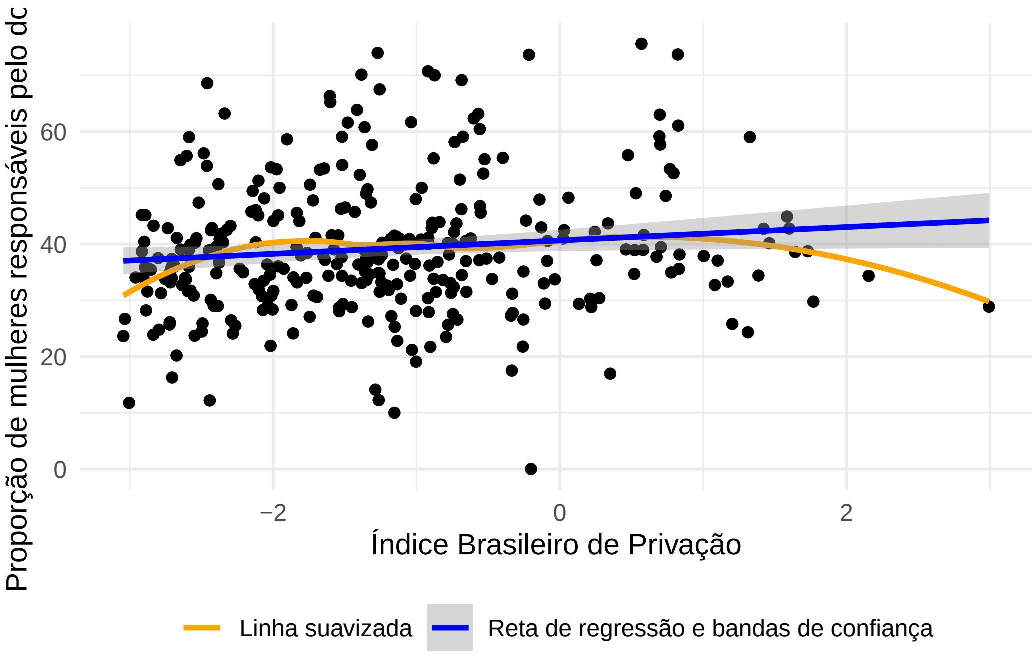

O diagrama sugere uma relação linear entre as covariáveis PROPM2 e IBP: A proporção de mulheres responsáveis pelo domicílio aumenta levemente à medida que aumenta o Índice Brasileiro de Privação.

6.2.2 Matriz de correlação de Pearson

```
rcorr.adjust(setores_df[,c("RAZAO2", "IBP", "PROPM2" )], type = "pearson", use =
 ↪  "complete")
```

```
Pearson correlations:
```

```
       RAZAO2    IBP PROPM2
RAZAO2 1.0000 0.6429 0.2101
IBP    0.6429 1.0000 0.1171
PROPM2 0.2101 0.1171 1.0000

 Number of observations: 322

 Pairwise two-sided p-values:
       RAZAO2 IBP    PROPM2
RAZAO2        <.0001 0.0001
IBP    <.0001        0.0357
PROPM2 0.0001 0.0357

 Adjusted p-values (Holm's method)
       RAZAO2 IBP    PROPM2
RAZAO2        <.0001 0.0003
IBP    <.0001        0.0357
PROPM2 0.0003 0.0357
```

Uma vez que as associações observadas nos diagramas de dispersão são lineares, os coeficientes de correlação podem ser interpretados da forma usual. Os coeficientes sugerem uma associação linear entre a variável resposta (RAZAO2) e as covariáveis IBP (moderada) e PROPM2 (fraca).

6.2.3 Matriz de dispersão e correlação de Pearson

Ao criar a matriz, deve-se especificar as variáveis na ordem correta, a fim de que a variável que acredita-se depender da outra esteja alocada no eixo y.

```
ggpairs(
        setores_df[, c("IBP", "PROPM2", "RAZAO2")],   # Selecione a variável resposta
 ↪  e as covariáveis de interesse
        upper = list(continuous = "cor"),             # Exibe a correlação de Pearson
         ↪  no painel superior
        lower = list(continuous = "smooth"),          # Exibe gráficos de dispersão
         ↪  com linha de suavização no painel inferior
        diag = list(continuous = "densityDiag"),      # Exibe histogramas de
         ↪  densidade no painel diagonal
        title = "Avaliação gráfica das relações entre variável resposta e
         ↪  covariáveis"  # Título do gráfico
)
```

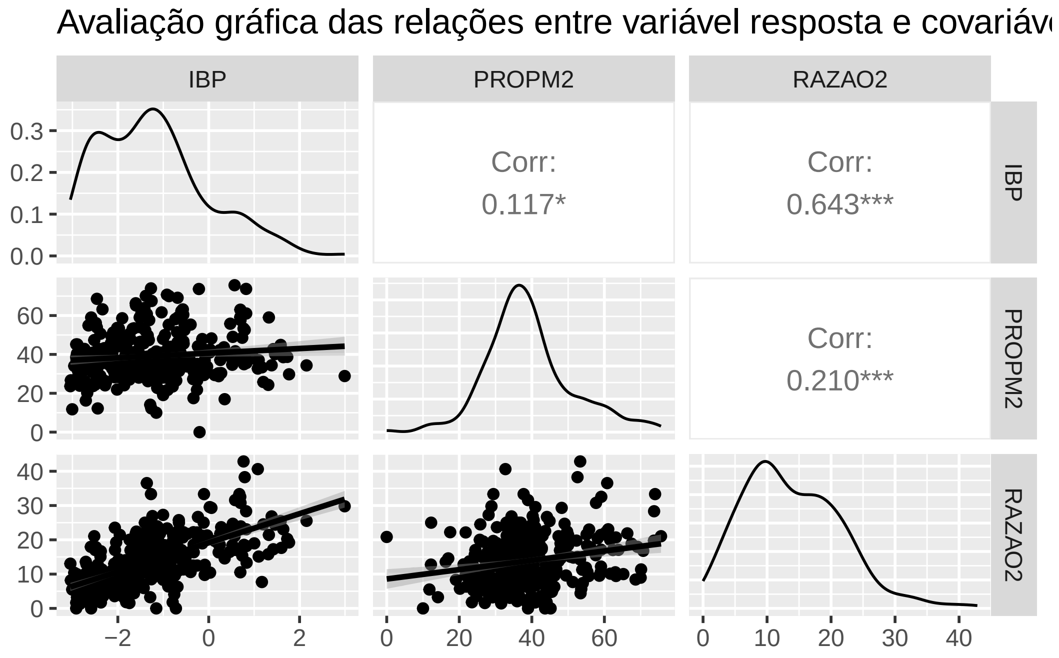

6.3. Avaliar a normalidade da variável resposta

6.3.1 Qq-plot

```
ggplot(setores_df, aes(sample = RAZAO2)) +
        stat_qq() +                                             # Plota os pontos dos
         ↪  quantis observados x teóricos (distribuição normal)
        stat_qq_line() +                                        # Adiciona a linha de
         ↪  referência para uma distribuição normal
        labs(y = "Prevalência de mães adolescentes",            # Título do eixo Y
             x = "Quantis da distribuição normal padrão") +     # Título do eixo X
        theme_minimal()                                         # Aplica tema minimal
```

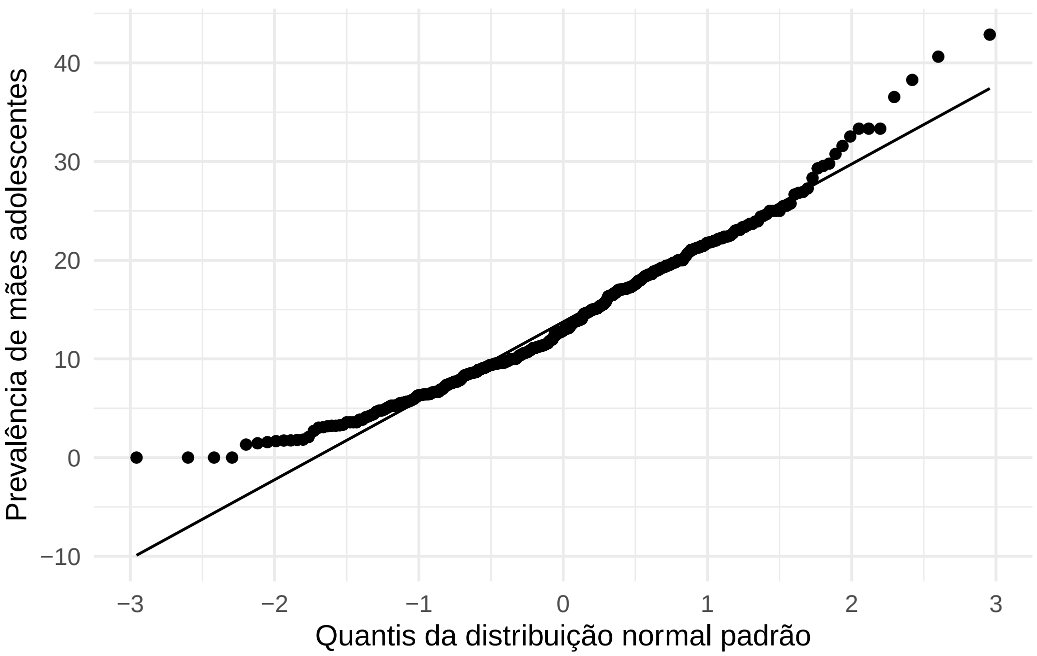

Há desvio da normalidade nas extremidades (valores muito grandes ou muito pequenos de RAZAO2). Após o ajuste do modelo, deve-se verificar se os resíduos ainda apresentam desvio de normalidade.

6.3.2 Teste de normalidade de Kolmogorov-Smirnov

```r
ks.test(setores_df$RAZAO2, "pnorm", mean(setores_df$RAZAO2), sd(setores_df$RAZAO2))
```

```
Warning in ks.test.default(setores_df$RAZAO2, "pnorm", mean(setores_df$RAZAO2),
: não devem existir empates no teste de Kolmogorov-Smirnov de apenas uma
amostra


	Asymptotic one-sample Kolmogorov-Smirnov test

data:  setores_df$RAZAO2
D = 0.077606, p-value = 0.04136
alternative hypothesis: two-sided
```

O p-valor é pouco menor do que 0,05, indicando desvio da normalidade. Após o ajuste do modelo, deve-se verificar se os resíduos ainda apresentam desvio de normalidade. Se isso ocorrer, o modelo não será adequado e será necessário buscar outros modelos mais adequados.

7. Análise Exploratória Espacial

7.1. Mapas temáticos

Visualizar a distribuição espacial das variáveis de interesse:

```
# Adicionar interação
tmap_mode("plot")
```

```
i tmap mode set to "plot".
```

```
# Criar mapa para a variável RAZAO2)

map_razao2 <- tm_shape(setores) +                                    # Define o
↪ conjunto de dados geográficos
        tm_polygons(
                fill = "RAZAO2",                                     # Define a
                ↪ variável para o preenchimento dos polígonos
                fill.scale = tm_scale(values = "brewer.blues"),      # Aplica a
                ↪ escala de cores
                fill.legend = tm_legend(title = "RAZAO2")            # Define o
                ↪ título da legenda
        ) +
        tm_title("Variável resposta") +                        # Adiciona um
        ↪ título ao mapa
        tm_layout(legend.outside = TRUE)                       # Mantém a
        ↪ legenda fora do mapa


# Criar mapa para a variável IBP
map_ibp <- tm_shape(setores) +
        tm_polygons(
                fill = "IBP",                                    # Define a
                ↪ variável para o preenchimento dos polígonos
                fill.scale = tm_scale(values = "brewer.greens"), # Aplica a
                ↪ escala de cores
                fill.legend = tm_legend(title = "IBP"),          # Define o
                ↪ título da legenda
                col = "black"                                    # Define a cor
                ↪ das bordas dos polígonos
        ) +
        tm_title("Covariáveis") +                            # Adiciona um título
        ↪ ao mapa
        tm_layout(legend.outside = TRUE)                     # Mantém a legenda
        ↪ fora do mapa
```

```r
# Criar mapa para a variável PROPM2
map_propm2 <- tm_shape(setores) +
        tm_polygons(
                fill = "PROPM2",                                # Define a
                 ↪  variável para o preenchimento dos polígonos
                fill.scale = tm_scale(values = "brewer.reds"),  # Aplica a
                 ↪  escala de cores
                fill.legend = tm_legend(title = "PROPM2"),      # Define o
                 ↪  título da legenda
                col = "black"                                   # Define a cor
                 ↪  das bordas dos polígonos
        ) +
        tm_title("Covariáveis") +                         # Adiciona um título
         ↪  ao mapa
        tm_layout(legend.outside = TRUE)                  # Mantém a legenda
         ↪  fora do mapa

# Mostrar mapa para a variável desfecho RAZAO2
map_razao2
```

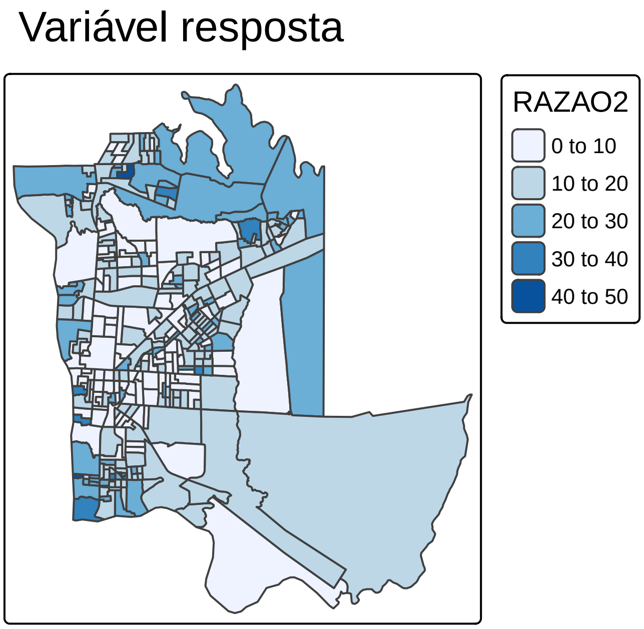


```r
# Mostrar os mapas das covariáveis lado a lado
tmap_arrange(map_ibp, map_propm2)
```

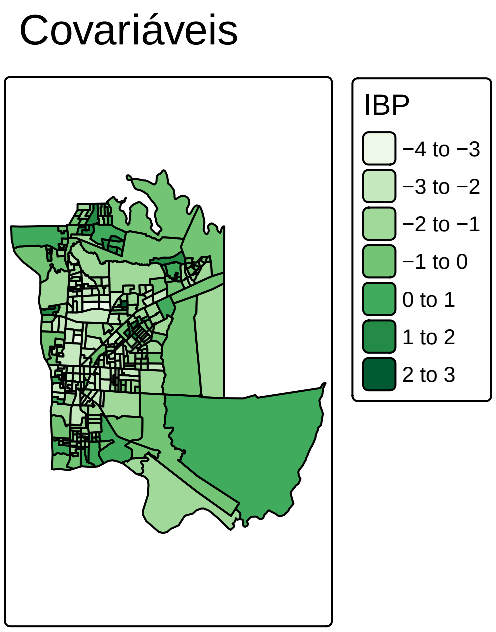

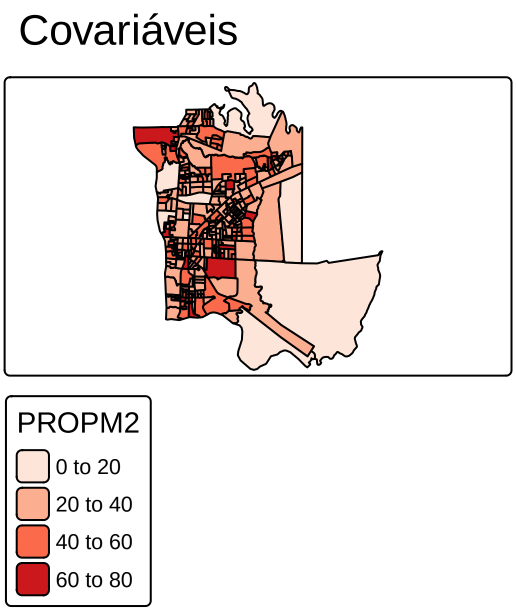

7.2. Dependência espacial da variável resposta

7.2.1. Matriz de vizinhança espacial

Criar a matriz de vizinhança do tipo "queen" (considera vizinhos que compartilham borda ou vértice):

```
matriz_viz_queen <- poly2nb(setores, queen = TRUE) # Cria matriz de vizinhança usando
 ↪  critério "queen"
print(matriz_viz_queen)                            # Exibe dados da matriz de
 ↪  vizinhança
```

```
Neighbour list object:
Number of regions: 322
Number of nonzero links: 1952
Percentage nonzero weights: 1.882643
Average number of links: 6.062112
```

Plotar mapa de vizinhos com base na contiguidade "queen":

```r
# Plotar a geometria dos setores espaciais com bordas em cinza
plot(st_geometry(setores), border = "gray")
# Adicionar as conexões de vizinhança ao mapa anterior (`matriz_viz_queen`: vizinhos
↪  espaciais, `st_geometry(setores)`: coordenadas dos setores)
plot.nb(matriz_viz_queen, st_geometry(setores), add = TRUE)
```

```
Warning in st_point_on_surface.sfc(coords): st_point_on_surface may not give
correct results for longitude/latitude data
```

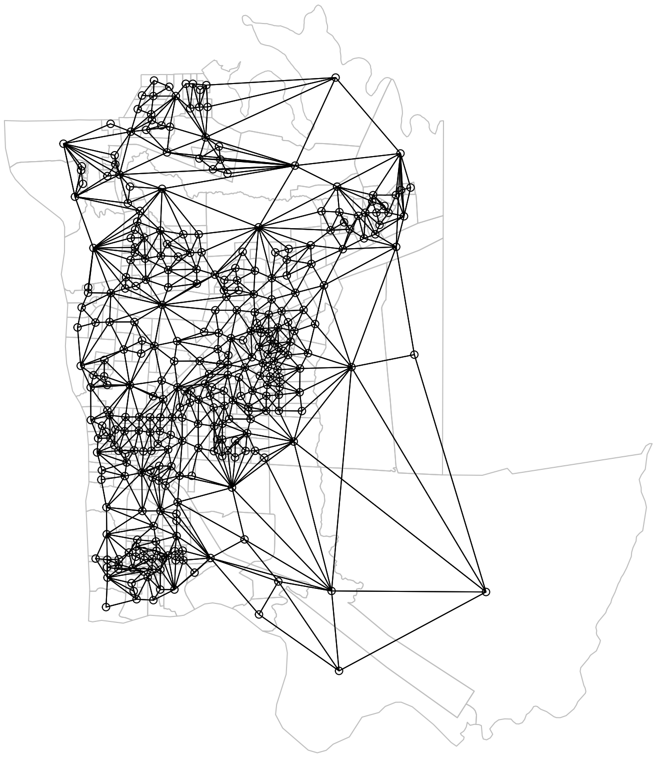

Criar histograma mostrando a distribuição do número de vizinhos por setor censitário:

```r
# Contar o número de vizinhos por setor (função card() do pacote spdep)
vizinhos <- data.frame(id_setores = 1:nrow(setores),       # Identificação dos
↪  setores
```

```
                       n_vizinhos = card(matriz_viz_queen) # Número de vizinhos por
                        ↪  setor
)

# Criar o histograma
ggplot(vizinhos, aes(x = n_vizinhos)) +
        geom_histogram(binwidth = 1, fill = "lightblue", color = "black") +         #
         ↪  Cria o histograma com cor de preenchimento e borda, binwidth = tamanho
         ↪  das barras
        scale_x_continuous(
                breaks = seq(min(vizinhos$n_vizinhos), max(vizinhos$n_vizinhos), by =
                 ↪  1) # Garante que todos os valores de x sejam plotados
        ) +
        labs(
                x = "Número de vizinhos",
                 ↪  # Rótulo do eixo x
                y = "Frequência"
                 ↪  # Rótulo do eixo y
        ) +
        theme_minimal() +                                                           #
         ↪  Aplica tema minimal
        theme(axis.text.x = element_text(angle = 0, hjust = 0.5))                   #
         ↪  Ajusta angulação e ajuste horizontal do texto no eixo x
```

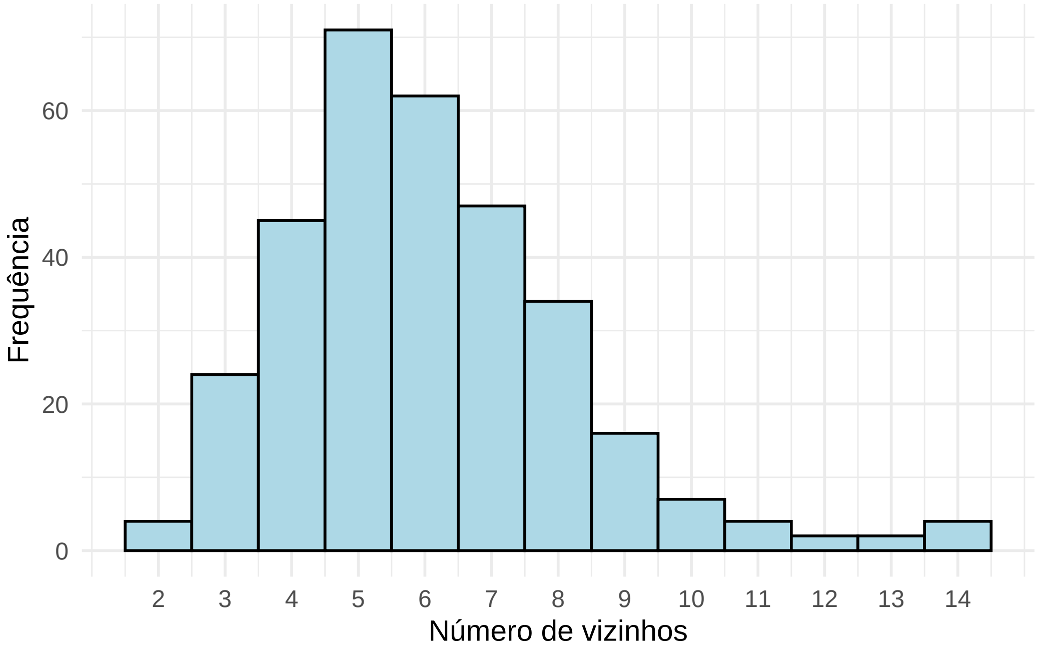

Criar a matriz de pesos espaciais correspondente à vizinhança:

```r
matriz_pesos <- nb2listw(matriz_viz_queen, style = "W", zero.policy = TRUE) # Cria
↪  matriz de pesos: "W" normaliza os pesos por linha, zero.policy = TRUE permite
↪  incluir unidades sem vizinhos
summary(matriz_pesos)                                                     # Exibe
↪  resumo dos pesos espaciais
```

```
Characteristics of weights list object:
Neighbour list object:
Number of regions: 322
Number of nonzero links: 1952
Percentage nonzero weights: 1.882643
Average number of links: 6.062112
Link number distribution:

 2  3  4  5  6  7  8  9 10 11 12 13 14
 4 24 45 71 62 47 34 16  7  4  2  2  4
4 least connected regions:
75 106 320 321 with 2 links
4 most connected regions:
140 234 309 316 with 14 links

Weights style: W
Weights constants summary:
    n     nn  S0       S1       S2
W 322 103684 322 112.8562 1343.689
```

7.2.2. Índice de Moran Global (I Moran)

Avaliar a dependência espacial da variável resposta (RAZAO2) usando I Moran:

```r
# Teste de Moran para RAZAO2
moran_gl <- moran.test(
        setores$RAZAO2,            # Variável de interesse
        matriz_pesos,              # Matriz de pesos espaciais
        alternative = "greater"    # H0: ausência de autocorrelação ou autocorrelação
         ↪  negativa; HA: presença de autocorrelação positiva
)
# Exibir resultado
moran_gl
```

```
    Moran I test under randomisation
```

```
data:  setores$RAZAO2
weights: matriz_pesos

Moran I statistic standard deviate = 9.0954, p-value <
0.00000000000000022
alternative hypothesis: greater
sample estimates:
Moran I statistic       Expectation          Variance
      0.293816737      -0.003115265       0.001065793
```

Abordagem de Monte Carlo para avaliar significância de I Moran:

```
# Realizar a simulação de Monte Carlo
mc_moran_gl <- moran.mc(setores$RAZAO2, # Variável de interesse
                        matriz_pesos,   # Matriz de pesos espaciais
                        nsim = 999)     # Número de simulações aleatórias
# Exibir resultado
mc_moran_gl
```

```
    Monte-Carlo simulation of Moran I

data:  setores$RAZAO2
weights: matriz_pesos
number of simulations + 1: 1000

statistic = 0.29382, observed rank = 1000, p-value = 0.001
alternative hypothesis: greater
```

Criar histograma dos valores de I Moran estimados para padrões simulados na abordagem de Monte Carlo:

```
hist(
        mc_moran_gl$res,                           # Dados simulados de I Moran
        main = "Histograma dos valores simulados de I de Moran", # Título do gráfico
        xlab = "I de Moran (simulado)",            # Rótulo do eixo X
        ylab = "Frequência",                       # Rótulo do eixo Y
        col = "skyblue",                           # Cor das barras do histograma
        border = "white",                          # Cor das bordas das barras
        breaks = 30                                # Número de intervalos (bins) no
         ↪  histograma
)
```

```r
# Adicionar linha representando o valor observado de I Moran
abline(v = mc_moran_gl$statistic,          # Valor de I Moran observado nos dados
 ↪  reais
       col = "red",                        # Cor da linha
       lwd = 2,                            # Espessura da linha
       lty = 2)                            # Tipo de linha (traço-ponto)
text(x = mc_moran_gl$statistic,            # Coordenada X para o texto
     y = max(hist(mc_moran_gl$res,
                  plot = FALSE)$counts),   # Coordenada Y ajustada ao topo do
                   ↪  histograma
     labels = "I observado",               # Texto para indicar o valor observado
     col = "red",                          # Cor do texto
     pos = 4,                              # Posição relativa ao ponto (à direita
      ↪  da linha)
     cex = 0.8)                            # Tamanho do texto
```

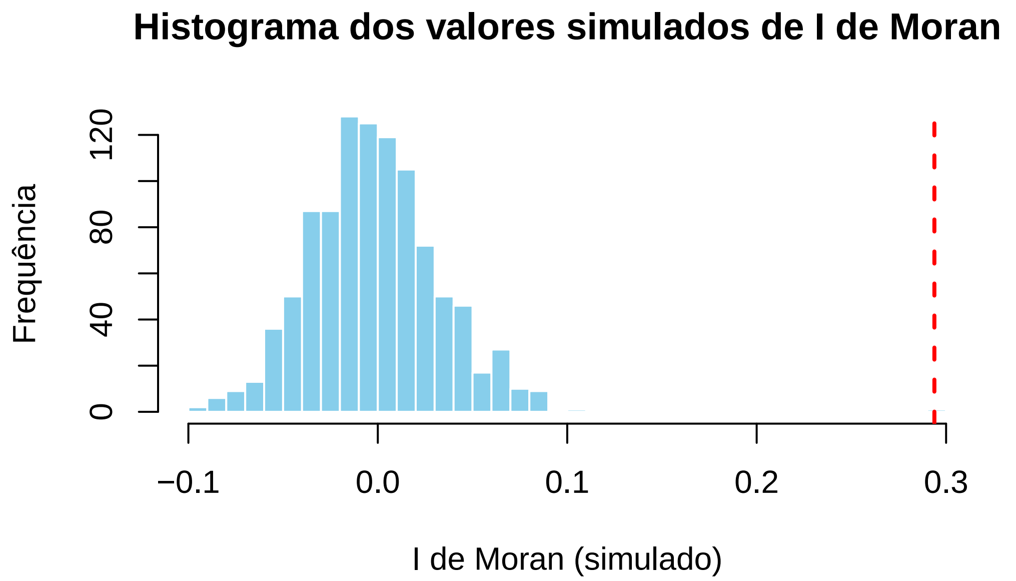

Criar gráfico de dispersão do I Moran para visualizar a autocorrelação espacial dos dados, mostrando como os valores de cada área estão relacionados aos valores médios das áreas vizinhas:

```r
moran.plot(
        setores$RAZAO2,                          # Variável de interesse
        matriz_pesos,                            # Matriz de pesos espaciais
        labels = FALSE,                          # Remove os rótulos das
         ↪  observações
```

```
        main = "Gráfico de dispersão do Índice de Moran", # Título do gráfico
        xlab = "RAZAO2 (Valor observado)",                # Rótulo do eixo X
        ylab = "RAZAO2 (Valor médio dos vizinhos)"        # Rótulo do eixo Y
)
```

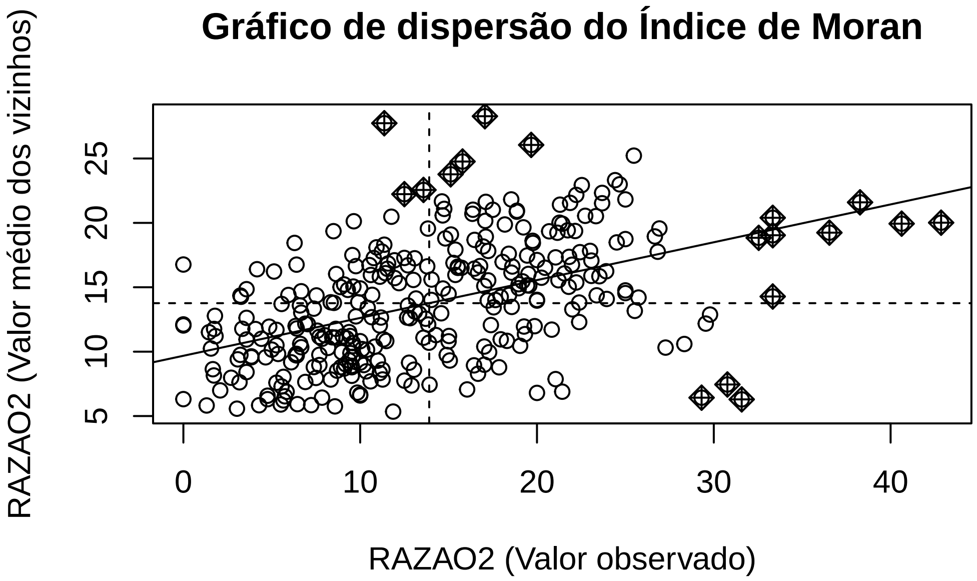

A dependência espacial da variável resposta (neste caso, a prevalência de mães adolescentes) foi analisada por meio do Índice de Moran (I de Moran), que revela padrões não aleatórios na distribuição dos casos de gravidez na adolescência nos setores censitários. Isso indica que os setores próximos tendem a apresentar valores semelhantes de prevalência, sugerindo a formação de agrupamentos espaciais (*clusters*). Esses agrupamentos indicam que fatores espaciais podem estar influenciando as taxas de gravidez entre adolescentes, resultando em uma distribuição espacialmente correlacionada. Para identificar as áreas com *clusters* significativos e *outliers* espaciais, pode-se complementar a análise utilizando o Índice de Moran Local (LISA), que fornece uma visão mais detalhada da variação espacial dentro das diferentes regiões do estudo.

8. Análise Inferencial

8.1. Ajuste do Modelo de Regressão Linear (MRL)

```r
mod.lm <- lm(
        RAZAO2 ~ IBP + PROPM2,   # Fórmula: variável dependente 'RAZAO2' e
 ↪  covariáveis 'IBP' e 'PROPM2'
        data = setores_df        # Base de dados: 'setores_df'
)

# Exibir resumo dos resultados do modelo
summary(mod.lm)
```

```
Call:
lm(formula = RAZAO2 ~ IBP + PROPM2, data = setores_df)

Residuals:
     Min       1Q   Median       3Q      Max 
-16.4347  -4.0440  -0.6814   2.8450  21.2340 

Coefficients:
            Estimate Std. Error t value             Pr(>|t|)    
(Intercept) 15.54712    1.21529  12.793 < 0.0000000000000002 ***
IBP          4.13016    0.28000  14.750 < 0.0000000000000002 ***
PROPM2       0.08857    0.02753   3.217              0.00143 ** 
---
Signif. codes:  0 '***' 0.001 '**' 0.01 '*' 0.05 '.' 0.1 ' ' 1

Residual standard error: 5.86 on 319 degrees of freedom
Multiple R-squared:  0.4317,	Adjusted R-squared:  0.4282 
F-statistic: 121.2 on 2 and 319 DF,  p-value: < 0.00000000000000022
```

8.2. Avaliar a qualidade do ajuste: análise de resíduos

```r
# Colocar os valores ajustados e os resíduos padronizados no banco de dados
 setores_df$valor_aju <- fitted.values(mod.lm)
 setores_df$residuo_pad <- rstandard(mod.lm)
```

Diagrama de dispersão dos resíduos padronizados em função da covariável Índice Brasileiro de Privação:

```
ggplot(setores_df, mapping = aes(x = IBP, y = residuo_pad)) +
        geom_point() +
        geom_hline(yintercept = 0, linetype = "dashed", color = "red") +     # Linha
         ↪  horizontal em y=0
        geom_hline(yintercept = -1.96, linetype = "dashed", color = "red") + # Linha
         ↪  horizontal em y=-1,96
        geom_hline(yintercept = 1.96, linetype = "dashed", color = "red") +  # Linha
         ↪  horizontal em y=1,96
        labs(
                x = "Índice Brasileiro de Privação",                         # Rótulo
                 ↪  do eixo x
                y = "Resíduos padronizados"                                  # Rótulo
                 ↪  do eixo y
        ) +
        theme_minimal()                                                      # Aplica
         ↪  tema minimal
```

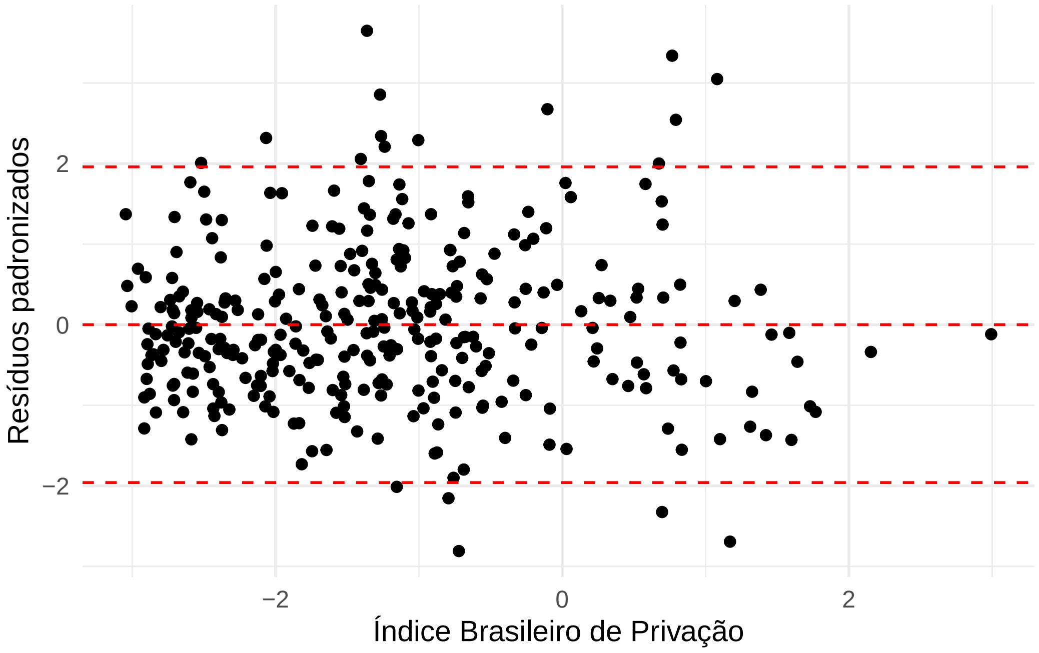

Diagrama de dispersão dos resíduos padronizados em função da covariável Proporção de mulheres responsáveis pelo domicílio:

```
ggplot(setores_df, mapping = aes(x = PROPM2, y = residuo_pad)) +
        geom_point() +
        geom_hline(yintercept = 0, linetype = "dashed", color = "red") +      # Linha
         ↪  horizontal em y=0
        geom_hline(yintercept = -1.96, linetype = "dashed", color = "red") +  # Linha
         ↪  horizontal em y=-1,96
```

```
    geom_hline(yintercept = 1.96, linetype = "dashed", color = "red") +   # Linha
     ↪  horizontal em y=1,96
    labs(
            x = "Proporção de mulheres responsáveis pelo domicílio",
             ↪  # Rótulo do eixo x
            y = "Resíduos padronizados"                                    #
             ↪  Rótulo do eixo y
    ) +
    theme_minimal()                                                        #
     ↪  Aplica tema minimal
```

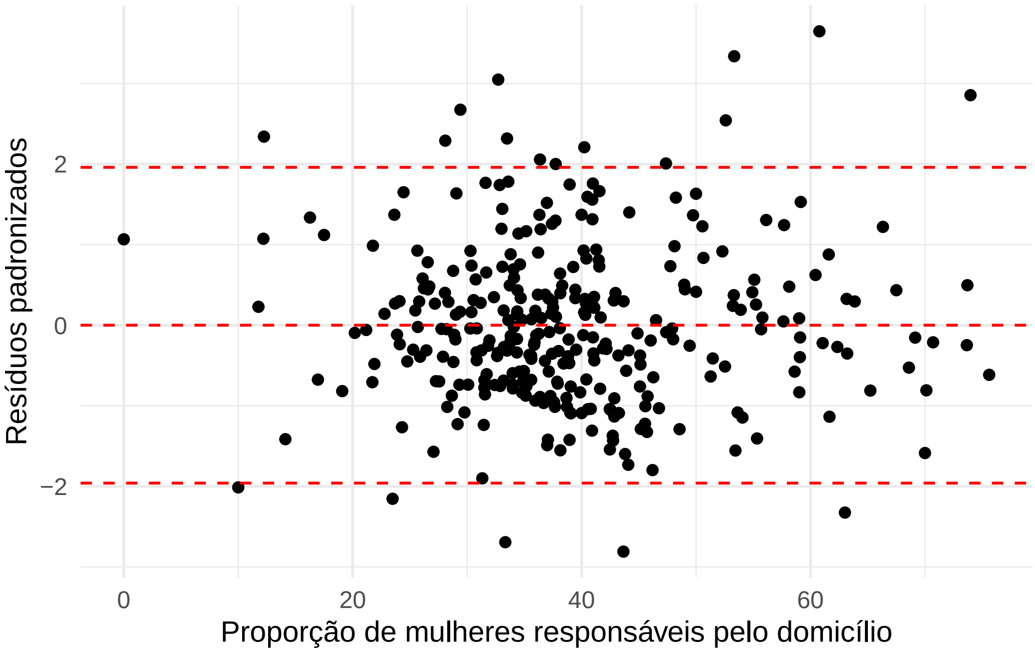

Diagrama de dispersão dos resíduos padronizados em função dos valores ajustados:

```
ggplot(setores_df, mapping = aes(x=valor_aju, y=residuo_pad)) +
    geom_point() +
    geom_hline(yintercept = 0, linetype = "dashed", color = "red") +      # Linha
     ↪  horizontal em y=0
    geom_hline(yintercept = -1.96, linetype = "dashed", color = "red") + # Linha
     ↪  horizontal em y=-1,96
    geom_hline(yintercept = 1.96, linetype = "dashed", color = "red") +  # Linha
     ↪  horizontal em y=1,96
    labs(
            x = "Valores ajustados",                                     # Rótulo
             ↪  do eixo x
            y = "Resíduos padronizados"                                  # Rótulo
             ↪  do eixo y
```

```
    ) +
    theme_minimal()                                                  # Aplica
     ↪  tema minimal
```

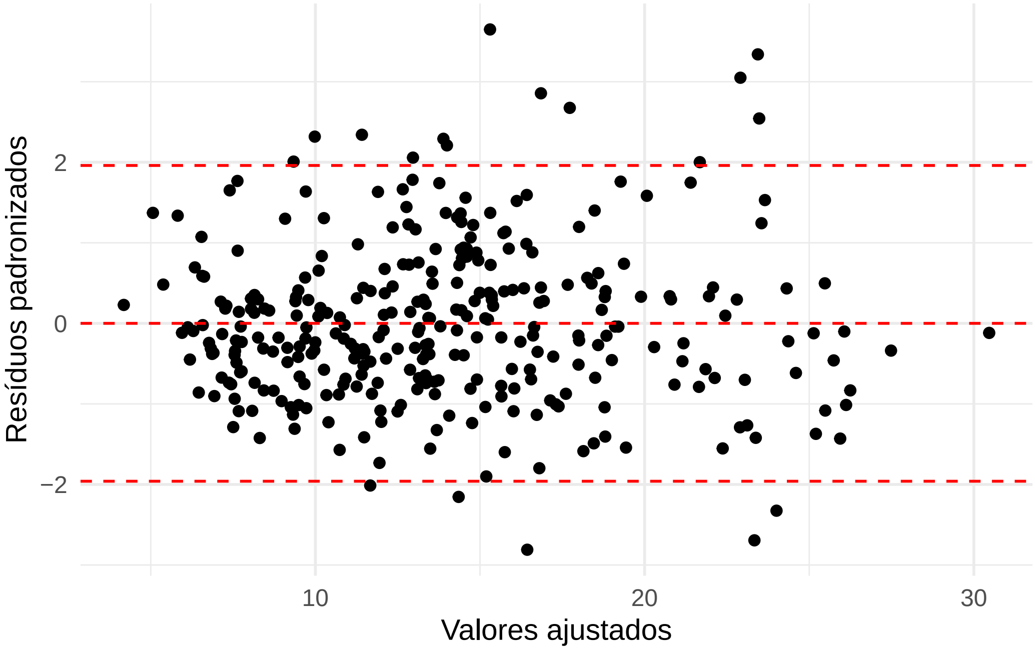

Nos três gráficos, os resíduos apresentam-se distribuídos aleatoriamente em torno de zero, sem evidência de heterocedasticidade, com alguns *outliers*.

Histograma dos resíduos padronizados:

```
ggplot(setores_df, aes(x = residuo_pad)) +
    geom_histogram(fill = "lightblue", color = "black", bins = 20) + # Cria o
     ↪  histograma com cor de preenchimento e borda, bins = número de barras
    labs(
        x = "Resíduo padronizado",                                 # Título do
         ↪  eixo x
        y = "Número de setores"                                    # Título do
         ↪  eixo y
    ) +
    theme_minimal()                                                # Aplica o
     ↪  tema minimal
```

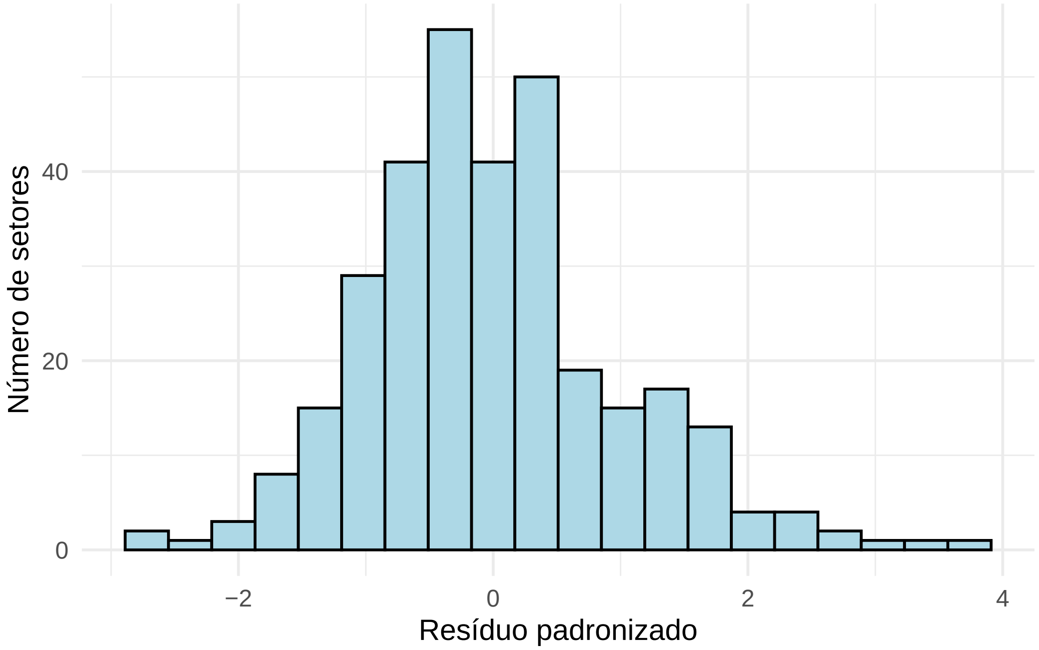

Qq-plot para os resíduos:

```
hnp(rstandard(mod.lm))
```

```
Half-normal plot with simulated envelope generated assuming the residuals are
        normally distributed under the null hypothesis.
```

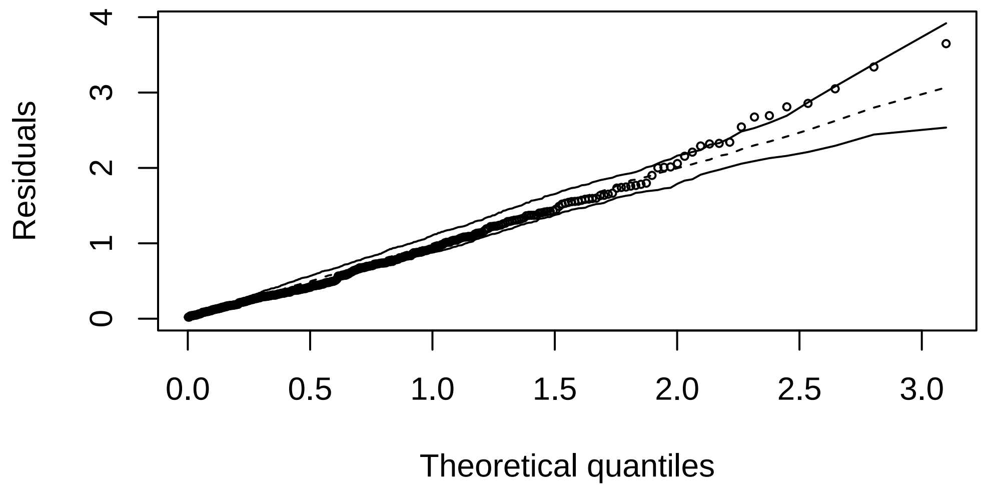

Teste de normalidade dos resíduos:

```
ks.test(setores_df$residuo_pad, "pnorm")
```

```
	Asymptotic one-sample Kolmogorov-Smirnov test

data:  setores_df$residuo_pad
D = 0.068375, p-value = 0.0985
alternative hypothesis: two-sided
```

Teste de Moran para os resíduos do modelo:

O teste de Moran foi aplicado aos resíduos do modelo, considerando como hipótese nula I de Moran = 0 e como hipótese alternativa I de Moran > 0, correspondente ao argumento ' alternative = "greater"', que é o padrão da função 'lm.morantest'. Essa configuração está alinhada ao interesse do presente estudo em detectar autocorrelação espacial positiva

```
lm.morantest(mod.lm, matriz_pesos) # Teste de Moran para os resíduos modelo OLS
```

```
	Global Moran I for regression residuals
```

```
data:
model: lm(formula = RAZAO2 ~ IBP + PROPM2, data = setores_df)
weights: matriz_pesos

Moran I statistic standard deviate = 1.8519, p-value = 0.03202
alternative hypothesis: greater
sample estimates:
Observed Moran I      Expectation         Variance
     0.054899635     -0.005263698      0.001055445
```

A identificação de dependência espacial nos resíduos da regressão linear indica que, mesmo após considerar as variáveis explicativas no modelo, ainda há padrões espaciais não explicados. Isso é uma violação dos pressupostos da regressão linear clássica, que presume que os resíduos sejam independentes e identicamente distribuídos (i.i.d.). A presença de dependência nos resíduos sugere que o modelo inicial não capturou totalmente a estrutura espacial dos dados.

8.3. Diagnóstico de Lagrange (LM)

Realizar o Diagnóstico de Lagrange (LM) para detectar dependência espacial nos resíduos do MRL:

```r
razao.lagr <- lm.RStests(mod.lm, matriz_pesos, test = c("LMerr", "RLMerr", "LMlag",
↪  "RLMlag"))

# Exibir os resultados completos dos testes LM
razao.lagr
```

```

    Rao's score (a.k.a Lagrange multiplier) diagnostics for spatial
    dependence

data:
model: lm(formula = RAZAO2 ~ IBP + PROPM2, data = setores_df)
test weights: matriz_pesos

RSerr = 2.769, df = 1, p-value = 0.09611


    Rao's score (a.k.a Lagrange multiplier) diagnostics for spatial
    dependence
```

```
data:
model: lm(formula = RAZAO2 ~ IBP + PROPM2, data = setores_df)
test weights: matriz_pesos

adjRSerr = 0.014537, df = 1, p-value = 0.904


    Rao's score (a.k.a Lagrange multiplier) diagnostics for spatial
    dependence

data:
model: lm(formula = RAZAO2 ~ IBP + PROPM2, data = setores_df)
test weights: matriz_pesos

RSlag = 3.8674, df = 1, p-value = 0.04923


    Rao's score (a.k.a Lagrange multiplier) diagnostics for spatial
    dependence

data:
model: lm(formula = RAZAO2 ~ IBP + PROPM2, data = setores_df)
test weights: matriz_pesos

adjRSlag = 1.1129, df = 1, p-value = 0.2914
```

```r
# Acessar e exibir o p-valor para o teste LM de erro espacial
p_val_RSerr <- razao.lagr$RSerr["p.value"]
print(paste("p-valor do teste LMerr (Erro espacial):", p_val_RSerr))
```

```
[1] "p-valor do teste LMerr (Erro espacial): c(0.0961052612639425)"
```

```r
p_val_RSlag <- razao.lagr$RSlag["p.value"] # Acessar p-valor Teste de Lagrange
 ↪  Multiplier para erro espacial
print(p_val_RSlag)
```

```
$p.value

0.04923201
```

```r
# Acessar e exibir o p-valor para o teste LM de defasagem espacial
p_val_RSlag <- razao.lagr$RSlag["p.value"] # Testa se há dependência espacial nos
 ↪  valores ajustados
print(paste("p-valor do teste LMlag (Defasagem espacial):", p_val_RSlag))
```

```
[1] "p-valor do teste LMlag (Defasagem espacial): c(0.04923200587906)"
```

- LMerr (RSerr): Teste de Lagrange Multiplier para erro espacial (teste se os resíduos têm autocorrelação espacial que pode ser modelada com um modelo de erro espacial).
- RLMerr (adjRSerr): Teste robusto de Lagrange Multiplier para erro espacial.
- LMlag (RSlag): Teste de Lagrange Multiplier para defasagem espacial (teste se a variável resposta tem autocorrelação espacial que pode ser modelada com um modelo de defasagem espacial).
- RLMlag (adjRSlag): Teste robusto de Lagrange Multiplier para defasagem espacial.

Os testes de Lagrange Multiplier auxiliam a identificar qual tipo de dependência espacial está presente. Eles distinguem se a dependência ocorre na variável dependente, caso em que o teste LMlag favorece a especificação de um modelo SAR, ou se está concentrada nos termos de erro, situação em que o teste LMerr indica a adequação de um modelo SEM. Quando ambos os efeitos podem estar presentes, os testes robustos RLMlag e RLMerr permitem verificar qual componente é mais relevante. Para ver o passo a passo do processo de escolha do modelo, consulte o fluxograma do artigo, na Figura 1.

No nosso exemplo, o teste LMlag apresentou valor de p menor que 0.05, enquanto o teste LMerr apresentou valor de p maior que 0.05. Dessa forma, a indicação foi pela adoção do modelo SAR.

8.4. Ajuste do Modelo de Defasagem Espacial (SAR)

Ajustar o modelo de defasagem espacial SAR considerando as variáveis explicativas 'IBP' e 'PROPM2':

```r
mod.lag <- lagsarlm(RAZAO2 ~ IBP + PROPM2, # Ajusta modelo de defasagem espacial
                    data = setores,
                    listw = matriz_pesos)  # A matriz de pesos espaciais
                     ↪  'matriz_pesos' é usada para levar em conta a dependência
                     ↪  espacial

# Exibe resultados do modelo de defasagem espacial
summary(mod.lag)
```

```
Call:
lagsarlm(formula = RAZAO2 ~ IBP + PROPM2, data = setores, listw = matriz_pesos)

Residuals:
      Min        1Q    Median        3Q       Max
-16.66564  -3.90251  -0.78655   2.83319  20.47044

Type: lag
Coefficients: (asymptotic standard errors)
             Estimate Std. Error z value              Pr(>|z|)
(Intercept) 13.266455   1.705430  7.7790  0.000000000000007327
IBP          3.779434   0.332289 11.3739 < 0.00000000000000022
PROPM2       0.086182   0.027212  3.1671               0.00154

Rho: 0.14094, LR test value: 3.6748, p-value: 0.055239
Asymptotic standard error: 0.072717
    z-value: 1.9383, p-value: 0.052591
Wald statistic: 3.7569, p-value: 0.052591

Log likelihood: -1022.878 for lag model
ML residual variance (sigma squared): 33.516, (sigma: 5.7893)
Number of observations: 322
Number of parameters estimated: 5
AIC: 2055.8, (AIC for lm: 2057.4)
LM test for residual autocorrelation
test value: 0.011308, p-value: 0.91531
```

```
# Calcula e exibe intervalos de confiança para os coeficientes do modelo de defasagem
↪  espacial
confint(mod.lag)
```

```
                   2.5 %     97.5 %
rho         -0.001578157  0.2834654
(Intercept)  9.923873919 16.6090368
IBP          3.128159315  4.4307084
PROPM2       0.032848134  0.1395156
```

Os coeficientes indicam que a cada aumento de uma unidade no Índice Brasileiro de Privação (IBP) espera-se um aumento médio de 3,78 % na prevalência de mães adolescentes (RAZAO2), independente da proporção de mulheres responsáveis pelo domicílio. De forma semelhante, a cada aumento de um ponto percentual na proporção de mulheres responsáveis pelo domicílio

espera-se um aumento médio de 0,086 % na prevalência de mães adolescentes, independente do IBP.

8.5. Análise de resíduos do modelo de defasagem espacial

```
# Colocar os valores ajustados e os resíduos padronizados no banco de dados
setores_df$valor_aju_lag <- fitted.values(mod.lag)
```

```
This method assumes the response is known - see manual page
```

```
setores_df$residuo_pad_lag <- (mod.lag$residuals -
↪  mean(mod.lag$residuals))/sqrt(mod.lag$s2)
```

Diagrama de dispersão dos resíduos padronizados em função da covariável Índice Brasileiro de Privação:

```
ggplot(setores_df, mapping = aes(x = IBP, y = residuo_pad_lag)) +
        geom_point() +
        geom_hline(yintercept = 0, linetype = "dashed", color = "red") +     # Linha
         ↪  horizontal em y=0
        geom_hline(yintercept = -1.96, linetype = "dashed", color = "red") + # Linha
         ↪  horizontal em y=-1.96
        geom_hline(yintercept = 1.96, linetype = "dashed", color = "red") +  # Linha
         ↪  horizontal em y=1.96
        labs(
                x = "Índice Brasileiro de Privação",                           #
                 ↪  Rótulo do eixo x
                y = "Resíduos padronizados"                                    #
                 ↪  Rótulo do eixo y
        ) +
        theme_minimal()                                                        # Aplica
         ↪  tema minimal
```

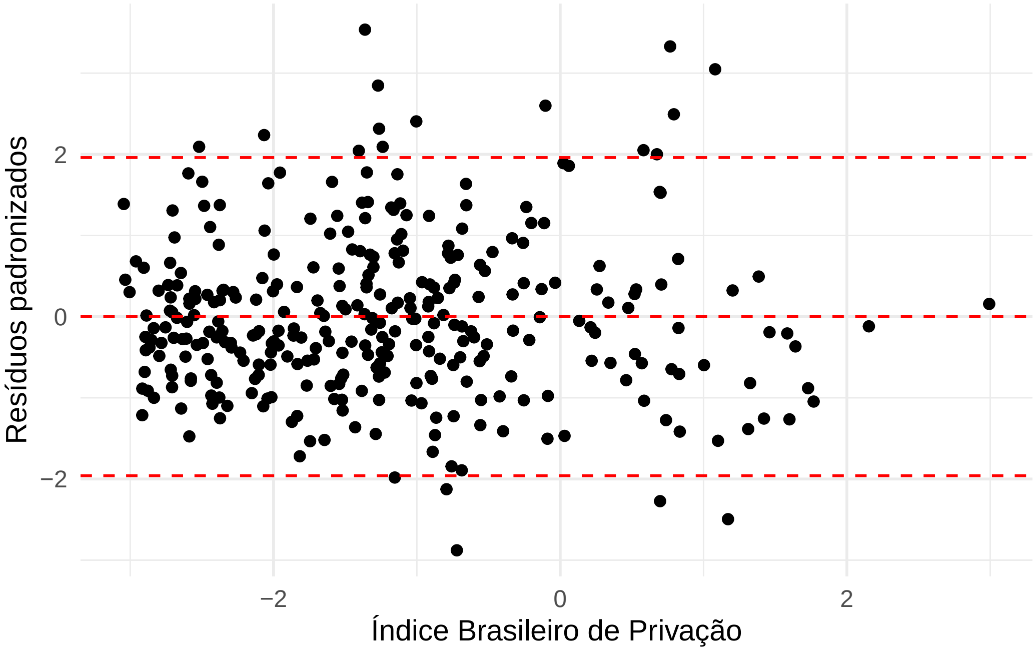

Diagrama de dispersão dos resíduos padronizados em função da covariável Proporção de mulheres responsáveis pelo domicílio:

```
ggplot(setores_df, mapping = aes(x = PROPM2, y = residuo_pad_lag)) +
        geom_point() +
        geom_hline(yintercept = 0, linetype = "dashed", color = "red") +      # Linha
        ↪  horizontal em y=0
        geom_hline(yintercept = -1.96, linetype = "dashed", color = "red") +  # Linha
        ↪  horizontal em y=-1,96
        geom_hline(yintercept = 1.96, linetype = "dashed", color = "red") +   # Linha
        ↪  horizontal em y=1,96
        labs(
                x = "Proporção de mulheres responsáveis pelo domicílio",
                ↪  # Rótulo do eixo x
                y = "Resíduos padronizados"                                   #
                ↪  Rótulo do eixo y
        ) +
        theme_minimal()                                                       #
        ↪  Aplica tema minimal
```

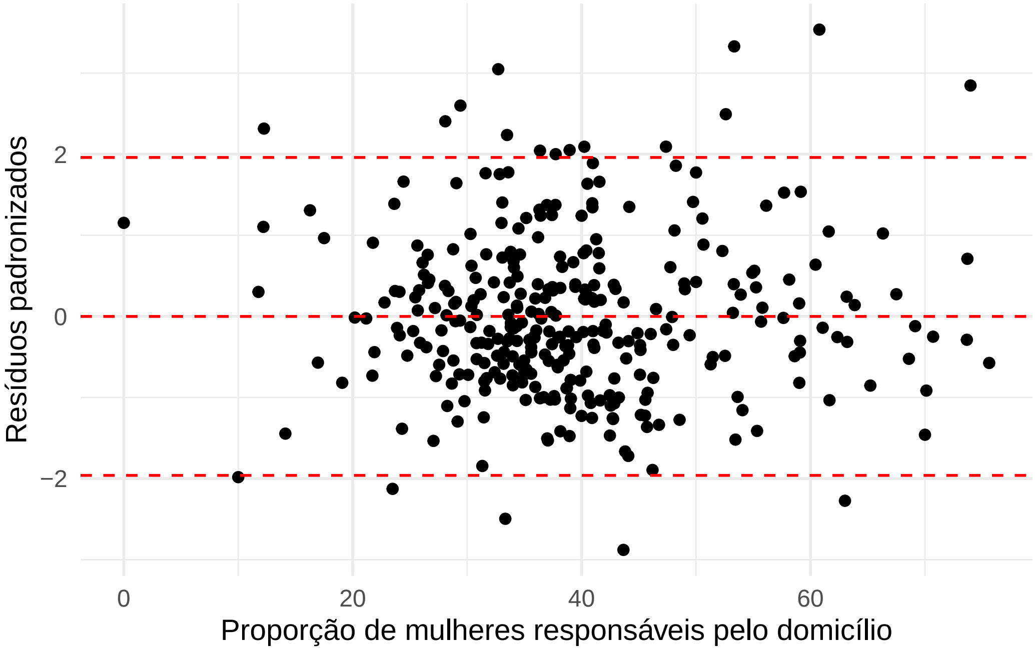

Diagrama de dispersão dos resíduos padronizados em função dos valores ajustados:

```
ggplot(setores_df, mapping = aes(x=valor_aju_lag, y=residuo_pad_lag)) +
        geom_point() +
        geom_hline(yintercept = 0, linetype = "dashed", color = "red") +     # Linha
         ↪  horizontal em y=0
        geom_hline(yintercept = -1.96, linetype = "dashed", color = "red") + # Linha
         ↪  horizontal em y=-1.96
        geom_hline(yintercept = 1.96, linetype = "dashed", color = "red") +  # Linha
         ↪  horizontal em y=1.96
        labs(
                x = "Valores ajustados",                                     # Rótulo
                 ↪  do eixo x
                y = "Resíduos padronizados"                                  # Rótulo
                 ↪  do eixo y
        ) +
        theme_minimal()                                                      # Aplica
         ↪  tema minimal
```

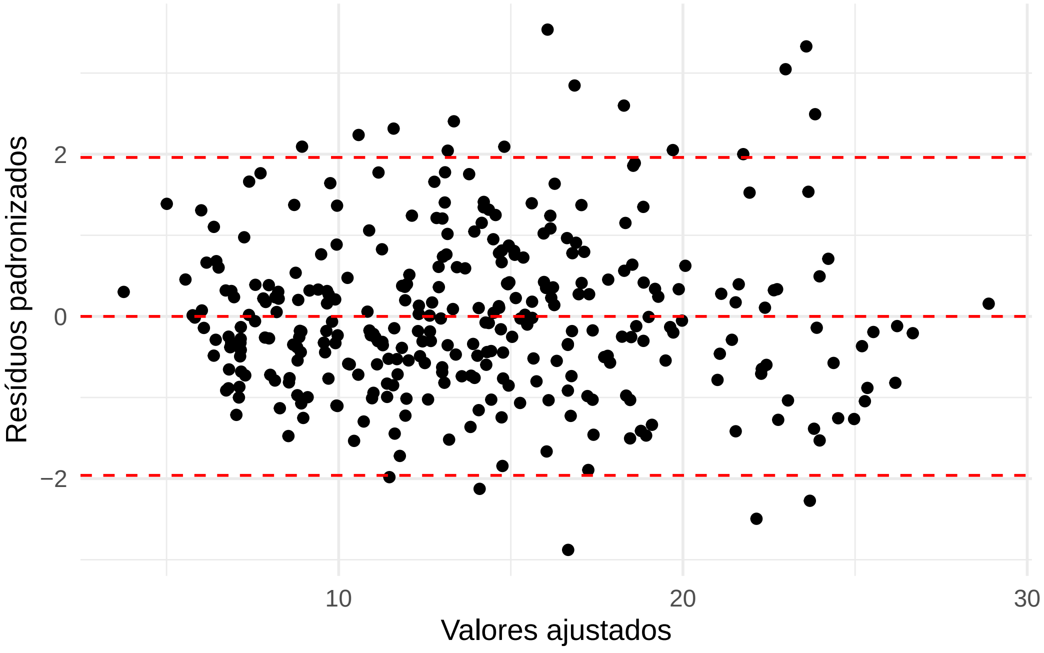

Nos três gráficos, os resíduos apresentam-se distribuídos aleatoriamente em torno de zero, sem evidência de heterocedasticidade, com alguns *outliers*.

Histograma dos resíduos padronizados:

```
ggplot(setores_df, aes(x = residuo_pad_lag)) +                          # Define a
↪  variável RAZAO2 no eixo x
       geom_histogram(fill = "lightblue", color = "black", bins = 20) + # Cria o
        ↪  histograma com cor de preenchimento e borda, bins = número de barras
       labs(
              x = "Resíduo padronizado",                                # Título do
               ↪  eixo x
              y = "Número de setores"                                   # Título do
               ↪  eixo y
       ) +
       theme_minimal()                                                  # Aplica o
        ↪  tema minimal
```

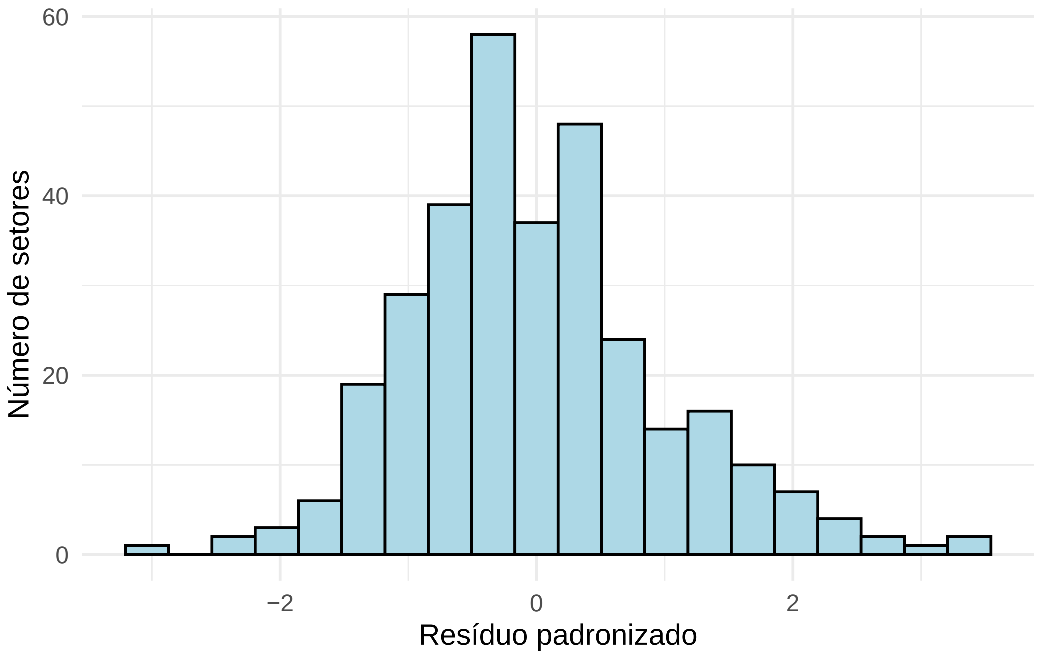

O histograma dos resíduos padronizados apresenta distribuição aproximadamente simétrica e centrada em zero, compatível com uma distribuição normal. Há poucos valores extremos, e não se observa assimetria acentuada ou padrões irregulares, indicando boa adequação do modelo às suposições de normalidade dos resíduos".

Qq-plot para os resíduos:

```
ggplot(setores_df, aes(sample = residuo_pad_lag)) +
        stat_qq() +                                          # Plota os pontos dos
         ↪  quantis observados x teóricos da normal
        stat_qq_line() +                                     # Adiciona linha de
         ↪  referência para indicar distribuição normal ideal
        labs(y = "Resíduo padronizado",                      # Título do eixo Y
             x = "Quantis da distribuição normal padrão")    # Título do eixo X
```

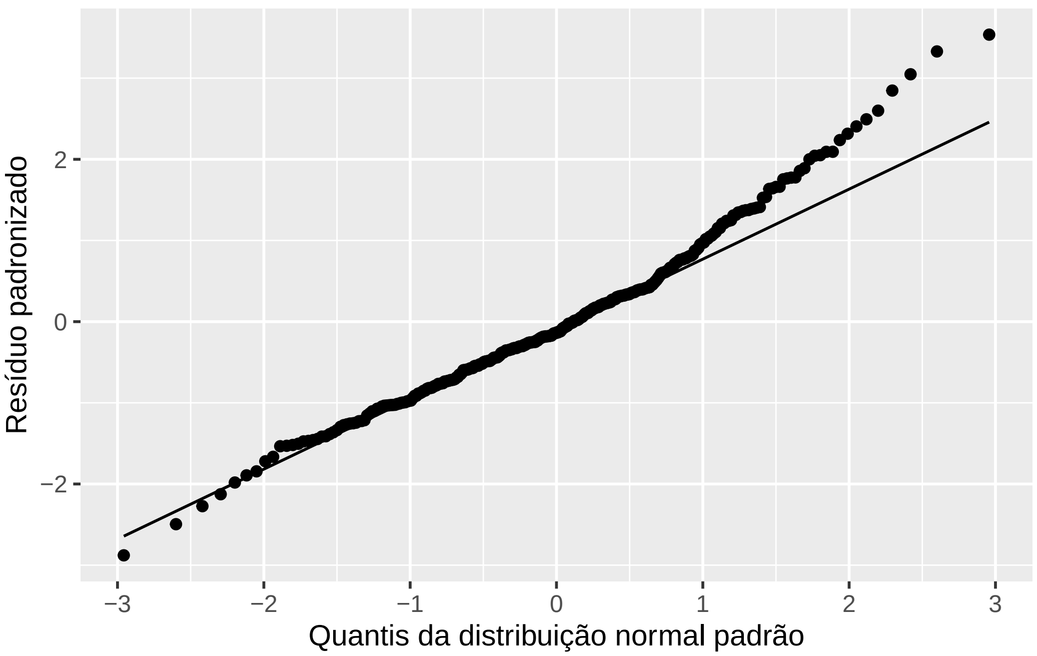

Teste de normalidade dos resíduos:

```
ks.test(setores_df$residuo_pad_lag, "pnorm")
```

```

	Asymptotic one-sample Kolmogorov-Smirnov test

data:  setores_df$residuo_pad_lag
D = 0.074759, p-value = 0.05468
alternative hypothesis: two-sided
```

O qq-plot sugere algum desvio da Normal, mas, segundo o teste de normalidade, não há evidências suficientes para rejeitar a hipótese de normalidade.

Teste de Moran para os resíduos do modelo:

```
moran.test(mod.lag$residuals, matriz_pesos)
```

```

	Moran I test under randomisation

data:  mod.lag$residuals
```

```
weights: matriz_pesos

Moran I statistic standard deviate = 0.033434, p-value = 0.4867
alternative hypothesis: greater
sample estimates:
Moran I statistic       Expectation          Variance
     -0.002024184      -0.003115265       0.001064957
```

De acordo com o teste de Moran, os resíduos do modelo não apresentam autocorrelação espacial significativa (Moran's I = -0.002, p = 0.487). Isso indica que, após o ajuste, a dependência espacial foi eliminada.

B. Utilizando o software GeoDA

1. Dependência espacial da variável resposta (análogo ao item A.7.2.)

1.1. Matriz de vizinhança espacial (análogo ao item A.7.2.1)

Criar a matriz de vizinhança do tipo "queen" (considera vizinhos que compartilham borda ou vértice) e criar a matriz de pesos espaciais correspondente à vizinhança:

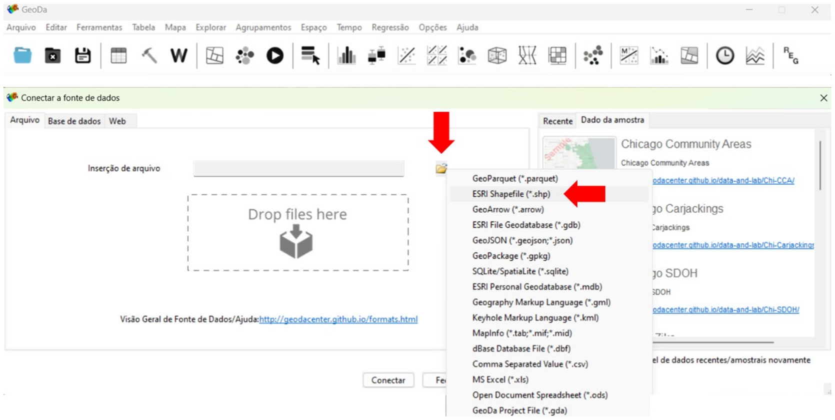

Figure 1: Abrindo a camada shapefile

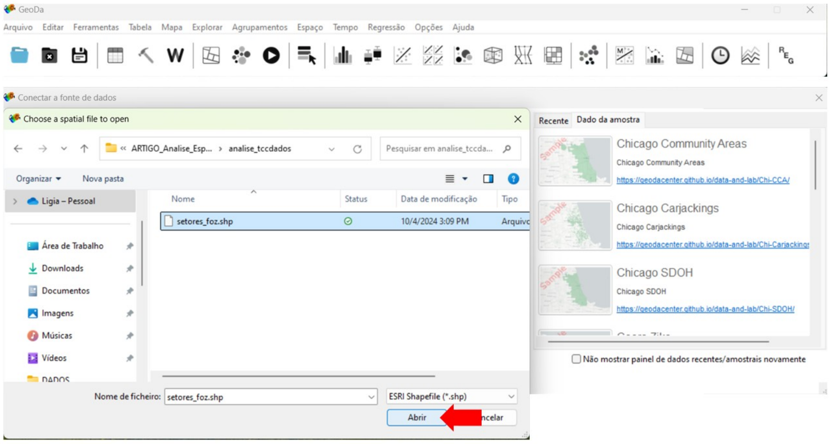

Figure 2: Selecionando a malha de polígonos que será lida pelo GeoDa (neste caso, a malha de setores).

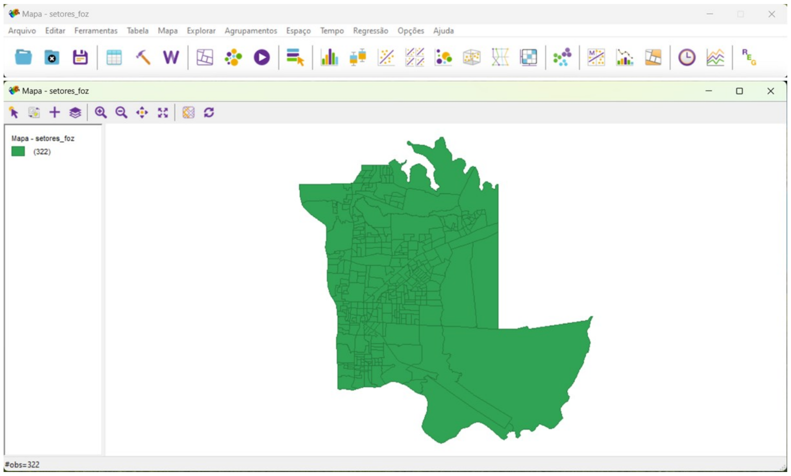

Figure 3: Visualizando a malha de polígonos

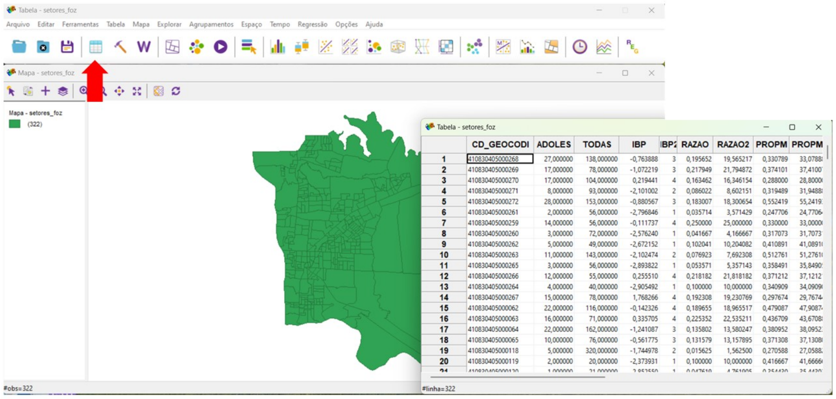

Figure 4: Abrindo a tabela de atributos

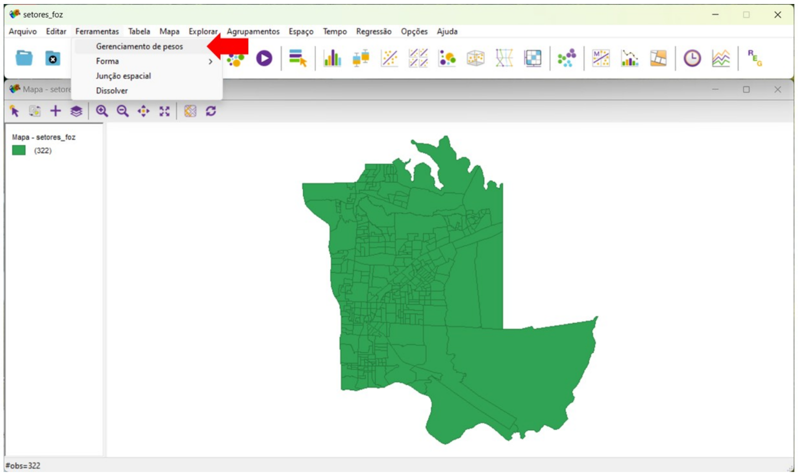

Figure 5: Criando a matriz de vizinhança: clicando no ícone "Ferramentas" e depois em "Gerenciamento de pesos"

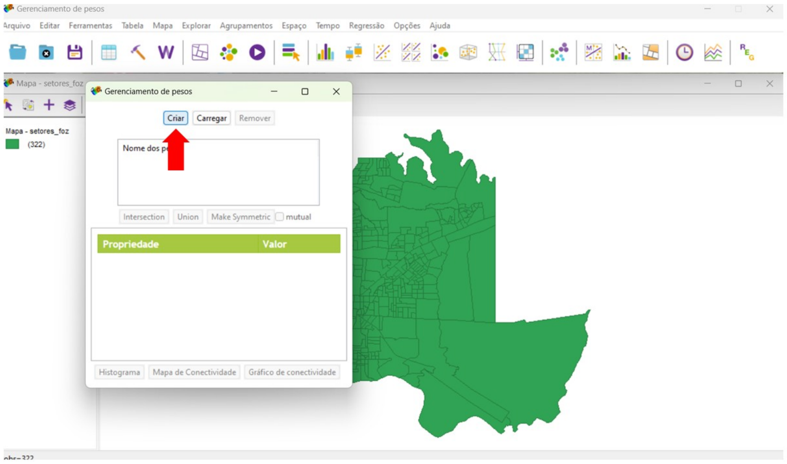

Figure 6: Clicando em “Criar”

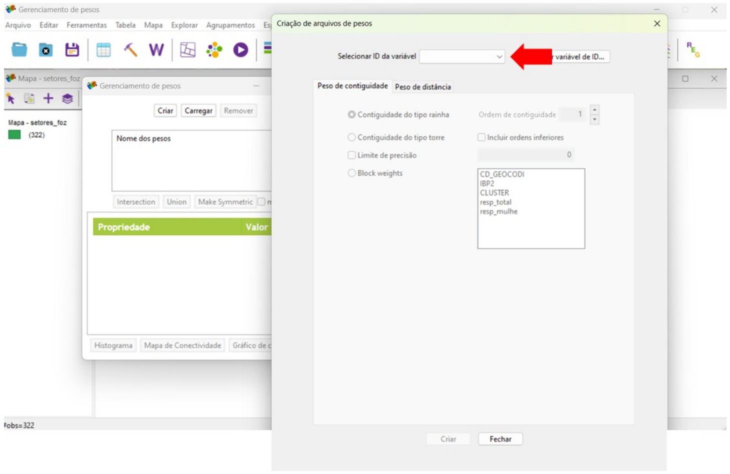

Figure 7: Selecionando a variável que contém o ID único para cada polígono

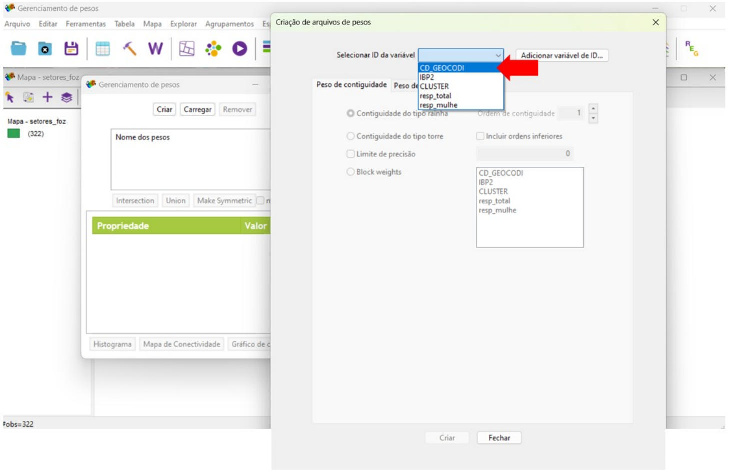

Figure 8: Selecionando a variável “CD_GEOCODI”

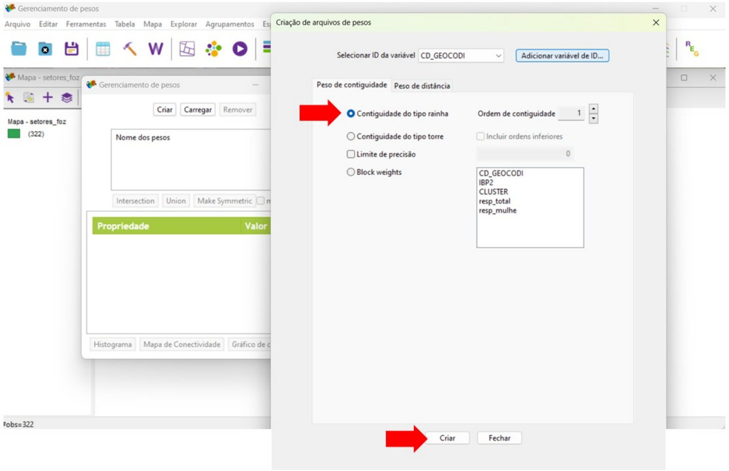

Figure 9: Escolhendo a opção “Contiguidade do tipo Rainha” e clicando em “Criar”

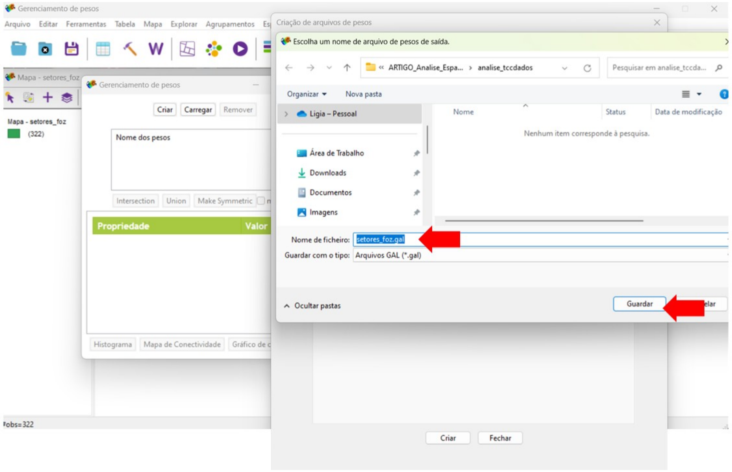

Figure 10: Salvando a matriz de vizinhança com um nome

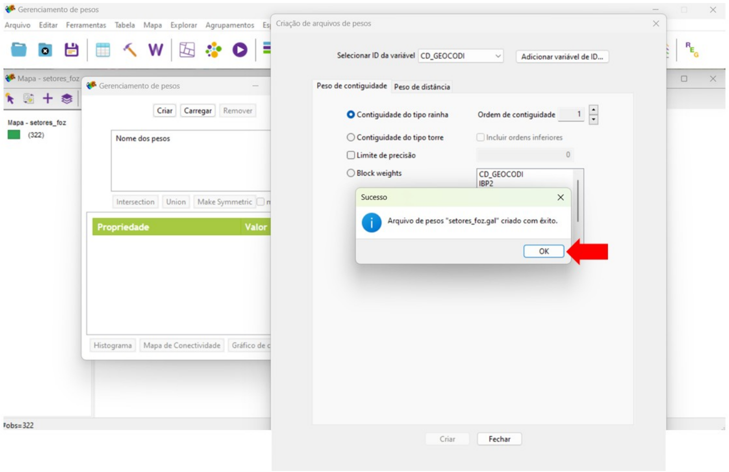

Figure 11: Clicando em “OK”

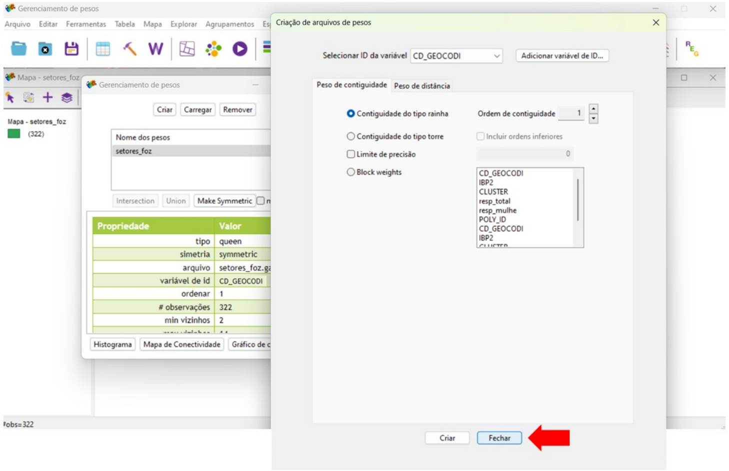

Figure 12: Clicando em “Fechar”

Criar histograma mostrando a distribuição do número de vizinhos por setor censitário:

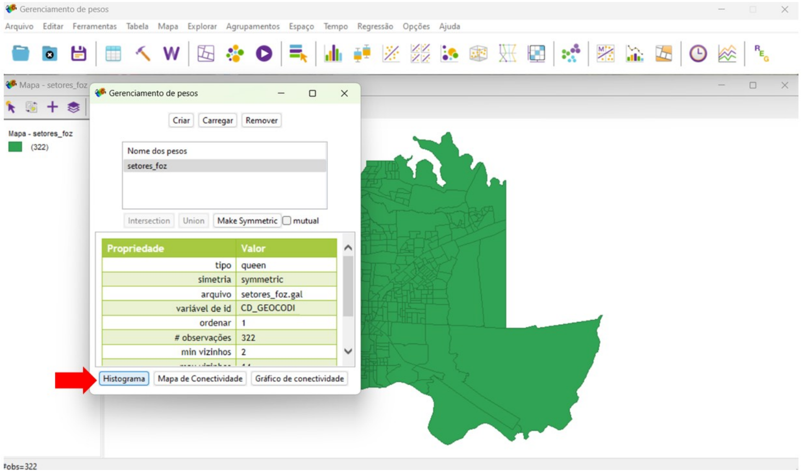

Figure 13: Clicando em “Histograma” na janela de Gerenciamento de pesos

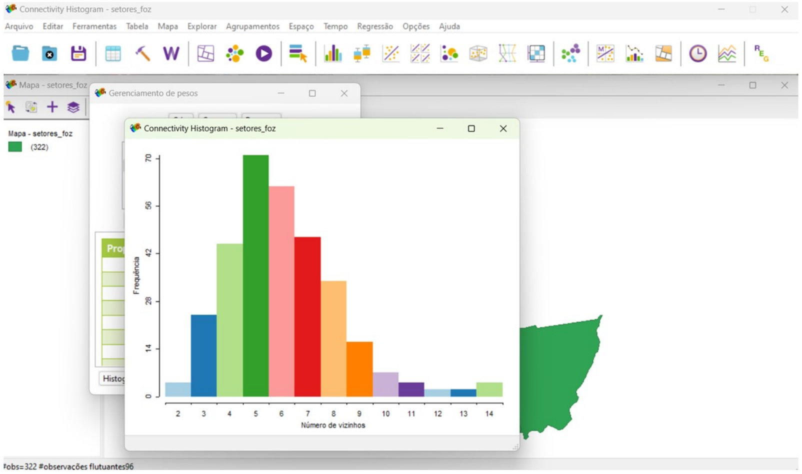

Figure 14: Visualizando o histograma da matriz de vizinhança

Plotar mapa de conectividade de vizinhos com base na contiguidade "queen":

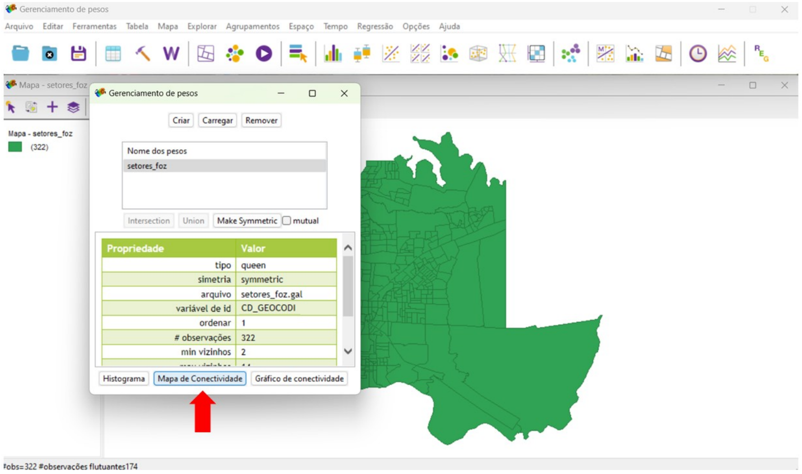

Figure 15: Clicando em "Mapa de Conectividade" da matriz de vizinhança

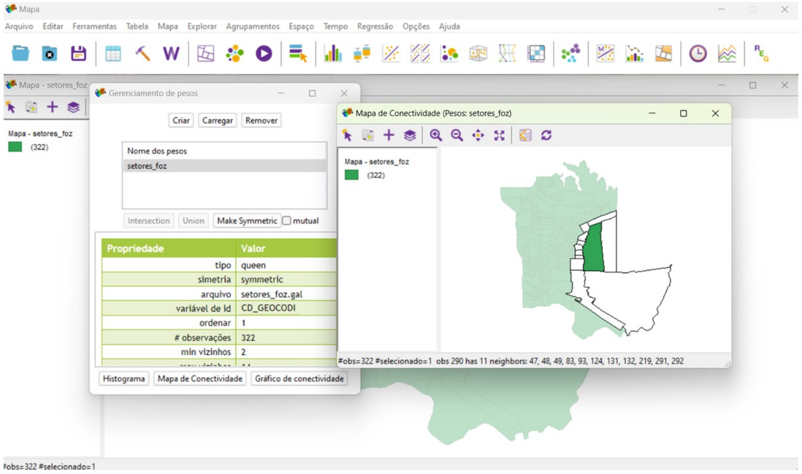

Figure 16: Visualizando o Mapa de Conectividade: passando o mouse sobre os polígonos para identificar seus vizinhos considerados pela matriz

Plotar gráfico de conectividade de vizinhos com base na contiguidade “queen”:

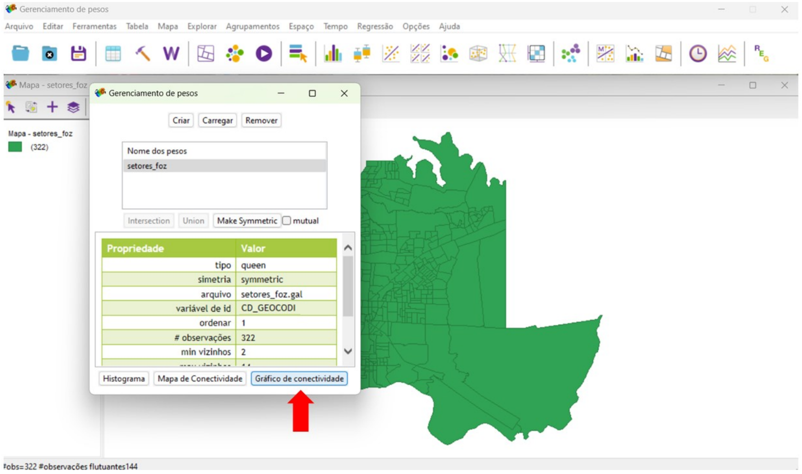

Figure 17: Clicando em “Gráfico de Conectividade” da matriz de vizinhança

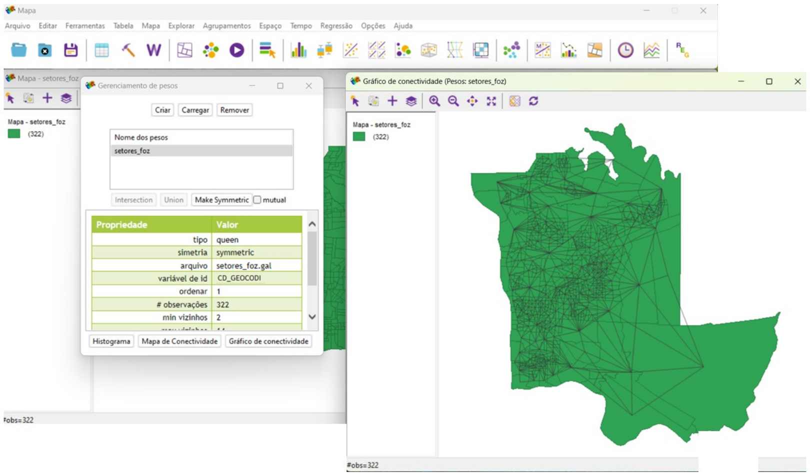

Figure 18: Visualizando o Gráfico de Conectividade

1.2. Índice de Moran Global (I Moran) (análogo ao item A.7.2.2)

Avaliar a dependência espacial da variável resposta (RAZAO2) usando Índice de Moran Global (I Moran):

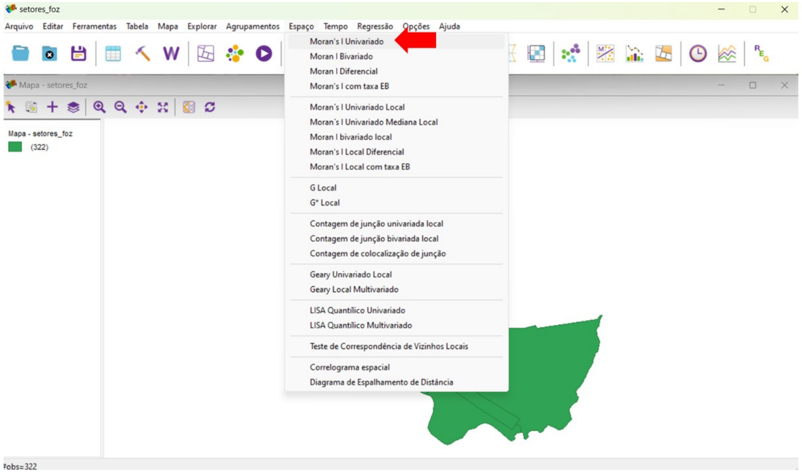

Figure 19: Clicando no ícone "Espaço" e selecionando "Moran's I Univariado"

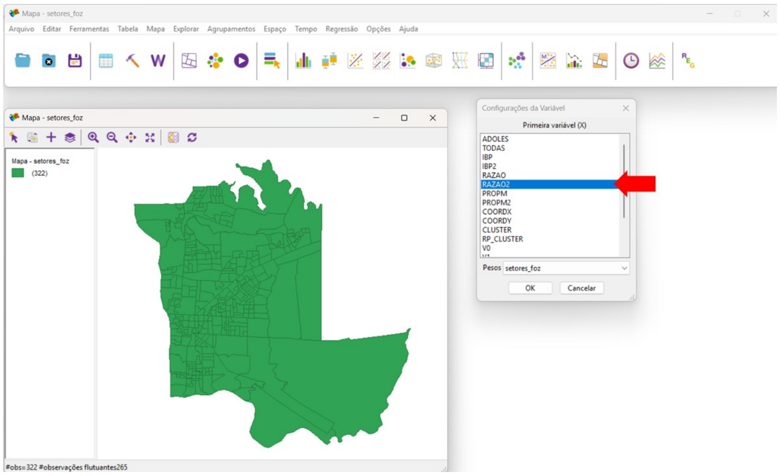

Figure 20: Selecionando a variável “RAZAO2”

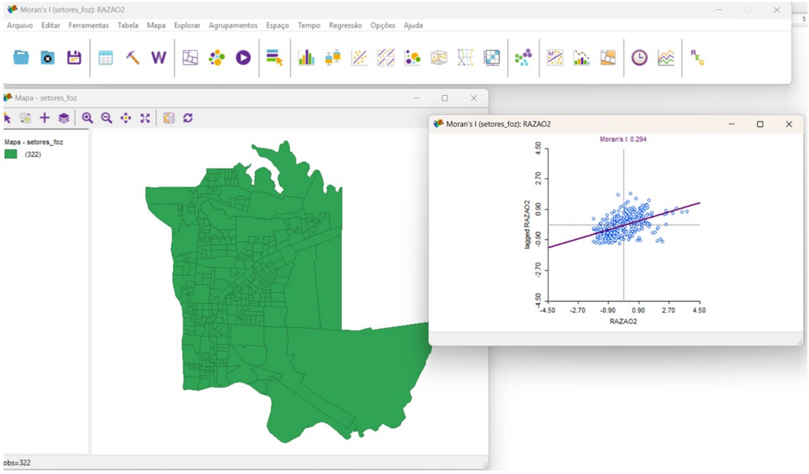

Figure 21: Visualizando o Moran Global da variável “RAZAO2”

Abordagem de Monte Carlo para avaliar significância de I Moran:

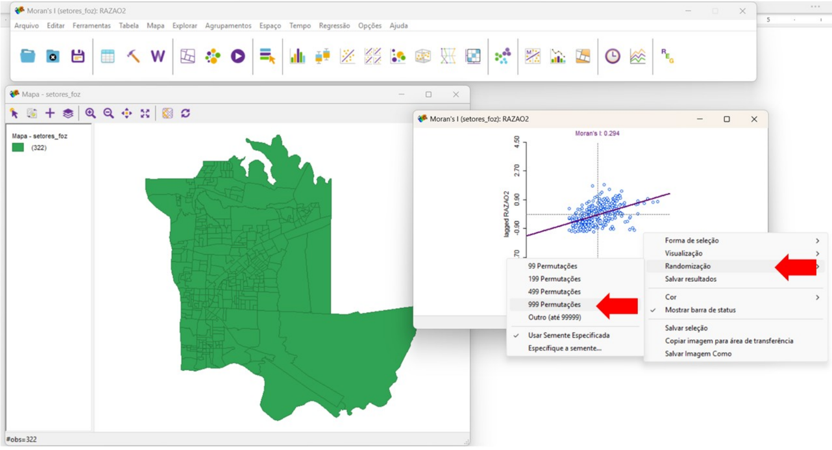

Figure 22: Clicando com o botão direito sobre o mapa, escolhendo 999 permutações e a opção “Randomização”

Criar histograma dos valores de I Moran estimados para padrões simulados na abordagem de Monte Carlo e criar gráfico de dispersão do I Moran para visualizar a autocorrelação espacial dos dados, mostrando como os valores de cada área estão relacionados aos valores médios das áreas vizinhas:

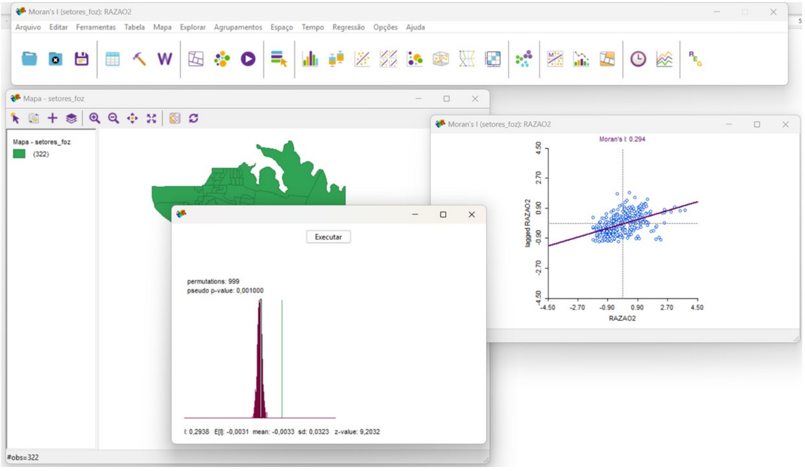

Figure 23: Visualizando o histograma resultante da permutação e o pseudo p-valor

O Índice de Moran indicou presença de dependência espacial da variável resposta (neste caso, a prevalência de mães adolescentes). Esse resultado aponta que áreas vizinhas tendem a apresentar prevalências semelhantes, evidenciando a formação de agrupamentos espaciais (clusters). Tais agrupamentos sugerem que fatores relacionados ao espaço podem estar associados

às taxas de gravidez na adolescência, resultando em uma correlação espacial na distribuição observada.

2. Análise Inferencial (análogo ao item A.8.)

2.1. Ajuste do MRL e diagnóstico LM (análogo aos itens A.8.1.e A.8.3.)

Realizar o diagnóstico de Lagrange (LM) para detectar dependência espacial nos resíduos do MRL:

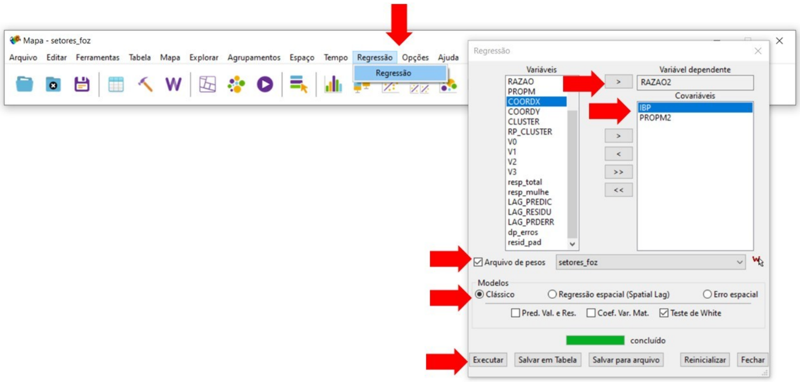

Figure 24: Clicando no ícone "Regressão" e em seguida em "Regressão" novamente. Configurando o modelo de regressão: escolhendo "RAZAO2" como variável dependente e "IBP" e "PROPM2" como covariáveis. Rodando o modelo clássico e selecionando a opção "Arquivo de pesos".

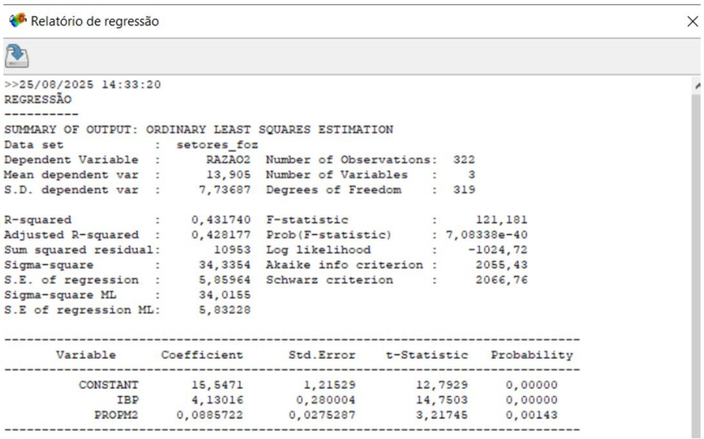

```
Relatório de regressão
>>25/08/2025 14:33:20
REGRESSÃO
----------
SUMMARY OF OUTPUT: ORDINARY LEAST SQUARES ESTIMATION
Data set            :  setores_foz
Dependent Variable  :      RAZAO2  Number of Observations:  322
Mean dependent var  :      13,905  Number of Variables   :    3
S.D. dependent var  :     7,73687  Degrees of Freedom    :  319

R-squared           :    0,431740  F-statistic           :     121,181
Adjusted R-squared  :    0,428177  Prob(F-statistic)     : 7,08338e-40
Sum squared residual:       10953  Log likelihood        :    -1024,72
Sigma-square        :     34,3354  Akaike info criterion :     2055,43
S.E. of regression  :     5,85964  Schwarz criterion     :     2066,76
Sigma-square ML     :     34,0155
S.E of regression ML:     5,83228

-----------------------------------------------------------------------------
       Variable      Coefficient      Std.Error    t-Statistic   Probability
-----------------------------------------------------------------------------
          CONSTANT       15,5471        1,21529        12,7929     0,00000
               IBP       4,13016       0,280004        14,7503     0,00000
            PROPM2     0,0885722      0,0275287        3,21745     0,00143
-----------------------------------------------------------------------------
```

Figure 25: Visualizando os resultados da regressão – parte 1

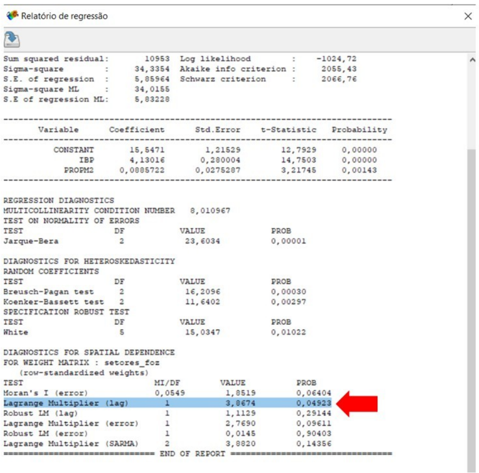

```
Relatório de regressão
Sum squared residual:       10953  Log likelihood        :    -1024,72
Sigma-square        :     34,3354  Akaike info criterion :     2055,43
S.E. of regression  :     5,85964  Schwarz criterion     :     2066,76
Sigma-square ML     :     34,0155
S.E of regression ML:     5,83228

-----------------------------------------------------------------------------
       Variable      Coefficient      Std.Error    t-Statistic   Probability
-----------------------------------------------------------------------------
          CONSTANT       15,5471        1,21529        12,7929     0,00000
               IBP       4,13016       0,280004        14,7503     0,00000
            PROPM2     0,0885722      0,0275287        3,21745     0,00143
-----------------------------------------------------------------------------

REGRESSION DIAGNOSTICS
MULTICOLLINEARITY CONDITION NUMBER   8,010967
TEST ON NORMALITY OF ERRORS
TEST                  DF          VALUE             PROB
Jarque-Bera            2           23,6034          0,00001

DIAGNOSTICS FOR HETEROSKEDASTICITY
RANDOM COEFFICIENTS
TEST                  DF          VALUE             PROB
Breusch-Pagan test     2           16,2096          0,00030
Koenker-Bassett test   2           11,6402          0,00297
SPECIFICATION ROBUST TEST
TEST                  DF          VALUE             PROB
White                  5           15,0347          0,01022

DIAGNOSTICS FOR SPATIAL DEPENDENCE
FOR WEIGHT MATRIX : setores_foz
   (row-standardized weights)
TEST                           MI/DF         VALUE          PROB
Moran's I (error)              0,0549         1,8519        0,06404
Lagrange Multiplier (lag)         1           3,8674        0,04923
Robust LM (lag)                   1           1,1129        0,29144
Lagrange Multiplier (error)       1           2,7690        0,09611
Robust LM (error)                 1           0,0145        0,90403
Lagrange Multiplier (SARMA)       2           3,8820        0,14356
============================== END OF REPORT ===============================
```

Figure 26: Visualizando os resultados da regressão – parte 2

No caso do estudo, o teste LM indicou que o melhor modelo a ser utilizado foi o modelo de defasagem espacial (SAR: Spatial Autoregression), que incorpora a dependência espacial nos dados. Esse modelo considera que o valor da variável resposta em um setor censitário é influenciado pelos valores em setores vizinhos, além das variáveis explicativas.

Ressalta-se que o valor de p para o teste do I de Moran dos resíduos do MRL no GeoDa corresponde à hipótese alternativa de que o I de Moran 0. Como o software ainda não disponibiliza a opção para a hipótese alternativa I de Moran > 0, consideramos, para fins de comparação, o valor de p dividido por dois (p = 0,032). Esse ajuste é válido porque o valor observado do I de Moran foi positivo e a distribuição gerada por randomização no GeoDa é simétrica, de modo que o resultado torna-se equivalente ao teste unicaudal realizado no R.

2.2. Ajuste do Modelo SAR (análogo ao item A.8.4.)

Ajustar o modelo SAR considerando as variáveis explicativas ‘IBP’ e ‘PROPM2’:

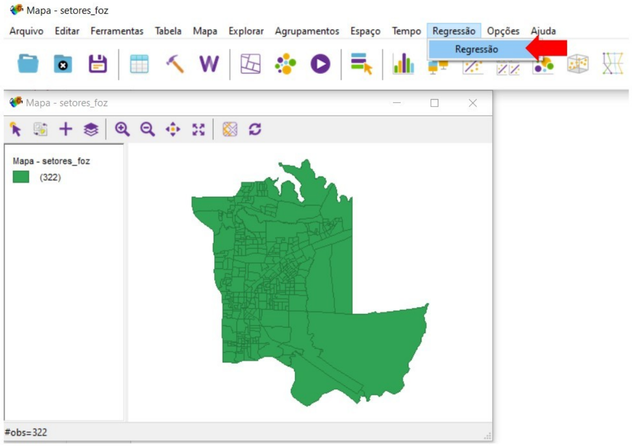

Figure 27: Clicando no ícone “Regressão” e em seguida em “Regressão” novamente.

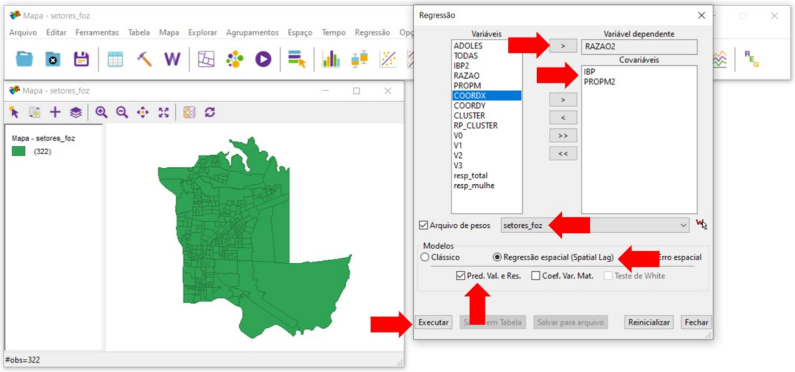

Figure 28: Configurando o modelo de regressão: mantendo a escolha de “RAZAO2” como variável dependente e “IBP” e “PROPM2” como covariáveis. Rodando o modelo regressão espacial (“Spatial Autoregression” ou “Spatial Lag”), mantendo a opção “Arquivo de pesos”.

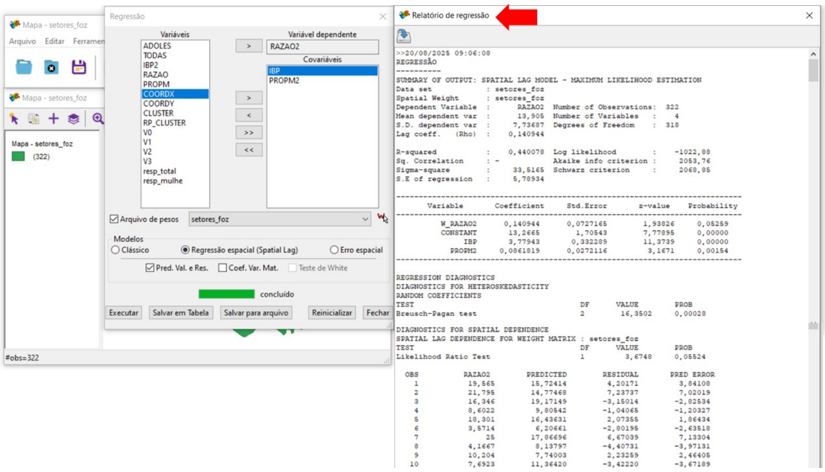

Figure 29: Visualizando os resultados da regressão

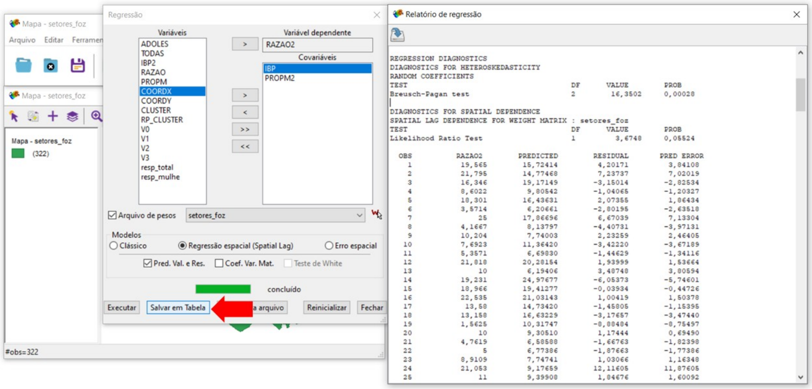

Figure 30: Salvando em tabela as colunas dos resíduos

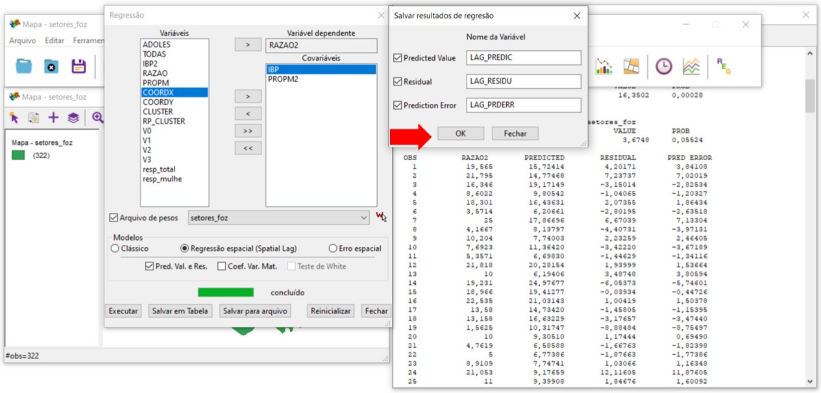

Figure 31: Confirmando o salvamento das variáveis

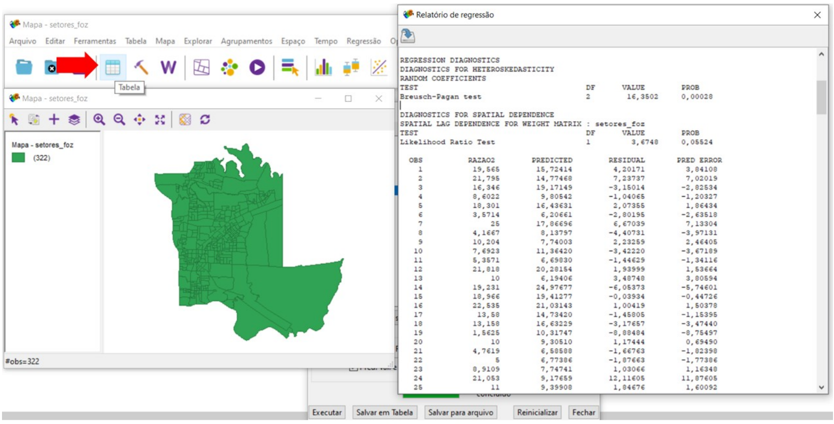

Figure 32: Abrindo a tabela de atributos

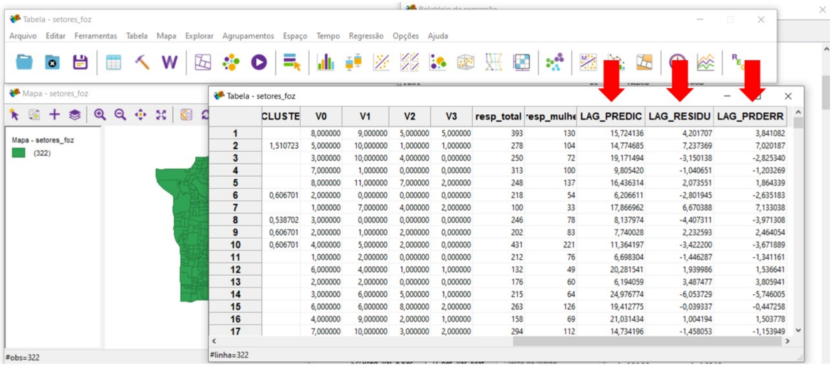

Figure 33: Visualizando as colunas na tabela

2.3. Análise de resíduos do modelo SAR (análogo ao item A.8.5.)

Criando a coluna dos resíduos padronizados. O cálculo é feito a partir da subtração da média dos resíduos (que se espera ser zero) dos resíduos estimados pelo modelo e, em seguida, pela divisão do resultado pela raiz quadrada da variância residual estimada (sigma quadrado).

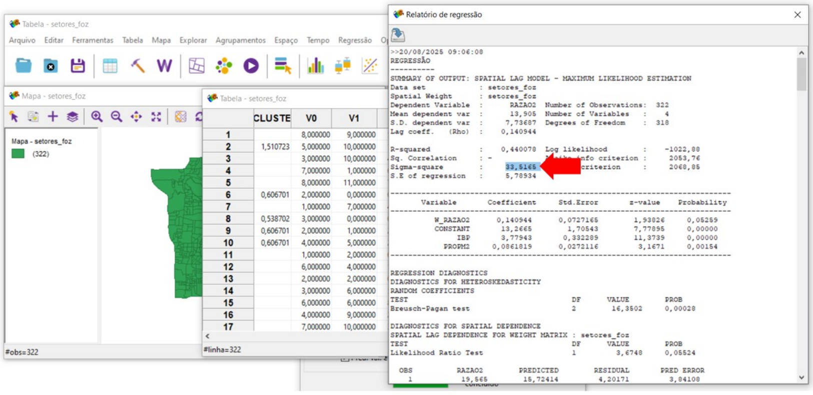

Figure 34: Copiando o valor do sigma quadrado do modelo

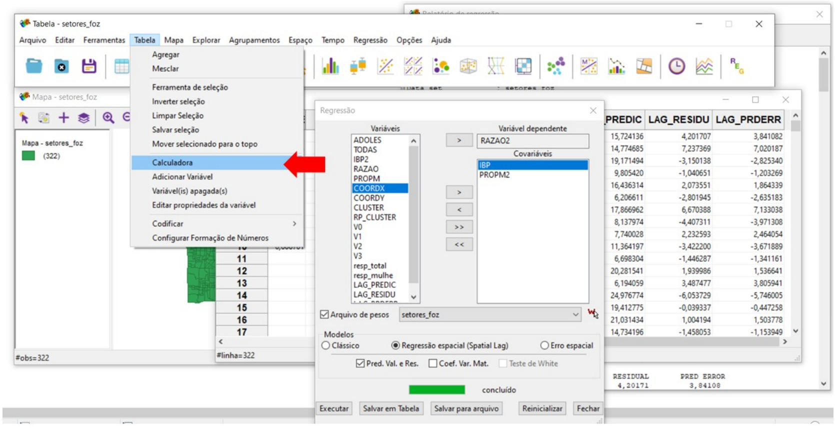

Figure 35: Abrindo a Calculadora no ícone "Tabela"

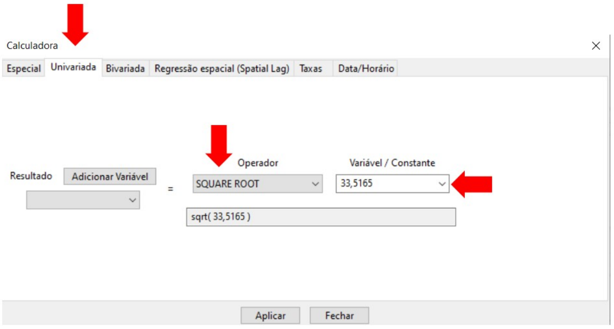

Figure 36: Na opção Univariada, aplicando o operador "Square Root" (raiz quadrada) ao valor do sigma quadrado copiado

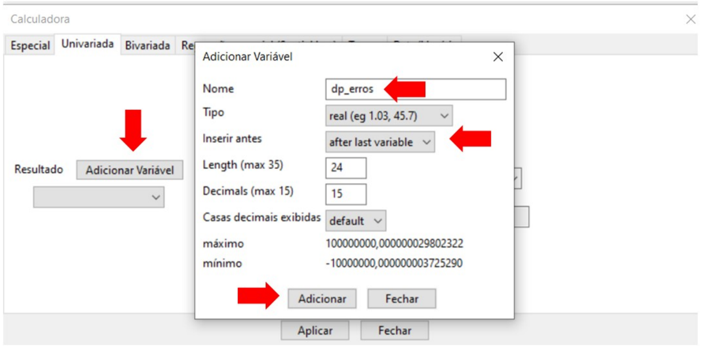

Figure 37: Clicando em "Adicionar variável" para criar a coluna "dp_erros". Selecionando a opção "after last variable" para inserir a coluna ao final da tabela

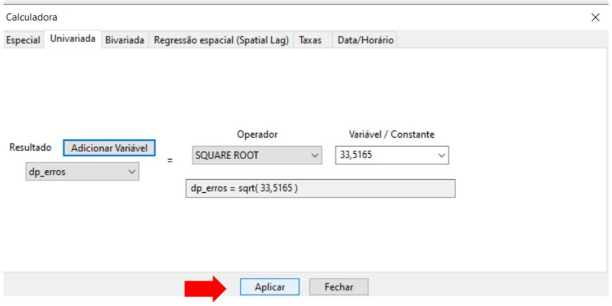

Figure 38: Clicando em "Aplicar"

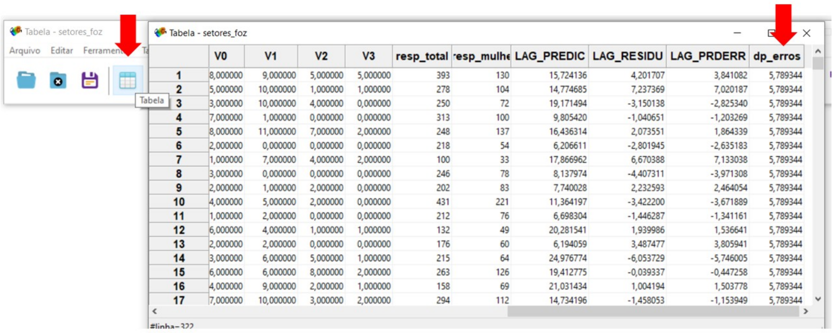

Figure 39: Abrindo a tabela para verificar se a nova variável foi adicionada

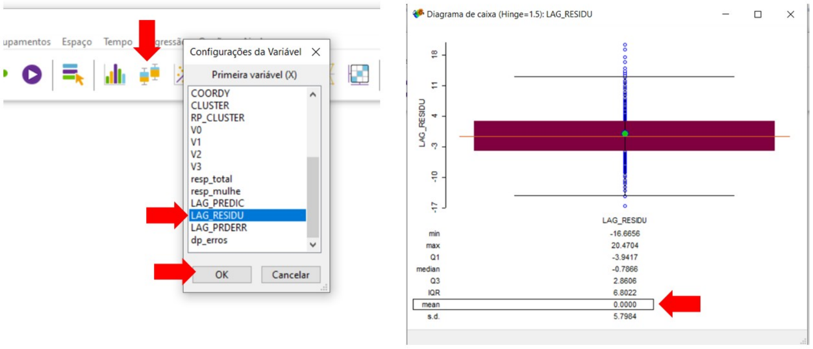

Figure 40: Verificando se a média dos resíduos é zero: clicando no ícone de boxplot, escolhendo a variável de resíduos e conferindo o valor de "mean"

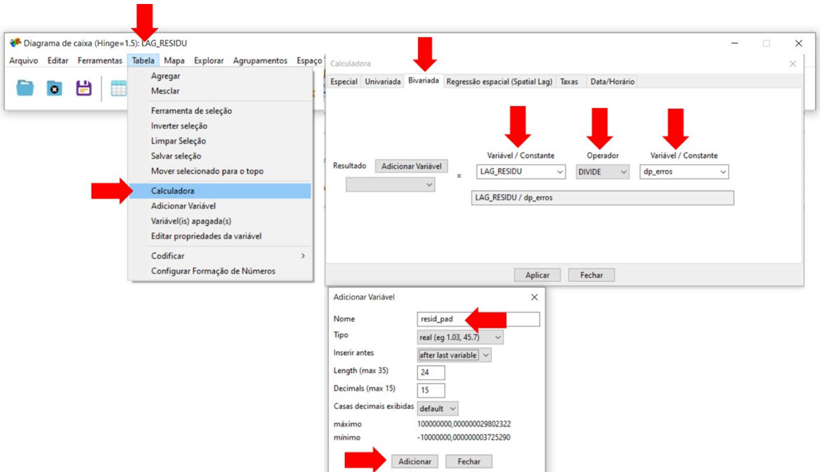

Figure 41: Abrindo a Calculadora, na opção Bivariada, e dividindo LAG_RESIDU pelo "dp_erros" (operador DIVIDE), criando a variável "resid_pad"

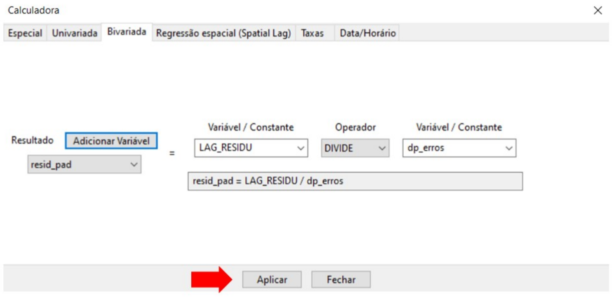

Figure 42: Clicando em "Aplicar"

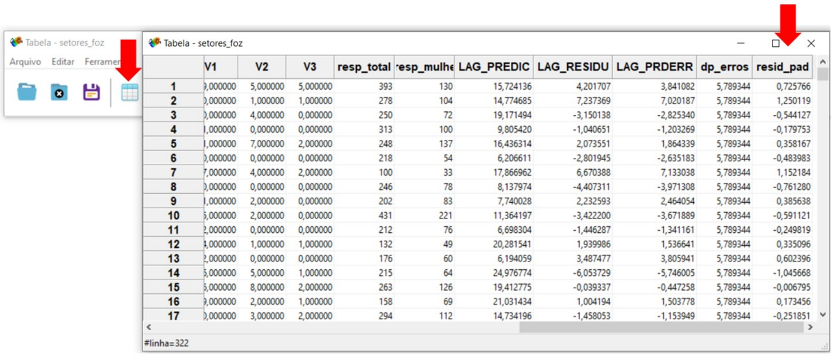

Figure 43: Verificando se a coluna dos resíduos padronizados foi criada na tabela

Diagrama de dispersão dos resíduos padronizados em função da covariável Índice Brasileiro de Privação:

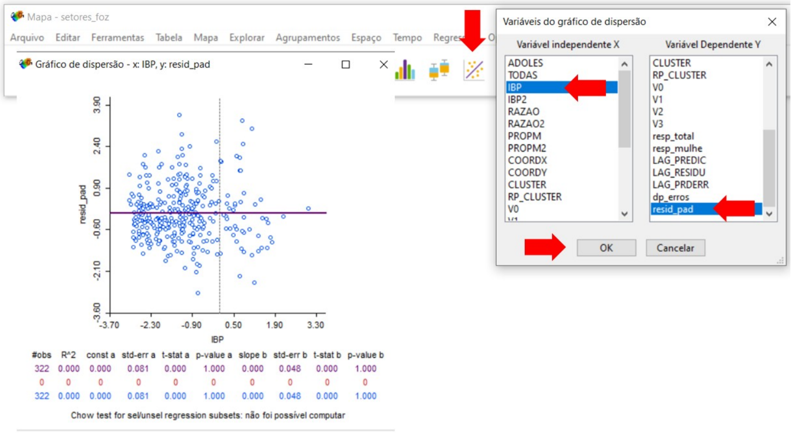

Figure 44: Fazendo o scatterplot dos resíduos padronizados em relação ao IBP

Diagrama de dispersão dos resíduos padronizados em função da covariável Proporção de mulheres responsáveis pelo domicílio:

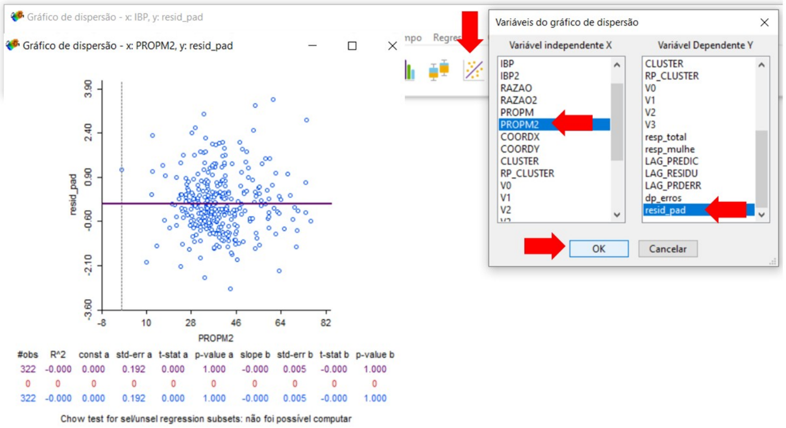

Figure 45: Fazendo o scatterplot dos resíduos padronizados em relação ao PROPM2

Diagrama de dispersão dos resíduos padronizados em função dos valores ajustados:

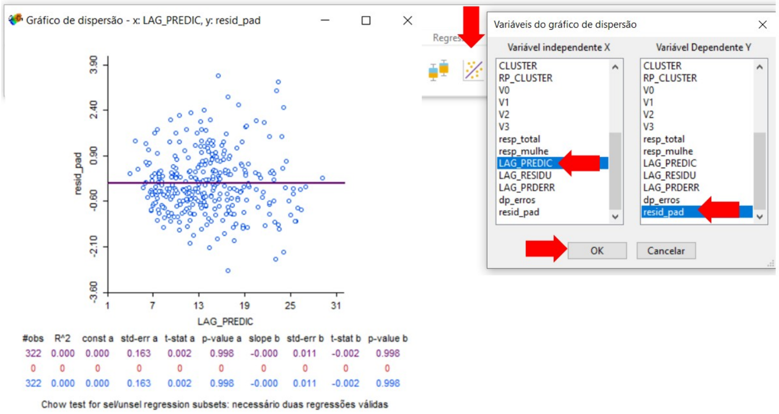

Figure 46: Fazendo o scatterplot dos resíduos padronizados em relação aos valores preditos

Histograma dos resíduos padronizados:

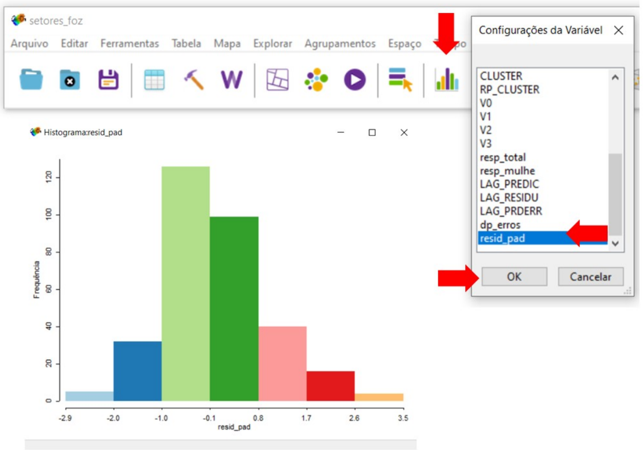

Figure 47: Fazendo o histograma dos resíduos padronizados

Teste de Moran para os resíduos do modelo:

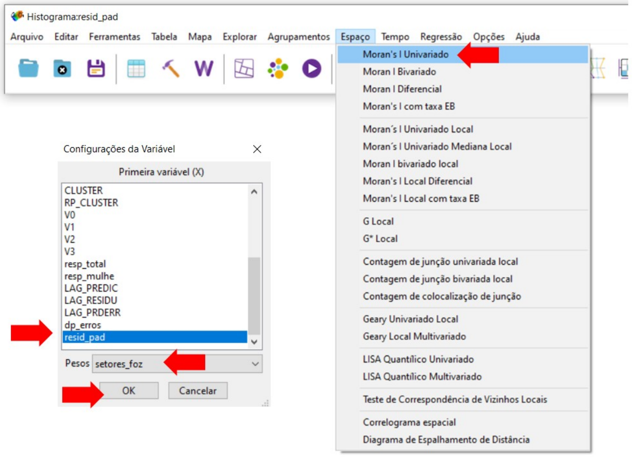

Figure 48: Calculando o Moran Global univariado dos resíduos padronizados

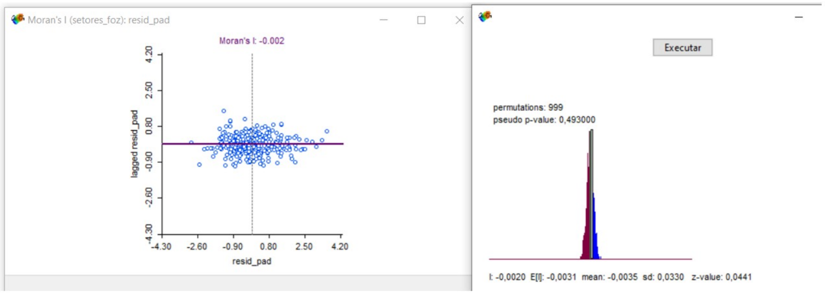

Figure 49: Visualizando o valor do Moran e o pseudo p-valor

De acordo com o teste de Moran, os resíduos do modelo não apresentam autocorrelação espacial significativa (Moran's I = -0.002, p = 0.487). Isso indica que, após o ajuste, a dependência espacial foi eliminada.
