# Supplementary material for: Linear regression in ecological studies involving space: methodology and an application example in public health
Source: Rev Bras Epidemiol. 2026 Apr 20;29:e260018. doi: 10.1590/1980-549720260018 (PMC13101397; doi:10.1590/1980-549720260018)
Supplement: Translation

# Translation: Regressão linear em estudos ecológicos envolvendo o espaço: metodologia e exemplo de aplicação na área da saúde

**DOI:** 10.1590/1980-549720260018.2

**Authors:** Gleice Margarete de Souza Conceição, Patricia Marques Moralejo Bermudi, Raquel Gardini Sanches Palasio, Gerson Laurindo Barbosa, Camila Meireles Fernandes, Lidia Maria Reis Santana, Ligia Vizeu Barrozo, Daiane Leite da Roza, José Alberto Quintanilha, Francisco Chiaravalloti-Neto

**Affiliations:** I Universidade de São Paulo, Faculdade de Saúde Pública, Departamento de Epidemiologia - São Paulo (SP), Brasil.; II Instituto Pasteur, Secretaria da Saúde do Estado de São Paulo - São Paulo (SP), Brasil.; III Universidade Federal de São Paulo, Departamento de Medicina - São Paulo (SP), Brasil.; IV Universidade de São Paulo, Faculdade de Filosofia, Letras e Ciências Humanas - São Paulo (SP), Brasil.; V Universidade de São Paulo, Instituto de Energia e Ambiente - São Paulo (SP), Brasil.

**Keywords:** Regressão linear, Estudos ecológicos, Dependência espacial, Análise espacial, Autocorrelação espacial, Modelos autorregressivos

## Abstract

Muitos fenômenos em saúde podem ser melhor compreendidos se considerada a região geográfica em que ocorrem. Um dos pontos mais importantes a ser considerado em delineamentos espaciais é a presença de autocorrelação em observações medidas ao longo do espaço. Se esta dependência não for adequadamente modelada, as estatísticas obtidas poderão ser viesadas, comprometendo a validade de conclusões sobre a presença ou ausência de associações. Metodologias desenvolvidas com base no modelo de regressão linear permitem acomodar adequadamente essa dependência, gerando estimativas precisas, robustas e não viesadas. Com o objetivo de ressaltar a aplicabilidade de modelos espaciais e apontar os cuidados necessários na análise dos dados, este artigo descreve, passo a passo, uma das metodologias mais comumente utilizadas para a análise de dados espaciais, bem como os cuidados a serem tomados para evitar erros na modelagem e distorção dos resultados. São apresentados o modelo de regressão linear, os procedimentos para avaliar a qualidade do ajuste, a medida mais utilizada para detectar a presença de dependência espacial e dois modelos autorregressivos comumente aplicados para modelar esta dependência (SAR e SEM). Um exemplo de aplicação é apresentado, utilizando-se os softwares GeoDa e R.

## INTRODUÇÃO

O termo “regressão” surgiu em 1885, com Francis Galton. Ao estudar características antropométricas de sucessivas gerações, Galton observou que filhos de pais com estatura alta em relação à média tendem a ser altos, porém mais baixos que seus pais; e filhos de pais com estatura baixa em relação à média tendem a ser baixos, porém mais altos que seus pais - postulando, então, que a estatura dos corpos humanos tendia a “regredir” à média^1^.

O Método dos Mínimos Quadrados, desenvolvido por Legendre e Gauss para determinar a órbita dos cometas, possibilitou estimar os parâmetros da regressão proposta por Galton. Gauss apontou que o método fornece as melhores estimativas quando se parte do princípio de que os erros são aleatórios, independentes e seguem a distribuição da curva normal^2^ ^,^ ^3^. Tal metodologia foi aperfeiçoada e utilizada na formulação da teoria dos Modelos de Regressão Linear (MRL). Os avanços tecnológicos reduziram tempo e esforço computacional, permitindo a aplicação da técnica para conjuntos cada vez maiores de dados. Atualmente, os MRL são utilizados para a análise de dados nas mais diversas áreas do conhecimento.

Com base nessa teoria, novas metodologias foram desenvolvidas, como os modelos para análise de dados espaciais e temporais. Estes podem ajudar a estabelecer a relação entre variáveis de interesse e fatores inter-relacionados ao longo do tempo e no espaço, permitindo uma compreensão aprofundada dos fenômenos estudados e possibilitando a formulação de políticas públicas mais eficazes^4^.

É comum que observações medidas no espaço e/ou no tempo estejam correlacionadas, sendo necessário levar essa dependência em conta. Modelos de regressão espacial permitem incorporar essa dependência, gerando estimativas precisas, robustas e não viesadas, além da identificação e interpretação dos efeitos dos fatores espaciais que influenciam o fenômeno em estudo.

Por outro lado, desconsiderar a dimensão espacial pode comprometer a validade das conclusões. Entre os principais problemas decorrentes disso estão a especificação inadequada do modelo, a omissão de variáveis importantes, a obtenção de estimativas viesadas e de intervalos de confiança e p-valores incorretos, podendo levar à obtenção de associações espúrias e a conclusões imprecisas ou inválidas^5^.

Assim, os modelos de regressão espacial são ferramentas poderosas para análise de dados observados no espaço. Softwares, como GeoDa e a linguagem R, têm facilitado sua aplicação. Contudo, é importante destacar que o sucesso dessas aplicações depende do conhecimento dos fundamentos teóricos e da compreensão do problema investigado.

Embora a literatura científica internacional contenha estudos com abordagem espacial, ela é menos frequente nas revistas brasileiras. Uma busca na Biblioteca Virtual em Saúde (BVS), Saúde Pública Brasil^6^, com o termo “regressão linear” revelou a existência de 2.458 artigos em português publicados até agosto de 2025. Ao restringir a busca para “regressão linear” e “espacial”, o número é reduzido para apenas 82.

Conforme Figueiredo Filho et al.^7^, materiais de apoio que contribuam para o entendimento de modelos espaciais e espaço-temporais, destacando procedimentos adequados e práticas a evitar, são igualmente escassos nas revistas científicas brasileiras. Nosso estudo se propõe a preencher essa lacuna, oferecendo uma abordagem abrangente sobre a importância e aplicação de modelos espaciais. O objetivo é divulgar ferramentas que auxiliem na escolha do método mais adequado para a análise de dados espaciais, bem como os vieses que podem surgir quando a dependência espacial é ignorada, reduzindo o risco de conclusões equivocadas e avançando no uso da regressão em dados espaciais. Para tanto, apresentamos o MRL e suas suposições, a medida mais utilizada para detectar a dependência espacial nos resíduos do modelo e duas das abordagens mais utilizadas para modelar esta dependência. Como exemplo, apresentamos uma aplicação utilizando os dados de Fernandes et al.^8^, que avaliaram a distribuição da prevalência de mães adolescentes no espaço e sua relação com indicadores socioeconômicos no município de Foz do Iguaçu (PR).

Por se tratar de um estudo metodológico, sem envolvimento direto de seres humanos ou utilização de dados individuais identificáveis, não houve necessidade de submissão ao Comitê de Ética em Pesquisa.

## O MODELO DE REGRESSÃO LINEAR

Os MRL são utilizados para descrever e quantificar associações lineares ou linearizáveis e, também, para o cálculo de previsões. Podem ser utilizados na avaliação de tendências em séries espaciais, temporais ou espaço-temporais. Envolvem uma variável resposta quantitativa (*Y*), preferencialmente contínua, e uma ou mais variáveis explicativas (*X*_*1*_ , *X*_*2*_ ,..., *X*_*p*_ ), também denominadas covariáveis, que podem ser quantitativas ou qualitativas. O objetivo é avaliar o efeito que as covariáveis exercem conjuntamente na Variável Resposta (VR) e aproximá-lo por uma função matemática, facilitando sua descrição e quantificação e, eventualmente, fazer previsões^9^.

Tais modelos podem ser aplicados em delineamentos ecológicos espaciais, nos quais a região geográfica dos eventos é fundamental para compreender o fenômeno estudado. Assim, tanto a estrutura do banco de dados quanto o modelo adotado devem considerar a distribuição espacial dos dados. Nesse tipo de delineamento, a área de estudo é particionada em Unidades Espaciais de Área (UEA) que, em geral, baseiam-se em unidades pré-existentes, como municípios e setores censitários.

O MRL para a análise de dados espaciais é semelhante ao modelo clássico, exceto que as variáveis são medidas por UEA e cada linha do banco de dados refere-se a uma delas. O modelo pode ser escrito como^10^

Y=Xβ+ε

Onde:

*Y* = (*y*_*1*_ , *y*_*2*_ ,..., *y*_*N*_ ) é o vetor *(Nx1)* que contém os valores da VR em cada UEA;

N é o número de UEAs;

*X* é a matriz *(Nxp)* contendo os valores das *p* covariáveis em cada UEA;

β é o vetor de parâmetros *(px1)* a serem estimados, que quantificarão o efeito de cada variável explicativa na resposta;

ε é um vetor *(Nx1)* de erros aleatórios.

As suposições do modelo são que ε~*N*(0,σ^2^ *I*_*N*_ ), em que *I*_*N*_ é a matriz identidade e (ε_*i*_ , ε_*j*_ ) são independentes para todo par de UEAs (*i, j)*, e podem ser traduzidas em quatro condições essenciais: normalidade: os erros aleatórios com distribuição Normal, implicando na normalidade da VR; homoscedasticidade: a variância da VR constante ao longo dos valores das covariáveis; linearidade: relação linear entre a VR e as covariáveis; independência: valores da VR independentes entre si (não correlacionados)^10^.

Tais suposições devem ser checadas antes e depois do ajuste do modelo, nas análises exploratória e de resíduos, respectivamente. Se não satisfeitas, os resultados estarão comprometidos. Na ausência de linearidade, o modelo não refletirá o comportamento dos dados, e as estimativas serão inválidas. Na ausência de normalidade, heteroscedasticidade ou independência, as variâncias dos estimadores, as estatísticas e os p-valores dos testes de hipóteses serão viesados, comprometendo quaisquer conclusões sobre a presença ou ausência de associações. Finalmente, a presença de *outliers* pode resultar em estimadores e variâncias viesadas, se sua influência for grande^10^.

Para contornar problemas de normalidade, linearidade e homoscedasticidade, as seguintes medidas podem ser empregadas: transformações na VR (como a logarítmica); ou o ajuste de modelos lineares generalizados, aditivos generalizados ou bayesianos. Estes consideram outras distribuições, além da normal, como Poisson e binomial negativa, e outras formas para a relação entre a VR e as covariáveis, além da linear^11^. Se as observações não forem independentes e os dados não permitirem especificar adequadamente a estrutura de dependência espacial, pouco pode ser feito, o que compromete a realização de qualquer análise. Por outro lado, havendo essa estrutura (por exemplo, ao longo do espaço), modelos que incorporam essa dependência devem ser utilizados. Os modelos com dependência espacial fazem parte desta categoria.

Em estudos espaciais, é comum a atuação da primeira lei da Geografia^12^: no espaço, as coisas estão relacionadas, mas as espacialmente mais próximas estão mais relacionadas do que as mais distantes. Esse fenômeno é denominado dependência espacial ou autocorrelação espacial positiva^4^.

É possível que desvios das suposições de normalidade, homoscedasticidade e a presença de dependência espacial na VR sejam corrigidos após a inclusão das covariáveis. Se o resíduo do modelo ajustado for normal, aleatório, homoscedástico, com poucos *outliers* (desde que não distorçam as estimativas dos coeficientes) e livre de dependência espacial, o modelo estará bem ajustado. Um exemplo é o estudo de Diniz et al.^13^, que modelou espacialmente a mortalidade por câncer de mama nos municípios de São Paulo. Apesar de a taxa ter apresentado dependência espacial, ela foi convenientemente acomodada pelas covariáveis, e os resíduos não apontaram nenhum desvio das suposições do MRL.

## ANÁLISE EXPLORATÓRIA

O primeiro passo de qualquer análise estatística é a exploração dos dados; ela deve ser minuciosa e anteceder a aplicação de qualquer modelo. É necessário entender a relação da VR com cada covariável e as relações das covariáveis entre si. A seguir, apresentamos as etapas que devem ser contempladas em uma análise exploratória tendo em vista o ajuste de qualquer MRL, de modo a evitar erros habitualmente cometidos.

Inicialmente, descreve-se cada variável individualmente, de acordo com a sua natureza. Variáveis qualitativas devem ser descritas por meio de tabelas de frequência e gráficos em barras. Para variáveis quantitativas utilizam-se medidas-resumo numéricas (média, mediana, desvio-padrão etc.) e uma série de gráficos (histogramas, box-plots e dotplots). Estes fornecem informações sobre a faixa de valores assumida e sua frequência, distribuição, tendência central e variabilidade, presença de assimetrias e caudas. Se existirem valores aberrantes (*outliers*), é essencial avaliar sua influência nas estimativas do MRL e, se necessário, utilizar medidas de controle apropriadas - eles devem ser controlados, mas nunca deletados. Para avaliar a aderência dos dados a uma determinada distribuição, além do histograma e de testes de normalidade, *Qq-plots* devem ser utilizados. Somente após compreender o comportamento de cada variável deve-se descrever seu comportamento conjunto, duas a duas, e avaliar a presença de associações, tanto da VR com cada covariável quanto das covariáveis entre si.

Para descrever a relação da VR com as covariáveis qualitativas a resposta deve ser descrita segundo as categorias da qualitativa, utilizando as medidas-resumo numéricas e os gráficos citados anteriormente. Em particular, é conveniente que a variância da VR seja semelhante nas categorias das covariáveis qualitativas. Isso pode ser avaliado por meio de testes de igualdade de variâncias^10^.

Para covariáveis quantitativas deve-se observar conjuntamente o diagrama de dispersão e o coeficiente de correlação. O primeiro exibirá a forma da relação e, se for linear, o segundo expressará a intensidade da relação. Dois pontos devem ser considerados. O primeiro é a linearidade, uma das suposições do MRL. Se a forma da relação entre a VR e a covariável não for linear, o MRL não será aplicável. Neste caso, pode ser conveniente transformar a variável quantitativa em qualitativa (por exemplo, utilizar faixas etárias em vez da idade). Os valores que definirão as categorias dependem do conhecimento do pesquisador e, se não houver conhecimento prévio, é conveniente defini-los de forma que cada categoria contenha mais ou menos o mesmo número de observações. Outra alternativa é a utilização de polinômios ou alisadores^10^ ^,^ ^14^. O segundo ponto refere-se ao coeficiente de correlação medir apenas associações lineares. Este deve ser observado em conjunto com o diagrama de dispersão, nunca isoladamente. Ainda, um coeficiente alto não implica em associação linear; do mesmo modo, um coeficiente baixo não implica necessariamente em ausência de associação, uma vez que pode ocorrer quando a associação não for linear.

É necessário, ainda, entender a relação das covariáveis entre si (usando as mesmas ferramentas previamente descritas), além de avaliar a presença de colinearidade, que ocorre quando elas são fortemente correlacionadas. Covariáveis colineares tornam os coeficientes instáveis e produzem estimativas pouco robustas. Por exemplo, informações sobre renda e escolaridade podem ser colineares: quanto maior a escolaridade, maior a renda. Quando se conhece uma delas, as informações adicionais fornecidas pela outra podem ser irrelevantes. Um critério possível seria considerar duas variáveis colineares se a correlação entre elas for maior que 0,70 (ou menor do que -0,70)^15^ e descartar uma delas. Procedimentos de modelagem *stepwise forward* ^*16*^ oferecem critérios robustos para a inclusão e exclusão de covariáveis colineares, desde que aplicados adequadamente: o critério de inclusão/permanência de variáveis nunca deve ser exclusivamente a significância estatística, mas todo um conhecimento do pesquisador sobre as relações conceituais e a plausibilidade biológica. Além disso, as decisões nunca devem ficar a critério do software - sempre do pesquisador.

Também é essencial avaliar a presença de interações entre covariáveis, tema não tratado neste artigo. Entretanto, é fundamental ressaltar que a omissão de uma interação importante tornará o modelo incorretamente especificado, levando a conclusões equivocadas.

Finalmente, deve-se avaliar se a distribuição da VR é aleatória no espaço ou se há dependência espacial. Isso pode ser feito por meio de índices que medem o grau de autocorrelação espacial, sendo o índice global de Moran, apresentado adiante, o mais utilizado.

Zuur et al.^17^ propõem um protocolo para a análise exploratória utilizando o R, que pode ser útil, mas insuficiente, para a realização da análise exploratória.

## ANÁLISE DE RESÍDUOS

É feita após o ajuste do modelo para verificar se o modelo adotado é apropriado aos dados. Para tanto, é necessário investigar seus resíduos. O resíduo (*e*_*i*_ ) é definido como

$$e_{i}=Y_{i}-{\hat{Y}}_{i}$$

Isto é, a diferença entre o valor observado (*Y*_*i*_ ) e o correspondente valor ajustado (*Ŷ*_*i*_ ).

O resíduo *e*_*i*_ pode ser visto como o erro observado, em contrapartida ao verdadeiro erro ε_*i*_ , que é desconhecido e não observável. O MRL supõe que os ε_*i*_ são independentes e identicamente distribuídos, com distribuição *N*(0,σ^2^). Se o modelo ajustado for apropriado, os *e*_*i*_ devem refletir as propriedades assumidas para os ε_*i*_ , ou seja, devem ter distribuição *N*(0,σ^2^), sem *outliers,* e serem independentes (sem autocorrelação). A análise dos resíduos envolve a construção de gráficos e medidas numéricas, fundamentalmente:

1. Diagrama de dispersão dos resíduos versus cada covariável;
2. Diagrama de dispersão dos resíduos versus valores ajustados;
3. Histogramas dos resíduos;
4. QQ-plot;
5. Índice de Moran.

Deve-se utilizar resíduos padronizados, por serem mais eficientes do que os brutos, principalmente na detecção de outliers. Nos diagramas listados como itens 1 e 2, os resíduos devem estar dispersos aleatoriamente em torno de zero, com variância constante, sem padrões sistemáticos e sem outliers influentes, com pelo menos 95% dos valores situados entre ±1,96. Os itens 3 e 4 (histograma e QQ-plot) devem apresentar distribuição aproximadamente normal. O Índice de Moran, listado como item 5, deve indicar a ausência de autocorrelação espacial.

## ÍNDICE DE MORAN GLOBAL (I DE MORAN)

O I de Moran^18^ mede a correlação entre os valores de uma variável com seus valores nas vizinhanças. Cliff e Ord^19^ foram os primeiros a aplicá-lo à análise de resíduos de regressão. Para construí-lo, é necessário identificar, para cada UEA, seus respectivos vizinhos. A definição de vizinhança pode ser estabelecida por contiguidade ou distância. A primeira é mais utilizada em delineamentos ecológicos e considera como vizinhos de uma UEA as áreas que compartilham um lado comum (*rook* ou torre) ou com um lado ou um vértice comum (*queen* ou rainha). A vizinhança por distância considera a distância entre o centroide de uma UEA e os das áreas vizinhas e pode ser construída de duas formas: considerando como vizinhas todas as unidades em uma distância máxima previamente fixada; ou considerando como vizinhas as k unidades mais próximas. Ambos os parâmetros são definidos pelo pesquisador.

O índice pode ser construído utilizando-se a VR ou os resíduos do MRL ajustado, e é calculado como:

$$I=\frac{N}{W}\frac{\sum_{i=1}^{N}\sum_{j=1}^{N}w_{ij}\left(y_{i}-\overset{-}{Y}\right)\left(y_{j}-\overset{-}{Y}\right)}{\sum_{i=1}^{N}{\left(y_{i}-\overset{-}{Y}\right)}^{2}}$$

Onde:

N é o número de UEAs;

*y*_*i*_ e *y*_*j*_ são os valores da VR (ou do resíduo do modelo de regressão) para o par (i, j) de UEAs, *i*=1,...,*N*, *j*=1,...,*N* ;

*W*_*ij*_ são os pesos espaciais, assumem o valor 1 se *i* e *j* são UEAs vizinhas, e o valor 0, em caso contrário;

$W=\sum_{i=1}^{N}\sum_{j=1}^{N}w_{ij}$, isto é, a soma dos pesos *W*_*ij*_

$\overset{-}{Y}=\frac{\sum_{i=1}^{N}y_{i}}{N}$ a média amostral de *Y*.

Where:

N is the number of SAUs;

*y*_*i*_ and *y*_*j*_ are the values of the RV (or of the regression model residual) for the pair (i, j) of SAUs, *i*=1,...,*N*, *j*=1,...,*N*;

*W*_*ij*_ are the spatial weights, which take the value 1 if *i* and *j* are neighboring SAUs, and 0, otherwise;

Outras possibilidades para os pesos *W*_*ij*_ podem ser utilizadas, por exemplo, , em que *l*_*ij*_ é o comprimento da fronteira entre *A*_*i*_ e *A*_*j*_ e *l*_*i*_ é o perímetro de *A*_*i*_ .

O I de Moran assume valores entre -1 e 1. Valores próximos de zero sugerem ausência de autocorrelação espacial, indicando que a VR (ou o resíduo) está aleatoriamente distribuída no espaço, sem padrões de agrupamento. Valores maiores do que zero apontam para a existência de autocorrelação espacial positiva, indicando que áreas com valores para a VR (ou resíduo) altos estão geograficamente próximas umas das outras, assim como áreas com valores baixos. Valores menores do que zero apontam para uma autocorrelação espacial negativa, indicando que áreas com valores semelhantes estão dispersas, distantes umas das outras. O teste de hipóteses para o índice (em que Ho significa ausência de autocorrelação espacial) é realizado com base na estratégia de Monte Carlo, utilizando um grande número de simulações para obtenção do p-valor^4^. Se o índice de Moran aplicado aos resíduos do MRL apontar a existência de dependência espacial, será necessário ajustar um modelo que possa acomodá-la.

Além disso, é importante distinguir o uso do índice de Moran aplicado à VR daquele aplicado aos resíduos. O I de Moran da variável resposta identifica autocorrelação espacial prévia ao ajuste múltiplo, enquanto o I de Moran dos resíduos avalia se ainda há autocorrelação após o ajuste. Assim, um Moran significativo nos resíduos indica que o modelo múltiplo não capturou completamente a estrutura espacial e, ainda, que um modelo espacial pode ser necessário.

## MRL COM DEPENDÊNCIA ESPACIAL GLOBAL

A dependência espacial pode ter caráter local ou global. Se a autocorrelação espacial é heterogênea, isto é, varia de local para local, a dependência tem caráter local, sendo necessário ajustar um modelo com múltiplos parâmetros para acomodá-la. A regressão geograficamente ponderada^20^ é uma das abordagens possíveis, mas se não houver grande heterogeneidade pode-se assumir que a dependência tem caráter global, podendo ser acomodada por meio de um único parâmetro, adicionado ao MRL. Neste artigo, trataremos apenas de modelos com efeitos globais. Os dois mais comumente utilizados são os modelos autorregressivos SAR (do inglês *Spatial Autoregression*) e SEM (do inglês *Spatial Error Model*). No primeiro, a dependência é explicitamente modelada na VR, incorporando um termo de defasagem espacial; no segundo, é considerada um ruído a ser removido e tratada como um componente dos resíduos^21^ ^,^ ^22^.

O SAR é formulado como:

Y=pWY+Xβ+ε

Onde:

*Y*, *X*, β, ε são os mesmos previamente definidos;

*W* é a matriz de vizinhança contendo os pesos *W*_*ij*_ definidos anteriormente

(*W*_*ij*_ =1, se i e j são UEAs vizinhas e *W*_*ij*_ =0, caso contrário);

ρ é o coeficiente espacial autorregressivo a ser estimado.

A dependência espacial será acomodada pelo termo ρ*WY* , em que *WY* corresponde à VR espacialmente defasada e ρ, ao coeficiente espacial autorregressivo, associado à defasagem.

O SEM é formulado como:

Y=Xβ+u

u=λWu+ε

Onde:

*Y*, *X*, β, *W,* ε são os mesmos previamente definidos;

λ é o coeficiente espacial autorregressivo a ser estimado.

Aqui, a dependência espacial é acomodada no termo de erro *u*, decomposto em λ*Wu* (erro espacialmente defasado que representa a parte dos resíduos com dependência espacial) e ε (erros normalmente distribuídos, com variância constante e independentes).

Nesses modelos, valores de ρ ou λ próximos de zero indicam ausência de autocorrelação espacial; por sua vez, valores maiores que zero e estatisticamente significativos indicam dependência espacial.

A escolha do modelo mais adequado é feita utilizando-se o Diagnóstico de Lagrange (LM), testando-se as hipóteses H_*0*ρ**_: *ρ*=0 e H_*0*λ**_: λ=0. As estatísticas dos testes (*LM*_*ρ*_ e *LM*_*λ*_ , respectivamente) são obtidas por meio de multiplicadores de Lagrange^22^. Se ambas as hipóteses forem rejeitadas, utilizam-se estatísticas robustas, e o modelo mais adequado será aquele com o maior valor da estatística LM robusta (*RLM*_*ρ*_ ou *RLM*_*λ*_ ).

Uma vez determinado o modelo mais adequado, uma nova análise de resíduos será necessária, conforme os itens 4 e 5. Se o I de Moran ainda apontar a presença de dependência espacial nos resíduos, então os parâmetros ρ ou λ não foram capazes de capturar toda a dependência. Antes de buscar novos tipos de modelagens, recomenda-se escolher uma matriz de vizinhança mais adequada ou incluir covariáveis inicialmente omitidas.

Esses procedimentos são apresentados na Figura 1.

**Figura 1.** Fluxo dos procedimentos para o ajuste de um MRL com componente espacial global^22^.

MRL: Modelo de Regressão Linear; SAR: *Spatial Autoregression;* SEM: *Spatial Error Model*

## EXEMPLO DE APLICAÇÃO

O estudo de Fernandes et al.^8^ utilizou como UEAs os setores censitários urbanos do município de Foz do Iguaçu (dados disponíveis em https://doi.org/10.6084/m9.figshare.27310467.v2)^23^. Após a análise exploratória, foi ajustado um MRL, tendo como VR a prevalência de mães adolescentes e, como covariáveis, o Índice Brasileiro de Privação (IBP) e a Proporção de Mulheres Responsáveis pelo Domicílio (PMRD). O I de Moran (estimado com matriz de vizinhança *Queen*) para os resíduos foi 0,059 (p=0,025), indicando dependência espacial.

Com base no diagnóstico ML, optou-se pelo ajuste de um SAR. A nova análise de resíduos mostrou um modelo bem ajustado, e o I de Moran (0,011, p=0,915) indicou que a dependência espacial foi adequadamente modelada. As estimativas de ambos os modelos estão na Tabela 1. Nota-se, no SAR, a diminuição do efeito do IBP e o aumento da amplitude de seu intervalo de confiança. Os procedimentos de modelagem, passo a passo, utilizando os softwares GeoDa^22^ e os códigos utilizados no R^24^, são apresentados no material suplementar (https://github.com/LAES-USP/material-supl-regressao-espacial.git).

**Tabela 1.** Estimativas dos modelos Modelos de Regressão Linear e *Spatial Autoregression* para a prevalência de mães adolescentes. Foz do Iguaçu (PR), Brasil, 2013-2019.

Variável explicativa	MRL			SAR		
	β	IC (β; 0,95)	Valor de p	β	IC (β; 0,95)	Valor de p
Índice Brasileiro de Privação	4,13	(3,58; 4,68)	0,000	3,77	(3,13; 4,43)	0,000
Proporção de mulheres responsáveis pelo domicílio	0,09	(0,03; 0,14)	0,001	0,09	(0,03; 0,14)	0,000
Coeficiente espacial autorregressivo (ρ)	-	-	-	0,14	(0,00; 0,28)	0,055
**Medida**	**Valor**		**Valor de p**	**Valor**		**Valor de p**
I de Moran	0,059		0,032	0,011		0,915
R^2^	0,43		-	0,44		-
AIC	2057,4		-	2055,8		-

MRL: Modelos de Regressão Linear; SAR: *Spatial Autoregression*.

## Declaração de disponibilidade de dados

O conjunto de dados que dá suporte aos resultados deste estudo está publicamente disponível no repositório figshare (https://doi.org/10.6084/m9.figshare.27310467.v2).

O material suplementar do artigo está disponível no repositório GitHub (https://github.com/LAES-USP/material-supl-regressao-espacial.git). O conteúdo inclui a descrição detalhada dos procedimentos de modelagem espacial, os comandos em R (versão 4.3) e as ilustrações geradas no GeoDa (versão 22), além dos arquivos necessários para a reprodução das análises.

## Material suplementar

PDF suplementar
